# Mission, Organization and Future Direction of the Serological Sciences Network for COVID-19 (SeroNet) Epidemiologic Cohort Studies

**DOI:** 10.1093/ofid/ofac171

**Published:** 2022-04-27

**Authors:** Jane C Figueiredo, Fred R Hirsch, Lawrence H Kushi, Wendy N Nembhard, James M Crawford, Nicholas Mantis, Laurel Finster, Noah M Merin, Akil Merchant, Karen L Reckamp, Gil Y Melmed, Jonathan Braun, Dermot McGovern, Samir Parekh, Douglas A Corley, Namvar Zohoori, Benjamin C Amick, Ruofei Du, Peter K Gregersen, Betty Diamond, Emanuela Taioli, Carlos Sariol, Ana Espino, Daniela Weiskopf, Alba Gifoni, James Brien, William Hanege, Marc Lipsitch, David A Zidar, Ann Scheck McAlearney, Ania Wajnberg, Joshua LaBaer, E Yvonne Lewis, Raquel A Binder, Ann M Moormann, Catherine Forconi, Sarah Forrester, Jennifer Batista, John Schieffelin, Dongjoo Kim, Giulia Biancon, Jennifer VanOudenhove, Stephanie Halene, Rong Fan, Dan H Barouch, Galit Alter, Swetha Pinninti, Suresh B Boppana, Sunil K Pati, Misty Latting, Andrew H Karaba, John Roback, Rafick Sekaly, Andrew Neish, Ahnalee M Brincks, Douglas A Granger, Amy B Karger, Bharat Thyagarajan, Stefani N Thomas, Sabra L Klein, Andrea L Cox, Todd Lucas, Debra Furr-Holden, Kent Key, Nicole Jones, Jens Wrammerr, Mehul Suthar, Serre Yu Wong, Natalie M Bowman, Viviana Simon, Lynne D Richardson, Russell McBride, Florian Krammer, Meenakshi Rana, Joshua Kennedy, Karl Boehme, Craig Forrest, Steve W Granger, Christopher D Heaney, Maria Knight Lapinski, Shannon Wallet, Ralph S Baric, Luca Schifanella, Marcos Lopez, Soledad Fernández, Eben Kenah, Ashish R Panchal, William J Britt, Iñaki Sanz, Madhav Dhodapkar, Rafi Ahmed, Luther A Bartelt, Alena J Markmann, Jessica T Lin, Robert S Hagan, Matthew C Wolfgang, Jacek Skarbinski

**Affiliations:** 1 Department of Medicine, Samuel Oschin Comprehensive Cancer Institute, Cedars-Sinai Medical Center, Los Angeles, CA, USA; 2 Department of Medicine, Hematology and Medical Oncology, Icahn School of Medicine at Mount Sinai, New York, NY, USA; 3 Division of Research, Kaiser Permanente Northern California, Oakland, CA, USA; 4 Fay W. Boozman College of Public Health, University of Arkansas for Medical Sciences, Little Rock, AR, USA; 5 Feinstein Institutes for Medical Research, Northwell Health, Manhasset, NY, USA; 6 Division of Infectious Diseases Wadsworth Center, New York State Department of Health, New York, NY, USA; 7 Division of Hematology and Cellular Therapy, Samuel Oschin Comprehensive Cancer Institute, Cedars-Sinai Medical Center, Los Angeles, CA, USA; 8 F. Widjaja Foundation Inflammatory Bowel and Immunobiology Research Institute, Los Angeles, CA, USA; 9 Unit of Comparative Medicine, University of Puerto Rico, Medical Sciences, San Juan, PR; 10 La Jolla Institute of Immunology, La Jolla CA, USA; 11 Department of Molecular Microbiology & Immunology, Saint Louis University, St. Louis MI, USA; 12 Center for Communicable Disease Dynamics, Department of Epidemiology, Harvard TH Chan School of Public Health, Bethesda, MD, USA; 13 Department of Medicine, Case Western Reserve University School of Medicine, Cleveland, OH, USA; 14 Department of Family and Community Medicine, Ohio State University College of Medicine, Columbus, OH, USA; 15 Department of Medicine, Icahn School of Medicine at Mount Sinai, New York, NY, USA; 16 Biodesign Virginia G. Piper Center for Personalized Diagnostics, Arizona State University, Tempe AZ, USA; 17 Department of Public Health, Michigan State University, Flint, MI, USA; 18 Department of Medicine, University of Massachusetts Chan Medical School, Worcester, MA, USA; 19 Department of Population and Quantitative Health Sciences, University of Massachusetts Chan Medical School, Worcester, MA, USA; 20 Department of Pediatrics, Tulane University School of Medicine, New Orleans, LA, USA; 21 Department of Biomedical Engineering, Yale University, New Haven, CT, USA; 22 Section of Hematology, Department of Internal Medicine and Yale Cancer Center, Yale University School of Medicine, New Haven, CT, USA; 23 Yale Cancer Center, New Haven, CT, USA; 24 The Center for Virology and Vaccine Research, Beth Israel Deaconess Medical Center, Boston, MA, USA; 25 Ragon Institute, Massachusetts Institute of Technology, Cambridge, MA, USA; 26 Department of Pediatrics, Heersink School of Medicine, University of Alabama at Birmingham, Birmingham, AL, USA; 27 Department of Medicine, Division of Infectious Diseases, Johns Hopkins University, Baltimore, MD, USA; 28 Department of Pathology and Laboratory Medicine, Emory University School of Medicine, Atlanta, GA, USA; 29 Department of Human Development and Family Studies, College of Social Science, Michigan State University, East Lansing, MI, USA; 30 Institute for Interdisciplinary Salivary Bioscience Research, University of California at Irvine; Department of Pediatrics, Johns Hopkins University School of Medicine, Baltimore, MD, USA; 31 Department of Laboratory Medicine and Pathology, University of Minnesota, Minneapolis, MN, USA; 32 W. Harry Feinstone Department of Molecular Microbiology and Immunology, The Johns Hopkins Bloomberg School of Public Health, Baltimore, Maryland, USA; 33 Division of Public Health, College of Human Medicine, Michigan State University, East Lansing, MI, USA; 34 Department of Pediatrics, Division of Infectious Disease, Emory University, Atlanta, GA, USA; 35 The Henry D. Janowitz Division of Gastroenterology, Department of Medicine, Icahn School of Medicine at Mount Sinai, New York, NY, USA; 36 University of North Carolina School of Medicine, Division of Infectious Diseases, Chapel Hill, NC, USA; 37 Department of Microbiology, Icahn School of Medicine at Mount Sinai, New York, NY, USA; 38 Institute for Health Equity Research and Department of Emergency Medicine, Icahn School of Medicine at Mount Sinai, New York, NY, USA; 39 Department of Pathology, Icahn School of Medicine at Mount Sinai, New York, NY, USA; 40 Department of Pediatrics, College of Medicine, University of Arkansas for Medical Sciences, Little Rock, AR, USA; 41 Department of Microbiology and Immunology, College of Medicine, University of Arkansas for Medical Sciences, Little Rock, AR, USA; 42 Salimetrics, LLC, Carlsbad, CA, USA; 43 Department of Environmental Health and Engineering, Bloomberg School of Public Health, Johns Hopkins University, Baltimore, Maryland, USA; 44 Department of Communication, Michigan AgBio Research, Michigan State University, East Lansing, MI, USA; 45 School of Dentistry, Department of Oral and Craniofacial Health Sciences, University of North Carolina School of Medicine, Chapel Hill, NC, USA; 46 Gillings School of Global Public Health, Department of Epidemiology, University of North Carolina School of Medicine, Chapel Hill, NC, USA; 47 Division of Surgical Outcomes and Precision Medicine Research, Department of Surgery, University of Minnesota, Minneapolis, MN, USA; 48 Puerto Rico Public Health Trust, Puerto Rico Science, Technology and Research Trust and University of Puerto Rico at Humacao, Medical Sciences, San Juan, PR, USA; 49 Department of Biomedical Informatics, Center for Biostatistics, Ohio State University College of Medicine, Columbus, OH, USA; 50 Division of Biostatistics, College of Public Health, The Ohio State University, Columbus, OH, USA; 51 Department of Emergency Medicine, The Ohio State University Wexner Medical Center, Columbus, OH, USA; 52 Department of Immunology, Heersink School of Medicine, University of Alabama at Birmingham, Birmingham, AL, USA; 53 Department of Medicine, Emory University School of Medicine, Atlanta, GA, USA; 54 Department of Microbiology and Immunology, Emory University School of Medicine, Atlanta, GA, USA; 55 Department of Medicine, Division of Infectious Diseases, University of North Carolina School of Medicine, Chapel Hill, NC, USA; 56 Marsico Lung Institute and Department of Microbiology and Immunology, University of North Carolina School of Medicine, Chapel Hill, NC, USA

**Keywords:** COVID-19, SARS-CoV-2, epidemiology, cohort, serosurveillance, SeroNet

## Abstract

Global efforts are needed to elucidate the epidemiology of Severe Acute Respiratory Syndrome Coronavirus 2 (SARS-CoV-2), the underlying cause of coronavirus disease 2019 (COVID-19) including seroprevalence, risk factors and long-term sequelae, as well as immune responses following vaccination across populations and the social dimensions of prevention and treatment strategies. In the U.S., the National Cancer Institute in partnership with the National Institute of Allergy and Infectious Diseases, established the SARS-CoV-2 Serological Sciences Network (SeroNet) as the nation’s largest coordinated effort to study COVID-19. The network is comprised of multidisciplinary researchers bridging gaps and fostering collaborations between immunologists, epidemiologists, virologists, clinicians and clinical laboratories, social and behavioral scientists, policy makers, data scientists, and community members. In total, 49 institutions form the SeroNet consortium to study individuals with cancer, autoimmune disease, inflammatory bowel diseases, cardiovascular diseases, HIV, transplant recipients, as well as otherwise healthy pregnant women, children, college students, and high-risk occupational workers (including health care workers and first responders). Several studies focus on underrepresented populations, including ethnic minorities and rural communities. To support integrative data analyses across SeroNet studies, efforts are underway to define common data elements for standardized serology measurements, cellular and molecular assays, self-reported data, treatment, and clinical outcomes. In this paper, we discuss the overarching framework for SeroNet epidemiology studies, critical research questions under investigation, and data accessibility for the worldwide scientific community. Lessons learned will help inform preparedness and responsiveness to future emerging diseases.

## Supplementary Material

ofac171_suppl_Supplementary_Appendix_A

ofac171_suppl_Supplementary_Appendix_B

