# Mission, Organization, and Future Direction of the Serological Sciences Network for COVID-19 (SeroNet) Epidemiologic Cohort Studies

**DOI:** 10.1093/ofid/ofac171

**Published:** 2022-04-27

**Authors:** Jane C Figueiredo, Fred R Hirsch, Lawrence H Kushi, Wendy N Nembhard, James M Crawford, Nicholas Mantis, Laurel Finster, Noah M Merin, Akil Merchant, Karen L Reckamp, Gil Y Melmed, Jonathan Braun, Dermot McGovern, Samir Parekh, Douglas A Corley, Namvar Zohoori, Benjamin C Amick, Ruofei Du, Peter K Gregersen, Betty Diamond, Emanuela Taioli, Carlos Sariol, Ana Espino, Daniela Weiskopf, Alba Gifoni, James Brien, William Hanege, Marc Lipsitch, David A Zidar, Ann Scheck McAlearney, Ania Wajnberg, Joshua LaBaer, E Yvonne Lewis, Raquel A Binder, Ann M Moormann, Catherine Forconi, Sarah Forrester, Jennifer Batista, John Schieffelin, Dongjoo Kim, Giulia Biancon, Jennifer VanOudenhove, Stephanie Halene, Rong Fan, Dan H Barouch, Galit Alter, Swetha Pinninti, Suresh B Boppana, Sunil K Pati, Misty Latting, Andrew H Karaba, John Roback, Rafick Sekaly, Andrew Neish, Ahnalee M Brincks, Douglas A Granger, Amy B Karger, Bharat Thyagarajan, Stefani N Thomas, Sabra L Klein, Andrea L Cox, Todd Lucas, Debra Furr-Holden, Kent Key, Nicole Jones, Jens Wrammerr, Mehul Suthar, Serre Yu Wong, Natalie M Bowman, Viviana Simon, Lynne D Richardson, Russell McBride, Florian Krammer, Meenakshi Rana, Joshua Kennedy, Karl Boehme, Craig Forrest, Steve W Granger, Christopher D Heaney, Maria Knight Lapinski, Shannon Wallet, Ralph S Baric, Luca Schifanella, Marcos Lopez, Soledad Fernández, Eben Kenah, Ashish R Panchal, William J Britt, Iñaki Sanz, Madhav Dhodapkar, Rafi Ahmed, Luther A Bartelt, Alena J Markmann, Jessica T Lin, Robert S Hagan, Matthew C Wolfgang, Jacek Skarbinski

**Affiliations:** Department of Medicine, Samuel Oschin Comprehensive Cancer Institute, Cedars-Sinai Medical Center, Los Angeles, California, USA; Department of Medicine, Hematology and Medical Oncology, Icahn School of Medicine at Mount Sinai, New York, New York, USA; Division of Research, Kaiser Permanente Northern California, Oakland, California, USA; Fay W. Boozman College of Public Health, University of Arkansas for Medical Sciences, Little Rock, Arkansas, USA; Feinstein Institutes for Medical Research, Northwell Health, Manhasset, New York, USA; Division of Infectious Diseases Wadsworth Center, New York State Department of Health, New York, New York, USA; Department of Medicine, Samuel Oschin Comprehensive Cancer Institute, Cedars-Sinai Medical Center, Los Angeles, California, USA; Division of Hematology and Cellular Therapy, Samuel Oschin Comprehensive Cancer Institute, Cedars-Sinai Medical Center, Los Angeles, California, USA; Division of Hematology and Cellular Therapy, Samuel Oschin Comprehensive Cancer Institute, Cedars-Sinai Medical Center, Los Angeles, California, USA; Department of Medicine, Samuel Oschin Comprehensive Cancer Institute, Cedars-Sinai Medical Center, Los Angeles, California, USA; F. Widjaja Foundation Inflammatory Bowel and Immunobiology Research Institute, Cedars-Sinai Medical Center, Los Angeles, California, USA; F. Widjaja Foundation Inflammatory Bowel and Immunobiology Research Institute, Cedars-Sinai Medical Center, Los Angeles, California, USA; F. Widjaja Foundation Inflammatory Bowel and Immunobiology Research Institute, Cedars-Sinai Medical Center, Los Angeles, California, USA; Department of Medicine, Hematology and Medical Oncology, Icahn School of Medicine at Mount Sinai, New York, New York, USA; Division of Research, Kaiser Permanente Northern California, Oakland, California, USA; Fay W. Boozman College of Public Health, University of Arkansas for Medical Sciences, Little Rock, Arkansas, USA; Fay W. Boozman College of Public Health, University of Arkansas for Medical Sciences, Little Rock, Arkansas, USA; Winthrop Rockefeller Cancer Institute, University of Arkansas for Medical Sciences, Little Rock, Arkansas, USA; Fay W. Boozman College of Public Health, University of Arkansas for Medical Sciences, Little Rock, Arkansas, USA; Feinstein Institutes for Medical Research, Northwell Health, Manhasset, New York, USA; Feinstein Institutes for Medical Research, Northwell Health, Manhasset, New York, USA; Department of Medicine, Hematology and Medical Oncology, Icahn School of Medicine at Mount Sinai, New York, New York, USA; Unit of Comparative Medicine, University of Puerto Rico, Medical Sciences, San Juan, Puerto Rico, USA; Unit of Comparative Medicine, University of Puerto Rico, Medical Sciences, San Juan, Puerto Rico, USA; La Jolla Institute of Immunology, La Jolla, California, USA; La Jolla Institute of Immunology, La Jolla, California, USA; Department of Molecular Microbiology and Immunology, Saint Louis University, St. Louis, Missouri, USA; Center for Communicable Disease Dynamics, Department of Epidemiology, Harvard TH Chan School of Public Health, Bethesda, Maryland, USA; Center for Communicable Disease Dynamics, Department of Epidemiology, Harvard TH Chan School of Public Health, Bethesda, Maryland, USA; Department of Medicine, Case Western Reserve University School of Medicine, Cleveland, Ohio, USA; Department of Family and Community Medicine, Ohio State University College of Medicine, Columbus, Ohio, USA; Department of Medicine, Icahn School of Medicine at Mount Sinai, New York, New York, USA; Biodesign Virginia G. Piper Center for Personalized Diagnostics, Arizona State University, Tempe, Arizona, USA; Department of Public Health, Michigan State University, Flint, Michigan, USA; Department of Medicine, University of Massachusetts Chan Medical School, Worcester, Massachusetts, USA; Department of Medicine, University of Massachusetts Chan Medical School, Worcester, Massachusetts, USA; Department of Medicine, University of Massachusetts Chan Medical School, Worcester, Massachusetts, USA; Department of Population and Quantitative Health Sciences, University of Massachusetts Chan Medical School, Worcester, Massachusetts, USA; Department of Population and Quantitative Health Sciences, University of Massachusetts Chan Medical School, Worcester, Massachusetts, USA; Department of Pediatrics, Tulane University School of Medicine, New Orleans, Louisiana, USA; Department of Biomedical Engineering, Yale University, New Haven, Connecticut, USA; Section of Hematology, Department of Internal Medicine, Yale University School of Medicine, New Haven, Connecticut, USA; Section of Hematology, Department of Internal Medicine, Yale University School of Medicine, New Haven, Connecticut, USA; Section of Hematology, Department of Internal Medicine, Yale University School of Medicine, New Haven, Connecticut, USA; Yale Cancer Center, New Haven, Connecticut, USA; Department of Biomedical Engineering, Yale University, New Haven, Connecticut, USA; Yale Cancer Center, New Haven, Connecticut, USA; The Center for Virology and Vaccine Research, Beth Israel Deaconess Medical Center, Boston, Massachusetts, USA; Ragon Institute, Massachusetts Institute of Technology, Cambridge, Massachusetts, USA; Departments of Pediatrics, Heersink School of Medicine, University of Alabama at Birmingham, Birmingham, Alabama, USA; Departments of Pediatrics, Heersink School of Medicine, University of Alabama at Birmingham, Birmingham, Alabama, USA; Departments of Pediatrics, Heersink School of Medicine, University of Alabama at Birmingham, Birmingham, Alabama, USA; Departments of Pediatrics, Heersink School of Medicine, University of Alabama at Birmingham, Birmingham, Alabama, USA; Department of Medicine, Division of Infectious Diseases, Johns Hopkins University, Baltimore, Maryland, USA; Department of Pathology and Laboratory Medicine, Emory University School of Medicine, Atlanta, Georgia, USA; Department of Pathology and Laboratory Medicine, Emory University School of Medicine, Atlanta, Georgia, USA; Department of Pathology and Laboratory Medicine, Emory University School of Medicine, Atlanta, Georgia, USA; Department of Human Development and Family Studies, College of Social Science, Michigan State University, East Lansing, Michigan, USA; Institute for Interdisciplinary Salivary Bioscience Research, University of California at Irvine, Irvine, California, USA; Department of Pediatrics, Johns Hopkins University School of Medicine, Baltimore, Maryland, USA; Department of Laboratory Medicine and Pathology, University of Minnesota, Minneapolis, Minnesota, USA; W. Harry Feinstone Department of Molecular Microbiology and Immunology, The Johns Hopkins Bloomberg School of Public Health, Baltimore, Maryland, USA; Department of Laboratory Medicine and Pathology, University of Minnesota, Minneapolis, Minnesota, USA; W. Harry Feinstone Department of Molecular Microbiology and Immunology, The Johns Hopkins Bloomberg School of Public Health, Baltimore, Maryland, USA; Department of Medicine, Division of Infectious Diseases, Johns Hopkins University, Baltimore, Maryland, USA; W. Harry Feinstone Department of Molecular Microbiology and Immunology, The Johns Hopkins Bloomberg School of Public Health, Baltimore, Maryland, USA; Division of Public Health, College of Human Medicine, Michigan State University, East Lansing, Michigan, USA; Division of Public Health, College of Human Medicine, Michigan State University, East Lansing, Michigan, USA; Division of Public Health, College of Human Medicine, Michigan State University, East Lansing, Michigan, USA; Division of Public Health, College of Human Medicine, Michigan State University, East Lansing, Michigan, USA; Department of Pediatrics, Division of Infectious Disease, Emory University, Atlanta, Georgia, USA; Department of Pediatrics, Division of Infectious Disease, Emory University, Atlanta, Georgia, USA; The Henry D. Janowitz Division of Gastroenterology, Department of Medicine, Icahn School of Medicine at Mount Sinai, New York, New York, USA; Department of Medicine, Division of Infectious Diseases, University of North Carolina School of Medicine, Chapel Hill, North Carolina, USA; Department of Microbiology, Icahn School of Medicine at Mount Sinai, New York, New York, USA; Institute for Health Equity Research and Department of Emergency Medicine, Icahn School of Medicine at Mount Sinai, New York, New York, USA; Department of Pathology, Icahn School of Medicine at Mount Sinai, New York, New York, USA; Department of Microbiology, Icahn School of Medicine at Mount Sinai, New York, New York, USA; Department of Pathology, Icahn School of Medicine at Mount Sinai, New York, New York, USA; Department of Pathology, Icahn School of Medicine at Mount Sinai, New York, New York, USA; Department of Pediatrics, College of Medicine, University of Arkansas for Medical Sciences, Little Rock, Arkansas, USA; Department of Microbiology and Immunology, College of Medicine, University of Arkansas for Medical Sciences, Little Rock, Arkansas, USA; Department of Microbiology and Immunology, College of Medicine, University of Arkansas for Medical Sciences, Little Rock, Arkansas, USA; Salimetrics, LLC, Carlsbad, California, USA; Department of Environmental Health and Engineering, Bloomberg School of Public Health, Johns Hopkins University, Baltimore, Maryland, USA; Department of Communication, Michigan AgBio Research, Michigan State University, East Lansing, Michigan, USA; Department of Oral and Craniofacial Health Sciences, School of Dentistry, University of North Carolina School of Medicine, Chapel Hill, North Carolina, USA; Department of Epidemiology, Gillings School of Global Public Health, University of North Carolina School of Medicine, Chapel Hill, North Carolina, USA; Division of Surgical Outcomes and Precision Medicine Research, Department of Surgery, University of Minnesota, Minneapolis, Minnesota, USA; Puerto Rico Public Health Trust, Puerto Rico Science, Technology and Research Trust and University of Puerto Rico at Humacao, Medical Sciences, San Juan, Puerto Rico, USA; Department of Biomedical Informatics, Center for Biostatistics, Ohio State University College of Medicine, Columbus, Ohio, USA; Division of Biostatistics, College of Public Health, The Ohio State University, Columbus, Ohio, USA; Department of Emergency Medicine, The Ohio State University Wexner Medical Center, Columbus, Ohio, USA; Department of Immunology, Heersink School of Medicine, University of Alabama at Birmingham, Birmingham, Alabama, USA; Department of Medicine, Emory University School of Medicine, Atlanta, Georgia, USA; Department of Medicine, Emory University School of Medicine, Atlanta, Georgia, USA; Department of Microbiology and Immunology, Emory University School of Medicine, Atlanta, Georgia, USA; Department of Medicine, Division of Infectious Diseases, University of North Carolina School of Medicine, Chapel Hill, North Carolina, USA; Department of Medicine, Division of Infectious Diseases, University of North Carolina School of Medicine, Chapel Hill, North Carolina, USA; Department of Medicine, Division of Infectious Diseases, University of North Carolina School of Medicine, Chapel Hill, North Carolina, USA; Department of Medicine, Division of Infectious Diseases, University of North Carolina School of Medicine, Chapel Hill, North Carolina, USA; Marsico Lung Institute and Department of Microbiology and Immunology, University of North Carolina School of Medicine, Chapel Hill, North Carolina, USA; Division of Research, Kaiser Permanente Northern California, Oakland, California, USA

**Keywords:** cohort, COVID-19, epidemiology, SARS-CoV-2, serosurveillance, SeroNet

## Abstract

**Background:**

Global efforts are needed to elucidate the epidemiology of severe acute respiratory syndrome coronavirus 2 (SARS-CoV-2), the underlying cause of coronavirus disease 2019 (COVID-19), including seroprevalence, risk factors, and long-term sequelae, as well as immune responses after vaccination across populations and the social dimensions of prevention and treatment strategies.

**Methods:**

In the United States, the National Cancer Institute in partnership with the National Institute of Allergy and Infectious Diseases, established the SARS-CoV-2 Serological Sciences Network (SeroNet) as the nation’s largest coordinated effort to study coronavirus disease 2019. The network comprises multidisciplinary researchers bridging gaps and fostering collaborations among immunologists, epidemiologists, virologists, clinicians and clinical laboratories, social and behavioral scientists, policymakers, data scientists, and community members. In total, 49 institutions form the SeroNet consortium to study individuals with cancer, autoimmune disease, inflammatory bowel diseases, cardiovascular diseases, human immunodeficiency virus, transplant recipients, as well as otherwise healthy pregnant women, children, college students, and high-risk occupational workers (including healthcare workers and first responders).

**Results:**

Several studies focus on underrepresented populations, including ethnic minorities and rural communities. To support integrative data analyses across SeroNet studies, efforts are underway to define common data elements for standardized serology measurements, cellular and molecular assays, self-reported data, treatment, and clinical outcomes.

**Conclusions:**

In this paper, we discuss the overarching framework for SeroNet epidemiology studies, critical research questions under investigation, and data accessibility for the worldwide scientific community. Lessons learned will help inform preparedness and responsiveness to future emerging diseases.

## Teaser

The SARS-CoV-2 Serological Sciences Network (SeroNet) is a large US multi-institutional coordinated initiative that researches immune response to SARS-CoV-2 infection and vaccination. The SeroNet research program and infrastructure development can help inform preparedness and response for other emerging diseases worldwide.

Coronavirus disease 2019 (COVID-19), an illness caused by infection with the severe acute respiratory coronavirus 2 (SARS-CoV-2), was first detected in December 2019 and designated a worldwide pandemic by the World Health Organization (WHO) on March 11, 2020 [1]. By April 2020, the danger that COVID-19 could overwhelm healthcare systems was apparent after propagated outbreaks throughout the world. Governments and public health agencies in many countries struggled to implement public health initiatives such as physical distancing, mask/face coverings, and, in some cases, stay-at-home orders in attempts to curb the number of infections or “flatten the curve”. Global efforts were launched to elucidate the epidemiology of this new disease, including its seroprevalence, risk factors, individual susceptibility, and long-term sequelae, in addition to developing effective therapeutics and vaccines.

In response to the COVID-19 pandemic, the US National Cancer Institute (NCI), in partnership with the National Institute of Allergy and Infectious Diseases (NIAID), Frederick National Laboratory for Cancer Research (FNLCR) and other parts of the National Institutes of Health (NIH), and the Department of Health and Human Services, established the Serological Sciences Network (SeroNet) as the nation’s largest coordinated effort to study the human immune response to COVID-19 through a Congressional emergency appropriation of funding [2]. The overall goal of SeroNet is (1) to expand the nation’s capacity for accessible and efficient SARS-CoV-2 serologic tests on a population-level and (2) to advance research on humoral and cellular immune responses to SARS-CoV-2 infection and vaccination among diverse and vulnerable populations. Another key objective is developing culturally targeted communication approaches to promote SARS-CoV-2 antibody testing and to better understand barriers that influence knowledge of and participation among minority communities in testing with the goal to address overall racial/ethnic disparities in COVID-19 susceptibility and outcomes. Lessons learned from SeroNet research can be applied immediately and may prove valuable both to (1) the development of vaccines and novel treatments and, (2) to inform future public health emergencies.

In this report, we discuss the overarching framework for the performance of SeroNet studies, and target outcomes of the consortium. By providing this foundational information, we alert the global scientific and medical community about data emerging from SeroNet studies to help drive the global response to the COVID-19 pandemic.

## METHODS

### Members of SeroNet

**Figure 1.**
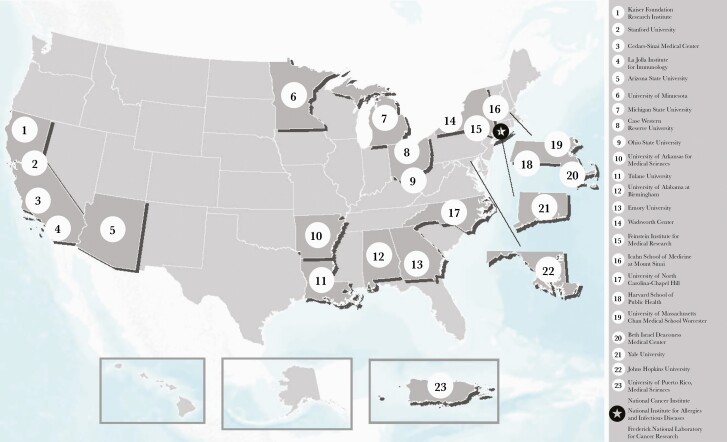
Primary US Institutions Participating in SeroNet Studies.

The NCI established 8 Serological Sciences Centers of Excellence to conduct research projects to characterize immune responses to SARS-CoV-2 infection and better understand predictors of protective immune responses and disease progression (Figure 1). In addition, 13 awards were granted to researchers to conduct projects on basic and applied serological research. Through the FNLCR, 4 subcontracts were awarded to research institutions as SeroNet Capacity Building Centers to expand the nation’s serology testing capabilities by increasing throughput, developing novel serological assays to test for SARS-CoV-2 antibodies, procuring reference serological samples, and conducting serosurveillance studies. In total, SeroNet granted 25 awards to 23 of the nation’s top biomedical research institutions.

### Evolving Research Questions

Upon establishment of SeroNet in October 2020 and naming the 25 awardees spread across 49 institutions (23 primary institutions, 26 additional collaborating institutions) overarching research questions were articulated. They focused on understanding susceptibility and diversity of exposures to SARS-CoV-2 including elucidating immune responses to infection in the general population and among high-risk and immunocompromised populations.

By December 2020, Pfizer-BioNTech [3] and Moderna/NIAID [4] had begun clinical trials in healthy populations on their respective mRNA SARS-CoV-2 vaccines, and they reported >94% short-term vaccine efficacy against hospitalization and mortality, with no evidence of increased incidence of major adverse events. These findings led to Emergency Use Authorization of the vaccines in Britain, the United States, the European Union, and several other countries. Soon thereafter, several other COVID-19 vaccines were approved by the WHO for use globally, including Johnson & Johnson/Janssen INJ-7843735/Ad26.COV2.S, Oxford/AstraZeneca AZD1222, Serum Institute of India Covishield (Oxford/AstraZeneca formulation), Sinopharm (Beijing) BBIBP-CorV (Vero Cells), and Sinovac CoronaVac [1, 5]. With over 20 SARS-CoV-2 vaccines now available around the globe [6], the SeroNet research infrastructure also supports questions focused on vaccine responses, including the durability of humoral and cellular immunity in immunocompromised populations compared with healthy individuals, and the frequency of breakthrough infections in vaccinated individuals.

In the coming months, the COVID-19 pandemic will no doubt continue to rapidly change both biologically, with emergence of new variants, and medically, with development of new vaccines and variations in vaccine perspectives, availability, and uptake across populations, and antiviral agents. Recommendations for “booster” (ie, subsequent dose vaccine administrations) and novel treatments for symptomatic disease are already changing the landscape. Public health policy will also evolve, with full US Food and Drug Administration authorization of vaccines and likely vaccine mandates by employers and communities. As such, SeroNet studies will continue to be refined to address research challenges that arise, including devising strategies related to vaccination uptake among hesitant and underserved populations (Table 1).

**Table 1.** Evolution of Scientific Inquiry in SeroNet

Research Questions in Prevaccine Era	Research Questions in Postvaccine Era	Future Directions
What is the prevalence of SARS-CoV-2 infection in the United States across age groups, racial/ethnic groups, and urban/rural populations?	Do persons with immunosuppression develop similar immune responses after SARS-CoV-2 vaccination as healthy individuals?	What are the trajectories of immune response after natural infection and/or vaccination? Is the pandemic over for otherwise healthy populations?
Why do some people who are exposed to SARS-CoV-2 develop symptoms and others do not?	Do specific immune suppressive therapies affect risk of SARS-CoV-2 infection or vaccination response (eg, cancer therapies including immunotherapies)?	Do additional doses/boosters of vaccine among immunosuppressed persons provide increased protection from infection or severe COVID-19?
What risk factors explain the spectrum of disease severity among those diagnosed with COVID-19? How do we define “long COVID” (postacute sequelae of SARS-CoV-2, PASC) and what are the predictors?	What is the durability of the vaccine-induced immune response across diverse populations? What T-cell responses to SARS-CoV-2 occur following infection and/or vaccination?	What is the optimal timing of vaccination relative to treatment for disease management? How does heterologous vaccination differ from homologous vaccination?
What are the risk factors associated with reinfection?	What is the clinical significance of “breakthrough COVID” in vaccinated populations?	Is serological measures of antibodies useful as a means to monitor vulnerable individuals and/or help guide vaccination policy?
How does disease severity correlate with long-term immunity to reinfection?	What are the characteristics of “low” vaccine responders? What alternative strategies are needed to protect them?	How do booster vaccines hold up against future “variants of concern”?
What genetic, clinical, and environmental factors affect the immune response to SARS-CoV-2?	Does vaccination decrease the likelihood of risk of severe illness? Long COVID?	What are barriers or enhancers of vaccine uptake among minority populations (eg, black and Hispanic communities), across the lifespan?
Do people with certain health conditions, such as cancer, diabetes, heart disease, or autoimmune disease, have an increased risk of developing severe illness from COVID-19?	What is the level of vaccine hesitancy across various populations? How do we address concerns?	What tools and resources are needed to enable broad and effective, home-based salivary collection?
Do culturally targeted messages about COVID-19 and noninvasive salivary antibody testing increase participation in research uptake among minority populations (eg, black and Hispanic communities)?	Do culturally targeted messages about COVID-19 and vaccines increase vaccination rates uptake among minority populations (eg, black and Hispanic communities)?	What are the interactions between anti-SARS-CoV-2 monoclonal antibody therapy for treatment and prevention of SARS-CoV-2 and development and maintenance of an immune response?
What is the significance of SARS-CoV-2 “variants of concern”?	What role can salivary antibody testing play in addressing vaccination hesitancy and booster vaccination among hesitant and underserved populations?	How do we prepare for the next pandemic?

Abbreviations: COVID-19, coronavirus disease 2019; SARS-CoV-2, severe acute respiratory syndrome coronavirus 2.

### RESULTS

#### Scope of Epidemiologic Research

There is a broad range of research studies across SeroNet, including epidemiologic studies, basic investigational science, development and deployment of serologic diagnostic methods, mathematical and statistical modeling, and qualitative research including focus groups, online surveys, and focus groups/qualitative interviews. Working groups were established to outline study design templates (Appendix A) to disseminate best practices within the network and broader community and to allow for future data harmonization. Among epidemiologic studies, the majority are prospective cohort studies with repeated measures focusing on various research questions across diverse populations, with strategic, real-world observational studies also included. Specific details on study aims and methodology for each SeroNet study involving human populations are outlined in Table 2 and Appendix B. The following subpopulations are being examined in SeroNet studies.

**Table 2.** Description of Epidemiologic Studies in SeroNet

Institution/Award	Project Title	Study Design	Study Population	Proposed Sample Size	Methods	Biospecimens and Assays
Arizona State University, CBC21X089	Multiplexed In-solution Serological Test for SARS-CoV-2, Human Coronaviruses, and Other Respiratory Pathogens	Prospective cohort study	□ HIV, cancer, and transplant patients and immunocompetent controls; □ All ages, M/F; □ Any race/ethnicity; □ Arizona/New York	1125 immunocompromised; 375 controls; 500 postnatural infection	3/2021–10/2025; Samples collected prevaccination and then 1, 3, 6, 12, 24 months postvaccination; Survey, medical records	Serum, PBMC, anterior nasal swab and saliva; MISPA to assay antibodies against the immunodominant antigens from SARS-CoV-2, other 6 human coronaviruses, and additional respiratory pathogens
Case Western Reserve School of Medicine and The Lerner School of Medicine, U01CA260513	Pre-exposure Immunologic Health and Linkages to SARS-COV2 Serologic Responses, Endothelial Cell Resilience, and Cardiovascular Complications: Defining the Mechanistic Basis of High Risk Endotypes (Cardio-COVID)	Retrospective cohort study	□ US veterans with COVID-19 receiving care within the Veterans Administration Health System; □ >18 yo, M/F; □ Any race/ethnicity; □ United States	150 000	3/2019–12/2021; Medical records	Serum; Reactivity to the full-length S protein, the receptor binding domain (RBD) of the S1 protein and N protein,
Case Western Reserve University, U01CA260539	Early Drivers of Humoral Immunity to SARS-CoV-2 Infections	Prospective cohort study	□ Individuals exposed to people known to have COVID-19; □ >12 yo, M/F; □ Any race/ethnicity; □ Northeast Ohio	200	9/2021–present; Peripheral blood along with nasopharyngeal swabs, and saliva sampled on days 0, 1, 3, 7, 10, 14, and 28 and every 6 months for up to 3 years	Serum, Saliva and PBMC; Bead array assays to measure antibodies to S and N proteins and RBD in serum, S and N proteins and RBD- specific sIgA and IgG in saliva relative to total IgA and IgG in saliva, Ag-specific B cells in peripheral circulation (flow cytometry and ELISPOT) and isolating individual Ag-specific B cells to determine somatic mutations
^a^Cedars-Sinai Medical Center, U54CA260591	Diversity and Determinants of the Immune-Inflammatory Response to SARS-CoV-19	Prospective cohort study	□ Healthcare workers (including those recovering COVID-19 patients and their household contacts), cancer patients, patients with IBD; □ >18 yo, M/F; □ Any race/ethnicity; □ Los Angeles and surrounding areas	2060 health workers, 1000 cancer patients, 175 IBD patients	12/2020–9/2025; Samples collected pre- and postvaccination up to 5 years; self-completed questionnaires, medical records	Plasma, Buffy, PBMC; Antibody levels using Abbott assays for RBD and N protein; T cell repertoire using Adaptive; proteomics; metabolomics
College of Human Medicine, Michigan State University, U01CA260469	Culturally Targeted Communication to Promote SARS-CoV-2 Antibody Testing in Saliva: Enabling Evaluation of Inflammatory Pathways in COVID-19 Racial Disparities	Randomized control trial	□ Black and white members of Flint Registry; □ >18yo, M/F; □ Black/African American and White; □ Flint, Michigan	500	6/2021–6/2024, Baseline measures, Surveys	Saliva; Multiplex salivary antibody assay for anti-nucleocapsid, RBD. IgG, IgA, and IgM. Panel of inflammatory markers including IFN-γ, TNF-α, IL-1β, IL-2, IL-5, IL-6, IL-7, IL-8, IL-10, IL-12p70, IL-13, IL-17A
^a^Emory University, U54CA260563	Immune Regulation of COVID-19 Infection in Cancer and Autoimmunity	Prospective cohort study	□ Hospital inpatients newly admitted due to a positive SARS-CoV-2 RT-PCR test. Emphasis on patients with cancer, obesity, immune compromise, and other conditions that could affect the viral immune response. □ >18 yo, M/F; □ Any race/ethnicity; □ Atlanta and surrounding communities	93 to date; plan to continue enrollment during each viral wave in Atlanta	4/2021–present; Samples collected upon admission to hospital with positive test for SARS-CoV-2, at discharge, and then 3 and 6 months after discharge; survey, medical records	Serum, NGS of NP swab sample, flow cytometry, ELISPOT, viral neutralization, RNA-Seq, multiplex cytokine assays, metabolomics
Feinstein Institutes for Medical Research, Northwell Health, CBC21X090	Serological Sciences Network Capacity Building Center	Prospective cohort study	□ Autoimmune Conditions (Systemic Lupus Erythematosus; Sjögren’s syndrome, rheumatoid arthritis) and immunocompetent controls; □ >18 yo, M/F; □ Any race/ethnicity; □ New York City metropolitan area	700 controls and 400 with autoimmune disease	6/2021– 12/2021; Time 0, 2 months, 6 months, 12 months, 24 months; Medical records	SARS-CoV-2 antibody assays on serum or plasma: Roche Cobas Elecsys, DiaSorin LIAISON
^a^Icahn School of Medicine at Mount Sinai, U54CA260560	Characterization of the Antibody Response to SARS-CoV-2 in Lung Cancer Patients	Prospective cohort study	□ Lung cancer patients and controls; □ >18 yo, M/F; □ Any race/ethnicity; □ New York City metropolitan area	2000	10/2020–4/2024; At time 0, 3, 6, 12, 24 months; Survey, medical records	SARS-CoV-2 antibody assays on serum
Icahn School of Medicine at Mount Sinai, CBC21X092	Serological Sciences Network Capacity Building Center	Prospective, longitudinal study	□ Persons with Inflammatory bowel disease □ Persons with multiple myeloma □ Solid organ transplant recipients; □ Healthy controls □ >18 yo, M/F; □ Any race/ethnicity; □ New York City metropolitan area	400	02/2021–01/2023; 6 visits: 1 prevaccine (if feasible), and longitudinally at 3, 6, 12, and 24 months	Serum, PBMC, Mount Sinai/Kantaro; Enzyme-linked immunosorbent assay (ELISA)
^a^Johns Hopkins University, U54CA260492-01	Johns Hopkins Excellence in Pathogenesis and Immunity Center for SARS-CoV-2 (JH-EPICS)	Prospective cohort study	□ HIV, cancer, and transplant patients and immunocompetent controls; □ All ages, M/F; □ Any race/ethnicity; □ Maryland	2000	Prevaccine, 2 weeks postvaccine, then every 6 months; Hospitalized participants: Diagnosis (Day 0), Day 1, Day 3, Day 7, Weekly, Day 28, Month 3, 6, 9, 12, 18, 24/Ambulatory Participants: Diagnosis (Day 0), Day 28, Month 3, 6, 9, 12, 18, 24	Serum, plasma, PBMC, and nasal and oropharyngeal swabs; Mesoscale Discovery Assay (MSD) and ELISA to assay antibodies and antibody subtypes directed against SARS-CoV-2 proteins, MSD assays for cytokines and chemokines, metabolic immune cell flow cytometry, virus neutralization assays, antibody-dependent cellular cytotoxicity, complement-mediated cytotoxicity, complement fixation, ViraFEST and ELISpot
Kaiser Permanente Northern California, U01CA260584	SARS-CoV-2 Serological Antibody Testing for Disease Surveillance and Clinical Use	Serial seroprevalence surveys with built in longitudinal follow-up of a subset of participants	□ Kaiser Permanente Northern California members aged ≥7 years old; □ >7yo, M/F; □ Any race/ethnicity; □ Northern and Central California	Seroprevalence: 3000 per month × 24 months = 72 000; Longitudinal follow-up group: 1200	4/2021–3/2023; At time 0 and 3-month for seroprevalence survey; At time 0, 3, 6, 12, 24 months for longitudinal follow-up subgroup; Survey, medical records	Serum; Serum/ Diasorin LIAISON SARS-CoV-2 S1/S2 IgG test AND Siemens SARS-CoV-2 Total Assay on ADVIA Centaur Platform
^a^Ohio State University, U54CA260582	Center for Serological Testing to Improve Outcomes from Pandemic COVID-19 (STOP-COVID)	Prospective cohort study	□ First responders, healthcare workers, and their household contacts; □ Any age, M/F □ Any race/ethnicity □ Central Ohio	2500	2/2021–8/2026; Time 0 and then every 180 days; Survey	Whole blood for serology; nasal swab for PCR. Saliva and biorepository specimens; anti-S (qual), anti-N (qual), trimeric anti-S (qual), unique S peptide alpha, unique N peptide alpha, unique S peptide beta, unique N peptide beta, unique S peptide SARS, unique N peptide SARS, common (cross-reactive) S peptide and N peptide, neutralizing titer(s) WT, D614G, B.1.1.7, B.1.351, P1, B.1.617.2,SARS, SARS QC coverage, SARS strain (Pango & GISAID), RSV A, RSV B, influenza A(H3N2), influenza A (H1N1), influenza B, human coronavirus HKU1, human coronavirus OC43, human coronavirus NL63, human coronavirus 229E, human metapneumovirus (HMPV), human adenovirus (HAdV), IFNB1 RNA, DXVX QC
^a^Tulane University of Louisiana, U54CA260581	Tulane University COVID Antibody and Immunity Network (TUCAIN)	Prospective cohort study	□ Adults living with solid and liquid cancers, adults with HIV, children with asthma, adults, and children with a history of SARS-CoV-2 infection or vaccination; □ >6 mo; M/F; □ White, black, Hispanic □ Southeast Louisiana	1600	04/2020–12/2025; Time 0, 1, 2, 4, 6 months then every 6 months after each immune event (eg, SARS-CoV-2 infection or vaccination); Survey and blood collection	Plasma, PBMCs; ELISA for anti-SARS-CoV-2 N, S and RBD Ab, T-cell epitopes studies, pseudovirus neutralization assays, antibody function assays
University of Alabama at Birmingham, Heersink School of Medicine U01CA260462	Adaptive Immunity and Persistent SARS-CoV-2 Replication	Prospective cohort study	□ Children undergoing cancer chemotherapy or other immunomodulatory treatment with COVID-19 □ healthy children with COVID-19 as controls □ 3 months to 18 years, M/F □ White, non-Hispanic, black, Hispanic □ Alabama	300	9/2020– 8/2024 0, 1, 3, 6 months for blood samples Weekly NP swab collection until 2 negative COVID PCR In-person interview, medical record abstraction	Whole blood; Plasma ELISA for IgG binding antibodies, neutralizing antibody assays using ACE2 binding inhibition and pseudovirus particles; NP swabs—RT-PCR for the detection of SARS-CoV-2 RNA
University of Arkansas for Medical Sciences, Fay W. Boozman College of Public Health, U01CA260526	The DISCOVAR Study: Disparities in Immune Response to SARS-CoV-2 in Arkansas	Prospective cohort study	□ Adult residents of Arkansas with COVID-19; □ >18 yo, M/F, □ White/Non-Hispanic black/Hispanic, □ Arkansas	600	4/2021–12/2025; Time 0, 1, 2, 3, 6, 12, 18, 24, 30, 36, 42, 48 months; Telephone, video or in person interview; medical records	SARS-CoV-2 antibody assays on serum and dried blood spots
University of Massachusetts Chan Medical School, U01CA261276	Enhancing Racial and Ethnic Diversity in COVID-19 Immunology Research Participation Through Storytelling (COVIDStory)	Randomized control trial	□ Black and Hispanic community members; □ >18 yo, M/F, □ Black/African American or Hispanic/Latinx, □ Central Massachusetts	1920	10/2021–8/2022 Survey in Qualtrics and RedCap: blood collection at Time 0.	Plasma; ELISA and/or LUMINEX screening for SARS-CoV-2 N, S, RBD IgG and IgA antibodies among other common viral infections such as the common human CoVs (OC43, LN63, 229E, and HKU1) influenza, EBV, and CMV
University of Minnesota, CBC21X091	Serological Sciences Network Capacity Building Center	Repeated measurement longitudinal cohort	□ HIV patients, cancer survivors, solid organ and hematopoietic transplant patients, and immunocompetent adults; □ >18 yo, M/F; □ Any race/ethnicity □ Minnesota	600 in each of the immunocompromised groups and 300 in the immunocompetent group.	06/2021– 12/2023 Prevaccine, 1-month postboost dose, then every 3–6 months; Medical records	Serum, plasma, PBMCs/ELISA, automated immunoassay (Roche Cobas), University of Minnesota in-house developed spike total anti-RBD antibody method with IgG titers, and Roche nucleocapsid qualitative method (to assess for natural infection)
^a^University of North Carolina at Chapel Hill, U54CA260543	North Carolina SeroNet Center for Excellence COVID-19 Household Transmission (CO-HOST) Observational Cohort of COVI-19 (OBS-C) COVID-19 in Farm and Food processing workers in North Carolina (COFF-NC) Covid-19 Convalescent Plasma Donor Biobank (CCP) Coronavirus-Inactivating Plasma (CoVIP) Recipient Biobank Adaptive Immune and Mucosal Responses in Covid-19 Recovered Individuals and SARS-CoV-2 Vaccinated Individuals (AIM-CoV) Hospital Remnant Study (HRS-CoV) UNC COVID Pathobiology study	Longitudinal cohort Cross-sectional cohort	□ Individuals with a positive test for SARS-CoV-2 infection (OBS-C); Households with persons with COVID-19 (CO-HOST); Farm and food processing workers (COFF-NC); Individuals with a positive test for SARS-CoV-2 infection who donated convalescent plasma (CCP) or received convalescent plasma (CoVIP) as part of a clinical trial; Individuals who have received a SARS-CoV-2 vaccine (AIM-CoV); NC-laboratory remnant samples from outpatient and inpatient clinics from April 2020 to June 2021 (HRS-CoV). □ All ages and sexes □ Any race/ethnicity □ Central North Carolina	OBS-C: 53, CO-HOST: 308 COFF-NC: 224 CCP: 201 CoVIP: 55 AIM-CoV: up to 200 HRS-CoV: 12 471 Pathobiology: 188	04/2020–2026; variable durations of follow up (28 days to 1 year); HRS-CoV was cross-sectional Surveys Medical record review	Serum; Plasma;Whole blood; Nasopharyngeal swabs; Anterior nasal turbinate swabs; Saliva; Throat wash; Sputum; Tracheal aspirate; Urine; Stool
University of Puerto Rico Medical Sciences Campus, Puerto Rico Science, Technology and Research Trust, La Jolla Institute of Technology, U01CA260541	SARS-CoV-2 Correlates of Protection in a Latino-Origin Population	Cross-sectional study	□ COVID-19 patients in Puerto Rico and vaccinated patients; □ >18 yo, M/F; □ Any race/ethnicity □ Puerto Rico	30 000	11/2020–7/2025; Baseline, 2 weeks; Survey, medical records	Nasopharyngeal swabs, whole blood; LDA ELISA-based IgM/IgG tests
Yale University, U01CA260507	Immuno-Serological Assays for Monitoring COVID19 in Patients with Hematologic Malignancies	Retrospective cohort study	□ >18 yo, M/F; □ Any race/ethnicity and African Americans in the New Haven area □ New Haven County	~300	11/2020– 10/2025; Samples will be collected at prevaccine, and 1 month, 3 months, 12 months and 24 months postvaccination	Microfluidic barcode chip for high-plex serology assay; Microfluidic barcode chip for high-plex plasma protein assay; CodePlex assay for multiplex cytokine assay commercially available at IsoPlexis; IsoCode assay of single-cell cytokine signature commercially available at IsoPlexis; Single-cell RNA-seq commercially available at 10× Genomics; Single-cell TCR/BCR sequencing available at 10× Genomics; CyTOF assay for multiplex immunophenotyping commercially available at Fluidigm
Nonepidemiologic SeroNet Studies						
Beth Israel Deaconess Medical Center, U01CA260476	Immunologic Signatures of SARS-CoV-2 vaccination and disease					
Harvard T Chan School of Public Health, 1U01CA261277	Causal, statistical, and mathematical modeling with serologic data					
La Jolla Institute For Immunology, U01CA260588	SARS-CoV-2-reactive tissue-resident memory T cells in healthy and cancer subjects					
^a^Stanford University, U54CA260517	Mechanisms and duration of immunity to SARS-CoV-2					
Wadsworth Center, U01CA260508	High-throughput dried blood spot (HT-DBS) technologies in SARS COV-2 serology and vaccinology					

Abbreviations: Ab, antibody; Ag, antigen; BCR, B-cell receptor; CMV, cytomegalovirus; COVID-19, coronavirus disease 2019; EBV, Epstein-Barr virus; HIV, human immunodeficiency virus; HRS, Hospital Remnant Study; IBD, *inflammatory bowel disease; IFN, interferon; Ig, immunoglobulin; IL, interleukin;* NC, North Carolina; NGS, next-generation sequencing; *NP,* nasopharyngeal; PBMC, peripheral blood mononuclear cells; RT-PCR, reverse-transcription polymerase chain reaction; SARS-CoV-2, severe acute respiratory syndrome coronavirus 2; TCR, T-cell receptor; TNF, tumor necrosis factor; WT, wild type; yo, years old.

Centers of Excellence (n = 8).

##### Individuals With Immune-Mediated Inflammatory Diseases

SeroNet studies are focused on populations with rheumatoid arthritis, systemic lupus erythematosus, and inflammatory bowel diseases (Crohn’s Disease and ulcerative/indeterminate colitis). Adults with immune-mediated inflammatory diseases face significant concerns regarding infection risk, continuity in clinical care, and potentially suboptimal vaccine response. Additional concerns include disease exacerbation with either infection or vaccination and poor infection outcome, in both cases owing to heightened autoimmunity [7–10]. Key findings have been reported on (1) the frequency of adverse events after vaccination [11] and (2) the comparison of induced antibody responses across SARS-CoV-2 vaccine platforms [12], which help to inform clinical guidelines [13].

##### Individuals With Cancer

The SARS-CoV-2 infection continues to cause significant morbidity and mortality among vulnerable immunosuppressed cancer patients. For example, patients with lung cancer have a greater than 7-fold higher rate of becoming infected with SARS-CoV-2 COVID-19, a greater than 3-fold higher hospitalization rate with high complication rates, and an estimated case fatality rate of more than 30% [14]. The potential effects of malignancy and/or anticancer treatments on COVID-19 vaccine response as well as the impact of a vaccine on cancer treatment, incidence of adverse events, and progression are a main focus of some SeroNet studies. Several hundred patients with cancer, including hematological malignancies, solid cancers, and hematopoietic cell transplant recipients have been accrued and are being followed prospectively for endpoints of interest and impacts of various immunotherapies/cancer treatments. Key findings reported include perspectives and concerns regarding vaccination in cancer patients [15], and the reduced antibody response in cancer patients compared with healthy individuals [16, 17], in particular patients with selected hematological malignancies and those receiving specific anticancer treatments. For example, the seroconversion rate for patients with chronic lymphoblastic leukemia is as low as 50% compared with approximately 100% in the general population [18].

##### Individuals Undergoing Solid Organ Transplantation

Solid organ transplant recipients may receive a variety of immunosuppressive regimens to prevent organ rejection. Evaluating immune responses to different COVID-19 vaccines among solid organ transplant recipients is a specific focus of 2 SeroNet centers. Most transplant recipients evaluated in these studies include recipients who have received kidney, lung, heart, or pancreas transplantation. Detailed information on type of immunosuppressive medications and duration of immunosuppression is obtained from electronic medical records. Recent studies show substantially lower seroconversion rates among solid organ transplant recipients [19–22], and subsequent studies are focusing on both the initial rates of seroconversion and the durability of the immune response in these solid organ transplant recipients [23, 24].

##### Individuals With Human Immunodeficiency Virus 

People with human immunodeficiency virus (PWH) are at an increased risk of COVID-19 and severe disease manifestations [25, 26]. The effects of antiretroviral therapy or human immunodeficiency virus (HIV)-related immunosuppression on vaccine response are unclear [27]. In addition, PWH who are not immunocompromised may have immunological features that result in different B-cell or T-cell responses compared with immunocompetent HIV-negative individuals [28–30]. Published studies on the immune response to SARS-CoV-2 vaccination in PWH demonstrate that PWH can respond to vaccination, but these are limited by nonrandomization approaches and lack of heterogeneity in sex, race/ethnicity, and age [27, 31, 32]. Therefore, further studies of humoral and cellular immunity and safety profiling after completion of the vaccine series in PWH are needed [31].

##### Individuals at Risk Due to Occupational Exposures

Healthcare workers (HCWs) have historically been on the front lines of epidemics [33]. The SARS-CoV-2 is a highly transmissible respiratory virus, making hospitals potential loci for outbreaks and placing HCWs at high risk of acquiring the infection and unknowingly transmitting of the virus to others. To track seroprevalence or SARS-CoV-2 antibodies and vaccine-induced immune response in HCWs, SeroNet studies at major academic centers have recruited several hundred HCWs for longitudinal assessments. Key studies to date include understanding the magnitude of neutralizing antibody titers among polymerase chain reaction-positive HCWs, intensive care unit patients, and convalescent plasma donors [34] and the diverse impact of these neutralizing antibodies to different variants of COVID-19 [35].

##### Pregnant Women

Prevention and control of COVID-19 infection among pregnant women have been a major concern during the pandemic, primarily because pregnancy is a risk for more severe COVID-19 outcomes for both mother and baby [36, 37]. Studies are underway to investigate the clinical characteristics, outcomes, and vertical transmission (of infection or antibodies postinfection or postvaccine). In addition, studies are being conducted to determine the best time during pregnancy to administer vaccines to protect the mother and optimally transplacentally transfer antibodies to the baby.

##### Children, Teens, and College Students

Although several studies show children and adolescents are at lower risk of COVID-19-related morbidity and mortality [38], multisystem inflammatory syndrome in children is a serious health condition associated with SARS-CoV-2 infection [39]. Given the rarity of this condition, large consortium efforts involving SeroNet will be helpful in better understanding risk factors, clinical course of the disease, and immune response to vaccination. Recent studies have highlighted (1) racial and socioeconomic disparities of SARS-CoV-2 infection among the pediatric population [40] and (2) virological characteristics of hospitalized children with infection [41]. Children undergoing cancer chemotherapy or receiving other immunomodulatory treatments are being enrolled to understand the immune responses against SARS-CoV-2 after infection and immunization.

### Ethics Approval and Participant Consent

The design of the work has been approved by local ethical committees for each individual study. This overview paper summarizing the consortium does not include factors necessitating patient consent.

## DISCUSSION

### Health Disparities: Race/Ethnicity, Sex, and Age

Health disparities among racial/ethnic minority groups are a persistent and growing public health concern. Although initially expected to be “the great equalizer,” COVID-19 has instead reinforced and exacerbated racial/ethnic health disparities in the United States [42]. The COVID-19 pandemic has emphatically demonstrated that minority populations are disproportionately exposed to infection and experience a greater burden of disease [43, 44]. Several reasons for these differences have been proposed, including a higher prevalence of comorbidities (eg, type 2 diabetes), greater social deprivation, large multigenerational households, differences in occupational risk, misinformation, and inequitable access to COVID-19 resources and healthcare. To address these concerns, specific SeroNet studies are engaging community leaders and focusing on the recruitment and retention of ethnic/racial minority groups throughout the United States across a spectrum of socioeconomic levels in our research studies.

The pandemic has also revealed disparities based on age, sex, and gender [45–48]. Worldwide, people who are older aged or male sex are at greater risk of more severe outcomes from COVID-19 [48]. Age and biological sex also impact innate, humoral, and cell-mediated immune responses during infection [49–51]. This is further reflected in specific SeroNet study populations utilizing electronic medical records, with elevated inflammatory biomarkers explaining a majority of the sex differences in COVID-19 outcomes among hospitalized patients [51]. How sex and age intersect to alter immunity to SARS-CoV-2 infection and vaccination is being considered in SeroNet studies and collaborations.

### Data Sharing

To accelerate data dissemination, SeroNet research results and data sets are made publicly available at the time a manuscript (“study”) is accepted for publication in a peer-reviewed journal. Rapid data sharing ensures transparency and accessibility and facilitates confirmation of the research findings, thus accelerating generalizability of the results. Furthermore, it promotes (1) new analytical strategies to answer other research questions and (2) the creation of harmonized datasets by combining data from multiple sources, with predetermined common data elements to facilitate meta-analyses. To ensure all data generated through the SeroNet program can be easily located, all studies will also be registered in the Immunology Data Portal, ImmPort [52], an immunology domain-specific data repository supported by NIAID. The ImmPort data model is designed to accommodate data and metadata from common types of immunology assays including enzyme-linked immunosorbent assays, flow cytometry, cytology by time-of-flight, chemiluminescence immunoassay, electrochemiluminescence immunoassay, Luminex, MesoScale Discovery, or IsoPlexis multiplex assays, and many others. ImmPort also allows linking data to other repositories, such as datatype-specific repositories, including NCBI’s dbGaP, SRA, or GEO. A SeroNet study record in ImmPort will contain the metadata and data deposited in ImmPort, as well as any links to data deposited in other public repositories.

## DISCUSSION

### Strengths and Limitations

The swift emergency appropriation passed by the US Congress in April 2020 provided funding within months that enabled the development of the SeroNet infrastructure. SeroNet is a unique network comprising a broadly based multidisciplinary consortium of researchers that fosters collaboration among immunologists, epidemiologists, virologists, clinicians, clinical laboratories, behavioral and social scientists, policymakers, and community members. By harnessing existing academic medical research centers and creating new relationships between institutions and investigators (eg, connecting infectious disease immunologists with oncology or autoimmune disease-focused immunologists or epidemiologists), this program is building long-lasting bridges and initiating a new vision for multidisciplinary research programs.

Within the network, we have defined (1) common data elements for self-reported data and clinical treatment/outcomes and (2) standardization of serological and cellular/molecular assays, thereby facilitating data harmonization for future consortium-wide efforts. All researchers pledged commitment to data sharing and accessibility using the F.A.I.R. (findable, accessible, interoperable, reusable) principles [53]. Moreover, the rapid dissemination of publications in OpenAccess format and the nimble and evolving nature of cohorts allow investigators to adapt to critical research questions. Furthermore, given the large size of the network, data can be pooled across studies to investigate rare exposures and outcomes.

Limitations of the network include the inherent heterogeneity across study methodologies. The network is also unable to investigate international variation and immune responses to vaccines not available in the United States. Finally, as the COVID-19 pandemic evolves, there will be a need for additional data collection not anticipated. However, the large infrastructure and diverse expertise of this multi-institutional effort should allow for sufficient nimbleness to address the ever-changing nuances of this pandemic.

### Future Directions and Impact

The heterogeneity of clinical severity and the different manifestations observed after SARS-CoV-2 exposure suggest that both the viral pathogenesis and host responses are exceedingly complicated and will necessitate long-term studies. Furthermore, rapid deployment of different types of vaccines and changes in public policies, including the availability of vaccines, affect recommendations for the number of vaccine doses (and timing) needed to sustain immunological protection across various populations. SeroNet is in an optimal position to gather such data and answer critical scientific questions on these topics as they arise. Outstanding questions include understanding correlates of protection, identifying vulnerable populations for booster vaccinations, and alternative strategies for “poor responders.” Factors that increase the durability of vaccine-elicited immune responses in the general population and whether all persons require subsequent vaccination are unclear. Future priorities for investigation include the following: (1) the potential benefit of heterologous vaccinations; (2) deep phenotyping of the spectrum of “at-risk” subpopulations with detailed clinical annotation to identify pathogenic mechanisms; (3) investigation into diversity of immune response across subpopulations and their respective roles in protection from infection and/or disease; and (4) interactions with other common respiratory pathogens and putative cross-protection.

## CONCLUSIONS

In summary, SeroNet represents an ambitious effort to coordinate the study of this infection in real time. This publication brings information about this network forward, with the goals of articulating our framework for epidemiologic and immunologic study of SARS-CoV-2 and human populations and highlighting the value of creating a pan-national research network to combat the COVID-19 pandemic. The longitudinal studies of human populations already initiated establish critically important early benchmarks for tracking the host immunologic response to both the SARS-CoV-2 virus and to vaccination through time. The principles of making SeroNet datasets publicly available will help drive discovery and serve as a model for future research on both novel and existing diseases when multidisciplinary, collaborative research is desired in an evolving environment.

## Supplementary Data 

Supplementary materials are available at *Open Forum Infectious Diseases* online. Consisting of data provided by the authors to benefit the reader, the posted materials are not copyedited and are the sole responsibility of the authors, so questions or comments should be addressed to the corresponding author.

ofac171_suppl_Supplementary_Appendix_A

ofac171_suppl_Supplementary_Appendix_B
