# Supplementary material for: Mission, Organization, and Future Direction of the Serological Sciences Network for COVID-19 (SeroNet) Epidemiologic Cohort Studies
Source: Open Forum Infect Dis. 2022 Apr 27;9(6):ofac171. doi: 10.1093/ofid/ofac171 (PMC9129196; doi:10.1093/ofid/ofac171)
Supplement: ofac171_suppl_Supplementary_Appendix_A [file ofac171_suppl_supplementary_appendix_a.pdf]

Appendix A

Study Design Template

SeroNet
Template for SARS-CoV-2 post-vaccine surveillance studies in cancer/immunocompromised individuals

Purpose:
Provide a common study template that could be used within SeroNet and beyond for the serosurveillance of cancer/immunocompromised patients receiving a SARS-CoV-2 vaccine. The template could be applicable to prospective interventional trials when investigators have access to vaccines for experimental use, or for observational studies where individuals receive vaccines as they become available in the community.

Population can include:
- Newly diagnosed cancer patients
- Patients currently undergoing treatment for cancer, including chemotherapy, radiation therapy, immunotherapy, targeted therapy, or combinations of therapies
- Individuals with a history of cancer/cancer survivors
- Immunocompromised individuals
- Individuals with autoimmune diseases

This document is intended to be agnostic as to which vaccination individuals receive and will provide recommendations for:
- Population
  - Recommend focus on a particular population if possible. For example, a specific cancer type, autoimmune disease, or individuals treated with certain therapeutics.
  - Inclusion of a control group, or consideration for an external control group using the same timepoints and assays.
- Specimen selection and collection
  - Timepoints for vaccination and collection
  - Specimen types
  - Specimen collection protocols
  - Key determinants to be considered
- Assays
  - Type
  - Benchmarking/standardizing
  - Standard protocols where available
- Data collection and common data elements (CDEs)
  - Demographics
  - Clinical characteristics
    - Including cancer CDEs as well as for other co-morbidities
  - Vaccine kinetics

Design Considerations:

- Vaccine responses can be influenced by a number of factors and conditions
    - Cancer and autoimmune disease and their treatments could affect immunocompetence and therefore vaccination responses.
    - What is this impact of cancer and/or cancer therapy on response to the vaccine and conversely, what is the impact of the vaccine on cancer treatment or cancer progression?
    - Individuals with cancer are a complex and extremely diverse population and there are a multitude of considerations, including approaches to capturing the appropriate clinical information regarding an individual's cancer type, subtype, stage, treatments/regimen (chemotherapy, radiation therapy, immunotherapy, surgery), time since diagnosis, timing of therapy, etc.
    - Individuals with autoimmune diseases could experience flareups or other adverse reactions following vaccination.
- Recommendations for when specimens should be collected for optimal tracking of the vaccine induced immune response vs. those timepoints that are important from a cancer treatment perspective.
    - Inclusion of additional sampling timepoints following a documented infection (positive PCR) after vaccination.
    - Inclusion of additional sampling timepoints when adverse events are observed.
- The inclusion of a healthy control group within the study, or identification of an external control group using similar collection and testing procedures.
- Collection of non-cancer related clinical data performance status, chronic diseases and specific therapies, co-morbidities, tobacco history (particularly lung cancer), alcohol history, other medications, etc.
- Collection of quality-of-life considerations and patient reported outcomes

Scientific and Clinical Questions

- Anamnestic response is likely to be blunted in cancer/immunocompromised population as measured by antibody titer.
    - The degree of compromise in antibody responses and decay is unknown.
    - Specific populations and treatments should be correlated with antibody kinetics.
- Changes in incidence of adverse events (vaccine or cancer related).
- Considerations for the spacing of vaccine doses or need for an additional boost to confer protective "titers".

Timeline and specimen collection

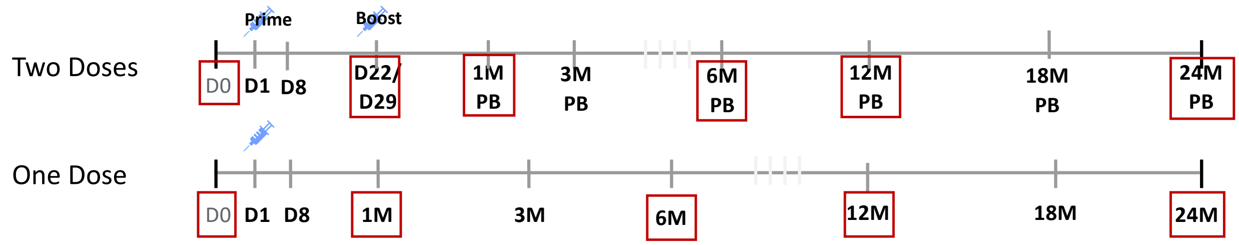

Figure 1. *Recommended timepoints for the collection of biospecimens following vaccination with a two dose or single dose vaccine. The red box indicates the recommended minimal timepoints for specimen collection to measure vaccine immune kinetics.*

Dependent upon the needs of the study, this template provides guidance about the core or minimum number of timepoints and sample types to collect post-vaccination for meaningful interpretation of the immune response to the vaccine. Note that the D0/D1 timepoint is intended to establish a baseline and samples should be collected prior to vaccination. This sample collection can occur on the same day as the vaccination, or up to one week before a planned vaccination.

It is also recommended to collect a nasal swab at the time other specimens are collected for measuring active infection/viral shedding.

Table 1. *Recommended collection timepoints per specimen type. PB denotes post-boost timing, for single dose vaccines the timepoints PB should be considered as well (1-, 3-, 6-, 12-, 18-, and 24-months post vaccination). The timepoints highlighted in red and with an X denote the minimum recommended specimen collection per timepoint. Other sample collection timepoints within the table are recommended for additional collections based on study aims.*

Sample Type	D0/D1 (Prime)	D4	D8	D22/29 (Boost)	1M (PB)	3M (PB)	6M (PB)	12M (PB)	18M (PB)	24M (PB)
Serum	X			X	X		X	X		X
Plasma										
PBMC	X			X	X					
DBS										
Saliva										
Nasal Swab	X			X	X		X	X		X

Table 2. *Recommended assays per timepoint following vaccination. X denotes the minimum recommended timepoints per assay type. Other sample collection timepoints within the table are recommended for additional assays depending on study aims.*

Assay Type	D0/1D1 (Prime)	D4	D8	D22/29 (Boost)	1M PB	3M PB	6M PB	12M PB	18M PB	24M PB
Serology (LBA)	X			X	X		X	X	X	X
Serology (Neutralization Assay)	X			X	X			X		
Immune Cell Repertoire	X	X	X	X	X					
ELISPOT	X		X	X	X	X				
Intracellular Cytokine Staining	X		X	X	X	X				
Assays for innate immunity	X	X	X							

Table 3. *Immune assays by sample type*

Assay	Serum	Plasma	PBMC	DBS	Saliva	Nasal Swab
Serology (LBA)	X	X		X	X	
Serology (Neutralization Assay)	X	X			X	
Immune Cell Repertoire			X			
ELISPOT			X			
Intracellular Cytokine Staining			X			
Assays for innate immunity	X	X	X			
SARS-CoV-2 Diagnostic Test						X

Assay Types

The SeroNet Operations (Ops) groups will be key to collecting information about assays that are used by SeroNet investigators, including validation and regulatory status, and the intended use for clinical or research studies with the goal of developing standard or harmonized assays to enable cross-laboratory and study analyses. The Serology Assays Ops group is currently planning on conducting a comparative study for high-throughput immune monitoring assays, in particular ligand binding assays (LBA), to determine assay performance characteristics and evaluate the suitability for the intended use.

Sample collection/preparation protocols are dependent upon the assay type. The attached documents provide standard procedures from the FNL Serology Lab for:

- Serum biospecimen processing
- PBMC isolation and cryopreservation

It is recommended to use assays that have been well characterized and validated. Below is a list of recommended assay types and protocols as available at the time of document completion.

- **Serology (LBA):** To distinguish natural infection from vaccine induced immunity we recommend performing an IgG LBA against both the Spike (natural infection and vaccine-mediated) and N (natural infection) proteins.
  - It is recommended to perform an FDA EUA quantitative assay.
  - When using an in-house assay provide information about the sensitivity and specificity of the assay compared to the National standard available from the FNL Serology Lab.
- **Serology (neutralization assay):** To determine the presence and magnitude of functional, neutralizing SARS-CoV-2 antibodies as well as correlation with ligand binding assay results. This is a more laborious assay and can be considered at limited timepoints or in limited subsets.
- **Immune cell assessments:** Disease related abnormalities and treatments for cancer and autoimmune disease can impact immune cell populations/ immunocompetence and potentially affect the response to vaccination. Therefore, especially a baseline immune assessment will be critical for the correlation of immune kinetics to immune status.
  - Assay types can include flow-cytometry and CyTOF.
- **ELISPOT**: Quantitative assay that measures cytokines released from antigen stimulated T cells.
- **Intracellular cytokine staining (ICS):** Flowcytometry-based assay that allows for simultaneous cellular phenotyping and single cell cytokine detection used to assess T cell responses.
- **Innate immune cell measurement assays**:
  - This template does not make specific assay recommendations to measure the innate immune response (note that the immune cell repertoire profiling can include assessment of innate immune cell populations).
- **SARS-CoV-2 diagnostic test**: PCR-based assay to detect viral RNA.
  - Recommend using an FDA EUA assay.

Common Data Elements

To facilitate SeroNet-wide data analyses the use of a minimum set of common data elements (CDEs) are recommended. Some data elements are recommended to be collected at each timepoint/encounter if feasible, others can be collected following consent at the beginning of the study. CDEs are roughly divided by:

- Demographics
- Clinical characteristics

- Patient reported outcomes
- Assay results

See the spreadsheet for draft SeroNet Common Data Elements

**SOP Title:** Serum Biospecimen Processing Procedure (NCI SeroNet Guidance Document)		
**Document ID: VIC_LAB_002**	Version	2.0
**Page 1 of 12**	Supersedes	1.0

Released by / Effective Date: Angelina C. Richards -S (Affiliate) Digitally signed by Angelina C. Richards -S (Affiliate) Date: 2020.11.24 17:34:25 -05'00'

**Written by:**		
Printed Name:	Title:	Signature/Date:
Troy Kemp	Scientific Manager	Troy J. Kemp -S (Affiliate) Digitally signed by Troy J. Kemp -S (Affiliate) Date: 2020.11.24 16:44:42 -05'00'

**Approved by:**		
Printed Name:	Title:	Signature/Date:
Debra Hope	NCI SeroNet Project Manager	Debra A. Hope -S (Affiliate) Digitally signed by Debra A. Hope -S (Affiliate) Date: 2020.11.24 16:52:24 -05'00'
Ligia Pinto	Director	Ligia A. Pinto -S (Affiliate) Digitally signed by Ligia A. Pinto -S (Affiliate) Date: 2020.11.24 17:25:14 -05'00'
**QA Approved by:**		
Printed Name:	Title:	Signature/Date:
Angelina Richards	QA Specialist III	Angelina C. Richards -S (Affiliate) Digitally signed by Angelina C. Richards -S (Affiliate) Date: 2020.11.24 17:34:07 -05'00'

This document contains confidential and proprietary information. Do not copy or distribute without prior, written permission.

1. PURPOSE

1.1. This GUIDANCE DOCUMENT is designed to explain how to process serum biospecimens.

1.2. This GUIDANCE DOCUMENT is intended to convey the process parameters and practices to be followed by each institute associated with the National Cancer Institute (NCI) Serology Network (SeroNet).

2. SCOPE

2.1. This document applies to all institutes associated with SeroNet through collaborations, grant funding, subcontracts, etc. that perform biospecimen processing.

2.2. This procedure does not describe the biospecimen collecting process. The biospecimen collecting process is dictated by the institute's protocol.

3. REFERENCES

3.1. VIC_GL_002: Shipping SARS-CoV-2 Associated Specimens to the FNL Central Repository (NCI SeroNet Guidance Document)

3.2. VIC_GL_003: Key Entity Identifier Assignment (NCI SeroNet Guidance Document)

4. RESPONSIBILITIES

4.1. It is the responsibility of the institute performing the serum biospecimen processing to:

4.1.1. Perform biospecimen processing using the indicated reagents, materials, equipment and process parameters in this guidance document.

4.1.2. Ship the processed biospecimens to the Frederick National Laboratory for Cancer Research (FNL) Central Repository following "VIC_GL_002: Shipping SARS-CoV-2 Specimens to the FNL Central Repository (NCI SeroNet Guidance Document)."

4.2. It is the responsibility of the Vaccine, Immunity and Cancer Program (VIC) to:

4.2.1. Generate, review and approve the biospecimen processing guidance document.

4.2.2. Distribute the most current version of this guidance document to each institute associated with SeroNet.

5. DEFINITIONS

5.1. Biospecimen - a sample of biological material, such as urine, whole blood, blood components, tissue, cells, DNA, RNA, and protein.

5.2. Severe Acute Respiratory Syndrome Coronavirus 2 (SARS-CoV-2)

6. REAGENTS, MATERIALS AND EQUIPMENT

6.1. Equipment

6.1.1. Class II Biosafety Cabinet (BSC)

6.1.2. -80°C Freezer

6.1.3. 2-8°C Refrigerator

6.1.4. Benchtop Centrifuge

6.1.5. Serologic Pipette

6.1.6. Pipette

6.2. Consumables

Note: Consumables requiring approval for use as “equivalent” by the NCI SeroNet are indicated with an Asterisk (*).

6.2.1. SeroNet specified 5 mL sterile tubes (Fisher Scientific, Cat #12-565-291 or equivalent*)

6.2.2. 125 mL Media Storage Bottle (Thomas Scientific, Cat # 19A00M420 or equivalent)

6.2.3. 250 mL Media Storage Bottle (Thomas Scientific, Cat # 19A00M421 or equivalent)

6.2.4. Pipette Tips

6.2.5. Serological Pipets

6.2.6. Blood Collection Tubes (Vacutainers)

6.2.6.1. Serum tube glass (BD, Cat # 366430 or equivalent*)

6.2.7. 4” Box and 81 position insert, or equivalent

6.2.8. Labels that can withstand temperatures ≤ -80°C

6.2.8.1. Example: Brady Label (Anthony-Lee Associates, Cat # THT-133-461-SLIT)

7. HEALTH AND SAFETY CONSIDERATIONS

Note: Each institute's Environment, Health, and Safety department will provide definitive measures for safety when processing human biospecimens as these considerations are provided only as a guideline.

7.1. Proper safety precautions should be taken while working in a laboratory setting. This includes, but is not limited to, proper protective equipment such as lab coats, safety glasses, closed-toe shoes, and non-latex gloves.

7.2. If SARS-CoV-2 positive samples are being processed, additional protective equipment is worn such as double layer of non-latex gloves and disposable arm sleeves.

7.3. A face mask is part of the standard personal protective equipment for the laboratory during the SARS-CoV-2 pandemic.

7.4. Follow the institute governed Biosafety Level 2 (BSL-2) requirements for handling and processing human biospecimens.

7.5. All human biospecimen processing work is performed inside of a Class II BSC.

7.6. Refer to the respective Safety Data Sheet (SDS) when working with any chemicals.

7.7. Refer to the institute's processes for disposing of biohazardous and chemical waste.

8. PROCEDURE PRINCIPLES

8.1. Refer to "VIC_GL_003: Key Entity Identifier Assignment (NCI SeroNet Guidance Document)" for process of assigning IDs to biospecimens and biospecimen aliquots.

8.2. Image of form "VIC_LAB_002.01, Serum Biospecimen Processing Form" is attached for institute's reference. The minimum information requiring documentation during the performance of the processing of the serum biospecimen is included in this form. See Attachment 1.

8.3. Image of form "VIC_LAB_002.02, Serum Biospecimen Collection Form" is attached for institute's reference. The minimum information requiring documentation during the collection of the blood biospecimen for serum processing is included in this form. See Attachment 2.

8.4. It is preferred that all equipment used in this process be maintained, at minimum, per the equipment manufacturer's recommendations.

8.5. It is preferred that all Pipettes, Laboratory Freezers and Refrigerators, and Benchtop Centrifuges used in this process be calibrated by a vendor or other qualified party.

8.6. It is preferred that all Laboratory Freezers and Refrigerators used in this process be monitored for temperature by a temperature monitoring system.

This document contains confidential and proprietary information. Do not copy or distribute without prior, written permission.

8.7. All human biospecimen handling is performed in a Class II Biosafety Cabinet (BSC) except for centrifugation and storage.

9. SERUM SEPARATION

Note: The maximum allowable time from blood collection (processing serum) to storage in a -80°C freezer is 8 hours.

9.1. Once blood biospecimen is received, allow the blood to clot upright at room temperature for 30-60 minutes.

9.2. If the blood biospecimen cannot be centrifuged immediately after the clotting time, refrigerate tubes at 2-8°C for up to 4 hours.

9.3. Label 5 mL sterile tubes for each serum biospecimen being processed. Use Attachment 3 for label specifications.

Note: The labels are expected to be printed by each Capacity Building Center (CBC) according the example in Attachment 3.

9.3.1. Biospecimen Aliquot ID: Refer to VIC_GL_003 for biospecimen aliquot ID assignment process. **Use Deidentified Biospecimen Aliquot ID Only**.

9.3.2. Biospecimen Type: Human Serum

9.3.3. Volume in milliliters (mL).

9.4. Rack the labeled tubes and set aside.

9.5. In a BSC, load blood biospecimen tubes into the centrifuge buckets and add the biohazard dome.

9.6. Centrifuge blood biospecimen tubes for 20 minutes at 1300 x g at 20-25°C.

Note: In case of catastrophic failure such as broken rotor, bucket, or biohazard dome during centrifugation, allow the centrifuge to sit for 30 minutes after it has stopped. Prior to inspection, consult with the clinic's Safety Department for best practices of biohazard clean up.

9.7. Following centrifugation, transport the centrifuge buckets with the biohazard dome to the BSC, and unload blood biospecimens in the BSC.

9.8. Carefully collect the top serum layer with a pipette. Do not disturb the buffy coat layer.

Note: Be very careful not to pick up red blood cells. Keep pipette above the red blood cell layer and leave a small amount of serum in the tube.

This document contains confidential and proprietary information. Do not copy or distribute without prior, written permission.

Effective Date: 24Nov20 Uncontrolled Print Copy For Reference Use Only

9.9. Place serum into a sterile Media Storage bottle. Vials from a single research participant are pooled together.

9.10. Mix by inverting the bottle 10 times.

9.11. Pipette 4.5 mL serum into the labeled sterile 5 mL tubes.

9.12. Label box(es) using label specification in Attachment 3.

9.13. Place aliquots into labeled box(es).

9.14. Store in -80°C freezer.

9.15. Ship specimens on dry ice to the FNL Central Repository following VIC_GL_002.

10. ATTACHMENTS

10.1. Attachment 1: VIC_LAB_002.01, Serum Biospecimen Processing Form

10.2. Attachment 2: VIC_LAB_002.02, Serum Biospecimen Collection Form

10.3. Attachment 3: Vial Label and Box Label

11. REVISION HISTORY

Version	Change	Reason
1.0	New guidance document for specimen processing by SeroNet organizations.	Currently no procedure; new initiative requiring communication of expectations.
2.0	1. Changed “specimen” and “sample” to “biospecimen” throughout document.<br>2. Minor formatting and grammatical changes throughout document.<br>3. Added Biospecimen and SARS-CoV-2 to new Definitions section.<br>4. Added VIC_GL_003 to References section.<br>5. Added SARS-CoV-2 and pandemic specific health/safety guidelines to Health and Safety Considerations section.<br>6. Added reference to VIC_GL_003, reference to new form, reworded equipment requirements to be “preferred” in the Procedure Principles section.<br>7. New form VIC_LAB_002.02 to capture biospecimen collection.	1. Consistency between documents and database verbiage.<br>2. Clarification.<br>3. Clarification.<br>4. Referenced in body of procedure.<br>5. Clarification.<br>6. Clarification.<br>7. Ease of use.

	8. Revised form VIC_LAB_002.01 to capture serum processing. Reformatted to accommodate processing of more than one biospecimen at one time.	8. Ease of use.

Attachment 1: VIC_LAB_002.01, Specimen Processing Form

Frederick National Laboratory for Cancer Research<br>sponsored by the National Cancer Institute		Vaccine, Immunity and Cancer Program<br>Standard Operating Procedure<br>Form	
**Form Title:** Serum Biospecimen Processing Form			
**Document ID: VIC_LAB_002.01**		Version:	2.0
Associated SOP: VIC_LAB_002		Effective Date:	
Supersedes:	1.0	**Page 1 of 3**	

Biospecimen Receipt

Serum Biospecimen Processing Laboratory Name:				
Biospecimen Number	Deidentified Biospecimen ID	Date Received	Time Received (24H)	Initials
1				
2				
3				
4				
5				

Equipment

Equipment Name	Equipment ID	Calibration Due Date
BSC		
Centrifuge		
Pipette		
☐ N/A Pipette		
☐ N/A Pipette		
☐ N/A 2-8°C Refrigerator		
☐ N/A -80°C Freezer		

Consumables

Consumable Name	Catalog Number	Lot Number	Expiration Date
☐ N/A 5 mL Sterile Tubes			
☐ N/A			

Verify current version prior to use. Use of a superseded or obsolete document is prohibited.

This document contains confidential and proprietary information. Do not copy or distribute without prior, written permission.

Verify current version prior to use. Use of a superseded or obsolete document is prohibited.

This document contains confidential and proprietary information. Do not copy or distribute without prior, written permission.

Effective Date: 24Nov20 Uncontrolled Print Copy For Reference Use Only

Frederick National Laboratory for Cancer Research
sponsored by the National Cancer Institute

Vaccine, Immunity and Cancer Program
Standard Operating Procedure
Form

Form Title: Serum Biospecimen Processing Form			
**Document ID: VIC_LAB_002.01**		Version:	2.0
Associated SOP: VIC_LAB_002		Effective Date:	
Supersedes:	1.0	**Page 2 of 3**	

Processing Steps

(√)	Process Step	
	Clot blood biospecimen for 30-60 mins at RT, upright.	
	Clot Start Time	Clot End Time
☐ N/A Step	If biospecimen is not processed immediately, store at 4°C for up to 4 hours.	
	4°C Storage Start Time	4°C Storage End Time
	Centrifuge blood biospecimen for 20 mins, 1300 x g, 20-25°C	
	Centrifuge time: ____________________mins.	
	Carefully collect the top serum layer.	
	Pool serum from single research participant.	
	Invert pooled serum 10 times.	
	Aliquot serum into labeled 5 mL sterile tubes.	
	Place aliquots into labeled box.	
	Store aliquots at -80°C.	

Biospecimen Number	Date/Time (24H) Blood Biospecimen Collected	Date/Time (24H) Serum Aliquots Stored at -80°C	Initials
1			
2			
3			
4			
5			

Verify current version prior to use. Use of a superseded or obsolete document is prohibited.

This document contains confidential and proprietary information. Do not copy or distribute without prior, written permission.

Frederick National Laboratory for Cancer Research<br>sponsored by the National Cancer Institute		Vaccine, Immunity and Cancer Program<br>Standard Operating Procedure<br>Form	
**Form Title:** Serum Biospecimen Processing Form			
**Document ID: VIC_LAB_002.01**		Version:	2.0
Associated SOP: VIC_LAB_002		Effective Date:	
Supersedes:	1.0	**Page 3 of 3**	

Serum Biospecimen Aliquot

Biospecimen Number	Number of Aliquots	Aliquot Volume (mL)	Example Biospecimen Aliquot Label
1			
2			
3			
4			
5			

Comments: ☐ N/A

Performed by/date:	
Reviewed by/date:	

Verify current version prior to use. Use of a superseded or obsolete document is prohibited.

This document contains confidential and proprietary information. Do not copy or distribute without prior, written permission.

Verify current version prior to use. Use of a superseded or obsolete document is prohibited.

This document contains confidential and proprietary information. Do not copy or distribute without prior, written permission.

Attachment 2: VIC_LAB_002.02, Serum Biospecimen Collection Form

Frederick National Laboratory for Cancer Research sponsored by the National Cancer Institute		Vaccine, Immunity and Cancer Program Standard Operating Procedure Form	
**Form Title:** Serum Biospecimen Collection Form			
**Document ID: VIC_LAB_002.02**		Version:	2.0
Associated SOP: VIC_LAB_002		Effective Date:	
Supersedes:	1.0	**Page 1 of 1**	

Deidentified Biospecimen ID:					
Biospecimen Group:	☐ Positive ☐ Negative ☐ Serosurveillance				
Section I. Vacutainer Collection Tube					
Catalog No.:					
Lot No.:	☐ N/A				
Exp. Date:					
Section II. Blood Biospecimen Collection					
Name of Clinic/Company:					
Date:		Time: (24 Hr)		Initials:	

Reviewed by/date: ______________________________

Verify current version prior to use. Use of a superseded or obsolete document is prohibited.

This document contains confidential and proprietary information. Do not copy or distribute without prior, written permission.

Verify current version prior to use. Use of a superseded or obsolete document is prohibited.

This document contains confidential and proprietary information. Do not copy or distribute without prior, written permission.

Attachment 3: Vial Label and Box / Rack Label

Vial Label

Aliquot ID
Biospecimen Type
Volume

Barcode:	Barcode linked to Biospecimen Aliquot ID
Line 1:	Deidentified Biospecimen Aliquot ID
Line 2:	Biospecimen Type (Serum)
Line 3:	Volume (mL)

Example Label:

A1_123456_123_1
Human Serum
4.5 mL

Box Label

Study: ?????? / ??????
Biospecimen Type: ?????
Date: DDMMMYY
Shipping ID: XXXXXXX
Box ? **of** ?

Line 1:	SeroNet
Line 2:	Biospecimen Type (Human Serum)
Line 3:	Date in DDMMMYY format
Line 4:	Shipping ID
Line 5:	Box Number

Example Label:

Study: SeroNet
Sample Type: Human Serum
Date: 01Jan20
Shipping ID: XXXXXXX
Box 1 **of** 10

Box Label Placement:

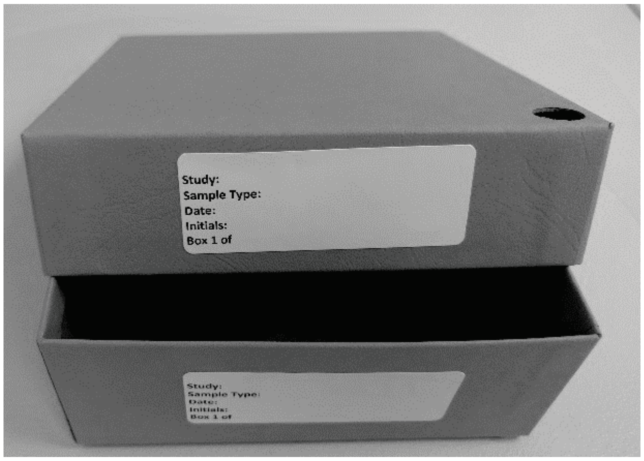

This document contains confidential and proprietary information. Do not copy or distribute without prior, written permission.

Released by / Effective Date: Angelina C. Richards -S (Affiliate) Digitally signed by Angelina C. Richards -S (Affiliate) Date: 2020.11.24 17:37:20 -05'00'

**Written by:**		
Printed Name:	Title:	Signature/Date:
Troy Kemp	Scientific Manager	Troy J. Kemp -S (Affiliate) Digitally signed by Troy J. Kemp -S (Affiliate) Date: 2020.11.24 16:46:41 -05'00'

**Approved by:**		
Printed Name:	Title:	Signature/Date:
Debra Hope	NCI SeroNet Project Manager	Debra A. Hope -S (Affiliate) Digitally signed by Debra A. Hope -S (Affiliate) Date: 2020.11.24 16:51:47 -05'00'
Ligia Pinto	Director	Ligia A. Pinto -S (Affiliate) Digitally signed by Ligia A. Pinto -S (Affiliate) Date: 2020.11.24 17:24:39 -05'00'
**QA Approved by:**		
Printed Name:	Title:	Signature/Date:
Angelina Richards	QA Specialist III	Angelina C. Richards -S (Affiliate) Digitally signed by Angelina C. Richards -S (Affiliate) Date: 2020.11.24 17:36:55 -05'00'

Verify current version prior to use. Use of a superseded or obsolete document is prohibited.

This document contains confidential and proprietary information. Do not copy or distribute without prior, written permission.

Effective Date: 24Nov20 Uncontrolled Print Copy For Reference Use Only

1. PURPOSE

1.1. This GUIDANCE DOCUMENT is designed to explain the process of isolating Peripheral Blood Mononuclear Cells (PBMCs) and freezing PBMCs for storage at -80°C or colder.

1.2. This GUIDANCE DOCUMENT is intended to convey the process parameters and practices to be followed by each institute associated with the National Cancer Institute (NCI) Serological Sciences Network (SeroNet).

2. SCOPE

2.1. This document applies to all institutes associated with SeroNet through collaborations, grant funding, subcontracts, etc. that perform PBMC isolation and cryopreservation.

2.2. This procedure does not describe the biospecimen collecting process. The biospecimen collecting process is dictated by the institute’s protocol.

3. REFERENCES

3.1. VIC_GL_002: Shipping SARS-CoV-2 Associated Specimens to the FNL Central Repository (NCI SeroNet Guidance Document)

3.2. VIC_GL_003: Key Entity Identifier Assignment (NCI SeroNet Guidance Document)

4. RESPONSIBILITIES

4.1. It is the responsibility of the institute performing the PBMC isolation and cryopreservation to:

4.1.1. Perform PBMC isolation and cryopreservation using the indicated reagents, materials, equipment and process parameters in this guidance document.

4.1.2. Ship the PBMCs to the FNL Central Repository following “VIC_GL_002: Shipping SARS-CoV-2 Associated Specimens to the FNL Central Repository (NCI SeroNet Guidance Document).”

4.2. It is the responsibility of the Vaccine, Immunity and Cancer Program (VIC) to:

4.2.1. Generate, review and approve the PBMC isolation and cryopreservation process guidance document.

4.2.2. Distribute the most current version of this guidance document to each institute associated with SeroNet.

5. DEFINITIONS

5.1. Acid Citrate Dextrose (ACD)

This document contains confidential and proprietary information. Do not copy or distribute without prior, written permission.

Effective Date: 24Nov20 Uncontrolled Print Copy For Reference Use Only

5.2. Biospecimen – a sample of biological material, such as urine, whole blood, blood components, tissue, cells, DNA, RNA, and protein.

5.3. Peripheral Blood Mononuclear Cell (PBMC) – any peripheral cell having a round nucleus; consists of lymphocytes (T cells, B cells, NK cells) and monocytes.

5.4. Severe Acute Respiratory Syndrome Coronavirus 2 (SARS-CoV-2)

6. REAGENTS, MATERIALS AND EQUIPMENT

6.1. Reagents

6.1.1. Dulbecco's Phosphate-Buffered Saline (DPBS), $Ca^{2}+$ and $Mg^{2}+$ free (Life Technologies, Cat # 14190-136 or equivalent)

6.1.2. Ficoll-Hypaque, density of 1.077 g/mL (Amersham Pharmacia Biotech, Cat # 17-1440-02)

6.1.3. RPMI-1640, No L-glutamine (Gibco, Cat # 21870076)

6.1.4. 200 mM L-glutamine (Gibco, Cat # 25030081)

6.1.5. 1M Hepes (Gibco, Cat # 15630-080)

6.1.6. Penicillin/Streptomycin (Sigma, Cat # P-0781)

6.1.7. Dimethyl Sulfoxide (DMSO), Cell Culture Grade (Sigma, Cat # D-2650)

1.1.1. Fetal Bovine Serum (FBS), Heat-Inactivated (Hyclone, Cat # SH30070.03HI)

6.1.8. Vital Stain Dye (e.g., Trypan Blue)

6.2. Consumables

Note: Consumables requiring approval for use as "equivalent" by the NCI SeroNet are indicated with an Asterisk (*).

6.2.1. 50 mL Polypropylene Centrifuge Tubes (Falcon, Cat # 352098 or equivalent)

6.2.2. 2 mL Cryovials (Fisher Scientific, Cat # 12-565-163N or equivalent*)

6.2.3. 15 mL Conical Tube (Falcon, Cat # 352097 or equivalent)

6.2.4. Serological Pipets, various sizes

6.2.5. Pipette Tips, various sizes

6.2.6. Wet Ice

This document contains confidential and proprietary information. Do not copy or distribute without prior, written permission.
Effective Date: 24Nov20 Uncontrolled Print Copy For Reference Use Only

6.2.7. Media Storage Bottle, various sizes

6.2.8. Labels that can withstand temperatures ≤ -80°C

6.2.8.1. Example: Brady Label (Anthony-Lee Associates, Cat # THT-133-461-SLIT)

6.2.9. BD vacutainer ACD tubes (Thomas Scientific, Cat # 9670A08 or equivalent*)

6.2.10. 2-inch box and 81 slot-grid

6.3. Equipment

6.3.1. Class II Biosafety Cabinet (BSC)

6.3.2. Benchtop Centrifuge

6.3.3. Hemocytometer

6.3.4. Inverted Microscope

6.3.5. Micropipettor

6.3.6. Automated Serological Pipet

6.3.7. Controlled-Rate Freezer

6.3.8. Liquid Nitrogen (LN_2)

6.3.9. Liquid Nitrogen (LN_2) Storage Freezer

6.3.10. 2-8°C Refrigerator

7. HEALTH AND SAFETY CONSIDERATIONS

Note: Each institute's Environment, Health, and Safety department will provide definitive measures for safety when processing human biospecimens as these considerations are provided only as a guideline.

7.1. Proper safety precautions should be taken while working in a laboratory setting. This includes, but is not limited to, proper protective equipment such as lab coats, safety glasses, closed-toe shoes, and non-latex gloves.

7.2. If SARS-CoV-2 positive samples are being processed, additional protective equipment is worn such as double layer of non-latex gloves and disposable arm sleeves.

7.3. A face mask is part of the standard personal protective equipment for the laboratory during the SARS-CoV-2 pandemic.

This document contains confidential and proprietary information. Do not copy or distribute without prior, written permission.

7.4. Follow the institute governed Biosafety Level 2 (BSL-2) requirements for handling and processing human biospecimens.

7.5. All human biospecimen processing work is performed inside of a Class II BSC.

7.6. Refer to the respective Safety Data Sheet (SDS) when working with any chemicals.

7.7. Refer to the institute’s processes for disposing of biohazardous and chemical waste.

8. PROCEDURE PRINCIPLES

8.1. Refer to “VIC_GL_003: Key Entity Identifier Assignment (NCI SeroNet Guidance Document)” for process of assigning IDs to biospecimens and biospecimen aliquots.

8.2. Image of form “VIC_LAB_001.01, PBMC Isolation and Cryopreservation Form” is attached for institute’s reference. The minimum information requiring documentation during the performance of this process is included in this form. See Attachment 1.

8.3. Image of form “VIC_LAB_001.02, PBMC Biospecimen Collection Form” is attached for institute’s reference. The minimum information requiring documentation during the performance of the blood biospecimen collection for PBMC isolation and cryopreservation is included in this form. See Attachment 2.

8.4. Phlebotomist should collect blood in ACD tubes.

8.5. It is preferred that all equipment used in this process is maintained, at minimum, per the equipment manufacturer’s recommendations.

8.6. It is preferred that all Micropipettors, Laboratory Freezers and Refrigerators, Benchtop Centrifuges, and Automated Cell Counters used in this process be calibrated by a vendor or other qualified party.

8.7. It is preferred that all Laboratory Freezers and Refrigerators used in this process be monitored for temperature by a temperature monitoring system.

8.8. All reagent preparation and human biospecimen handling are performed in a Class II Biosafety Cabinet (BSC) except for centrifugation, freezing cycle and storage.

9. REAGENT PREPARATION

9.1. RPMI-1640 Complete Media + 40% FBS

9.1.1. Combine reagents into appropriately sized media storage bottle. See Table 1 for preparation of 1000 mL; preparation can be scaled up or down as needed.

This document contains confidential and proprietary information. Do not copy or distribute without prior, written permission.

Table 1: RPMI-1640 Complete Media + 40% FBS Preparation (1000 mL)

Reagent	Volume (mL)
RPMI-1640, No L-Glutamine	570
Fetal Bovine Serum, Heat-Inactivated	400
200 mM L-Glutamine	10
1M Hepes	10
Penicillin/Streptomycin	10
**Total**	**1000**

9.1.2. Mix well by inversion.

9.1.3. Label reagent with Reagent Name, Lot Number/Tracking Number, preparation date, expiration date, storage condition and initials.

9.1.4. RPMI-1640 Complete Media + 40% FBS may be stored at 2-8°C for up to two weeks.

9.2. RPMI-1640 Complete Media + 15% DMSO

9.2.1. Prepare reagent day of use.

9.2.2. Combine reagents into appropriately sized media storage bottle. See Table 2 for preparation of 100 mL; preparation can be scaled up or down as needed.

Table 2: RPMI-1640 Complete Media + 15% DMSO Preparation (100 mL)

Reagent	Volume (mL)
RPMI-1640, No L-Glutamine	82
DMSO, Cell Culture Grade	15
200 mM L-Glutamine	1.0
1M Hepes	1.0
Penicillin/Streptomycin	1.0
**Total**	**100**

9.2.3. Mix well by inversion.

9.2.4. Label reagent with Reagent Name, Lot Number/Tracking Number, preparation date and initials.

9.2.5. Store reagent at 2-8°C or on wet ice until used.

This document contains confidential and proprietary information. Do not copy or distribute without prior, written permission.

9.2.6. Do not retain remaining reagent after processing, discard according to the organization's chemical disposal process.

10. PBMC ISOLATION

Note: Maximum allowable time from blood collection (processing of PBMC) to LN_2 storage is 8 hours.

10.1. Upon receipt of blood biospecimen, observe and record the total volume of blood biospecimen collected on form VIC_LAB_001.01.

10.2. Using a 50 mL polypropylene tube or appropriately sized sterile storage bottle/flask dilute the blood biospecimen with an equal volume of DPBS.

10.3. Label 50 mL or 15 mL conical tubes with sample identification number (ID).

10.4. Dispense 15 mL of Ficoll-Hypaque into labeled 50 mL conical tubes, or if using 15 mL conical tubes, dispense 4 mL of Ficoll-Hypaque into labeled tubes.

10.5. Carefully overlay diluted blood from step 10.2 onto the Ficoll-Hypaque from step 10.4.

10.5.1. When using 50 mL conical tube, the maximum volume is not to exceed 45 mL.

10.5.2. When using 15 mL conical tube, the maximum volume is not to exceed 13.5 mL. See Figure 1.

10.6. Centrifuge the samples for 20 minutes at 1000 x g at 20°C with the centrifuge brake turned off.

10.7. Using a transfer pipette or serological pipette, remove the PBMC layer and transfer to a single clean 50 mL centrifuge tube labeled with sample ID. See Figure 1.

Figure 1: Image of Blood Overlay and Layers Post Centrifugation

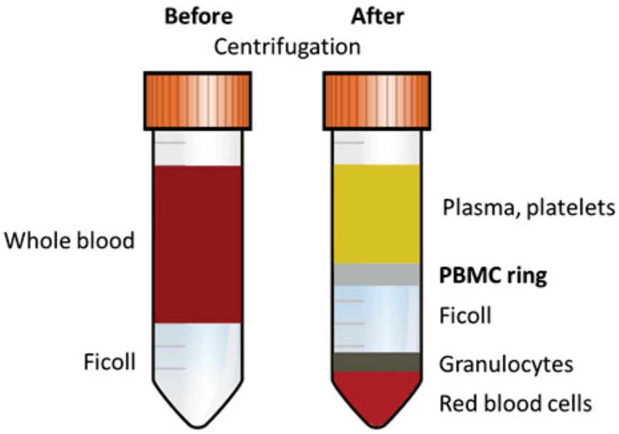

10.8. Wash the PBMCs by quantum satis (q.s.) to 45 mL with DPBS, then centrifuge for 10 minutes at 470 x g at 20°C with the brake on.

10.9. Decant the supernatant.

10.10. Wash the PBMC pellet one additional time with 45 mL DPBS. Centrifuge for 10 minutes at 300 x g at 20°C with brake on.

10.11. Decant the supernatant.

10.12. Resuspend cells in cold RPMI-1640 Complete Media + 40% FBS (1 mL).

10.13. Perform a cell count using hemocytometer. See Attachment 3 for cell counting using a hemocytometer.

Note: If the institute has an Automated Cell Counter, the institute can perform a second count on the cell counter as For Information Only (FIO).

10.14. Record the hemocytometer cell count and calculate viability (live cells ÷ total cells x 100%). Only proceed with cryopreservation if viability is greater than 80%.

11. CRYOPRESERVATION

Note: It is very important at this point that cells, media, and tubes are kept cold on wet ice.

11.1. Label 2 mL cryovials using Attachment 4. Refer to VIC_GL_003 for biospecimen aliquot ID assignment process. **Use Deidentified Biospecimen Aliquot ID Only**.

11.2. Adjust cell concentration to be 20 x 10^6 cells/mL using RPMI-1640 Complete + 40% FBS.

11.3. Add dropwise an equal volume of cold RPMI-1640 Complete + 15% DMSO giving a final freezing solution of RPMI-1640 Complete containing 20% FBS and 7.5% DMSO. Resuspend cells gently.

11.4. Transfer 1.0 mL of the cell suspension (well suspended) using a pipette with a 1000 µL tip into each of the pre-chilled 2 mL cryovials.

Note: Gently mix cells by inversion after 2-3 minutes has passed for cells to settle, before transferring the cells.

11.5. Maintain the cells on wet ice until all samples are ready for transfer to the controlled-rate freezer. Processing should be performed quickly due to the recognized toxicity of DMSO.

11.6. Controlled-Rate Freezer

11.6.1. See Attachment 5 for the controlled-rate freezer program.

11.6.2. Prechill the controlled-rate freezer to a starting temperature of 4°C.

Verify current version prior to use. Use of a superseded or obsolete document is prohibited.

This document contains confidential and proprietary information. Do not copy or distribute without prior, written permission.

Effective Date: 24Nov20 Uncontrolled Print Copy For Reference Use Only

11.6.3. Prepare one control vial to regulate the controlled- rate freezer. Use the same volume and concentrations as the final freezing solution (i.e., 0.5 mL of RPMI-1640 Complete + 40% FBS plus 0.5 mL RPMI-1640 Complete + 15% DMSO).

11.6.4. Transfer cryovials immediately to the controlled-rate freezer.

11.6.5. Place the PBMC biospecimen aliquot vials and the control vial into the freezing chamber.

11.6.6. Place the freezer thermocouple into the control vial. Allow the control vial temperature and the chamber temperature to equilibrate to 4°C.

11.6.7. Begin the programmed, controlled-rate freeze.

11.6.8. At the conclusion of the freeze cycle, the cryovials will have reached -90°C and are transferred directly to freeze boxes for liquid nitrogen storage.

11.6.9. Check the freezing report to assure appropriate controlled-rate freezing. Make note on record (form VIC_LAB_001.01) if the parameters were not met. Retain controlled-rate freezer report print out with record.

11.6.10. Record the number of vials frozen. Attached is an example vial label to the record (VIC_LAB_001.01).

11.6.11. If there were problems encountered during PBMC biospecimen processing, note these on the record (form VIC_LAB_001.01). Record any problems with freezing procedure.

11.7. Ship PBMCs in LN_2 shipper to the FNL Central Repository following VIC_GL_002.

12. ATTACHMENTS

12.1. Attachment 1: VIC_LAB_001.01, PBMC Isolation and Cryopreservation Form

12.2. Attachment 2: VIC_LAB_001.02, PBMC Biospecimen Collection Form

12.3. Attachment 3: Counting Cells with a Hemocytometer

12.4. Attachment 4: Vial Label and Box / Rack Label

12.5. Attachment 5: Controlled-Rate Freezer Program Parameters

13. REVISION HISTORY

Version	Change	Reason
1.0	New guidance document for isolation and cryopreservation of PBMC by SeroNet organizations.	Currently no procedure; new initiative requiring communication of expectations.

This document contains confidential and proprietary information. Do not copy or distribute without prior, written permission.

2.0	1. Replaced “sample” and “specimen” with “biospecimen” throughout the document.	1. Consistency between documents and database verbiage.
	2. Minor formatting and grammatical changes throughout the document.	2. Clarification, ease of use.
	3. Added VIC_GL_003 to References section.	3. Referred in the body of the procedure.
	4. Added Biospecimen and SARS-CoV-2 to Definitions section.	4. Clarification.
	5. Added Asterix to consumables requiring approval by SeroNet for use as equivalent.	5. Reflect current practice.
	6. Removed Automated cell counter, -80C, -20C and cell freeze device from equipment section.	6. Reflect current practice.
	7. Added SARS-CoV-2 pandemic health and safety guidelines to Health and Safety Considerations section.	7. Clarification.
	8. Added reference to VIC_GL_003 and new form VIC_LAB_001.02 to Procedure Principles section.	8. Clarification.
	9. Reworded equipment requirements to be “preferred” in the Procedure Principles section.	9. Clarification.
	10. Added option to do cell count using Automated Cell Counter; hemocytometer cell count is required.	10. Clarification; reflect current practice.
	11. Removed use of cell device as option for cell freeze.	11. Reflect current practice.
	12. New form VIC_LAB_001.02 for collection of PBMC biospecimen.	12. Ease of use.
	13. Revised form VIC_LAB_001.01 to have biospecimen receipt, removed automated cell counter and -80C/-20C freezers, removed N/A boxes for required equipment.	13. Ease of use, reflect current practice.

This document contains confidential and proprietary information. Do not copy or distribute without prior, written permission.

Attachment 1: VIC_LAB_001.01, PBMC Isolation and Cryopreservation Form

Frederick National Laboratory for Cancer Research — sponsored by the National Cancer Institute		Vaccine, Immunity and Cancer Program Standard Operating Procedure Form	
**Form Title:** PBMC Isolation and Cryopreservation Form			
**Document ID: VIC_LAB_001.01**		Version:	2.0
Associated SOP: VIC_LAB_001		Effective Date:	
Supersedes:	1.0	**Page 1 of 5**	

Biospecimen Receipt

PBMC Biospecimen Processing Laboratory Name:					
Biospecimen Number	Deidentified Biospecimen ID	Volume (mL	Date Received	Time Received (24H)	Initials
1					
2					
3					
4					
5					

Equipment

Equipment Name	Equipment ID	Calibration Due Date
BSC		
Centrifuge		
Pipette		
☐ N/A Pipette		
☐ N/A Pipette		
☐ N/A 2-8°C Refrigerator		
Microscope		
Hemocytometer		
☐ N/A Automated Cell Counter		
Controlled-Rate Freezer		
LN_2 Storage Freezer		

Verify current version prior to use. Use of a superseded or obsolete document is prohibited.

This document contains confidential and proprietary information. Do not copy or distribute without prior, written permission.

Verify current version prior to use. Use of a superseded or obsolete document is prohibited.

This document contains confidential and proprietary information. Do not copy or distribute without prior, written permission.

Frederick National Laboratory for Cancer Research
sponsored by the National Cancer Institute

Vaccine, Immunity and Cancer Program
Standard Operating Procedure
Form

**Form Title:** PBMC Isolation and Cryopreservation Form			
**Document ID: VIC_LAB_001.01**		Version:	2.0
Associated SOP: VIC_LAB_001		Effective Date:	
Supersedes:	1.0	**Page 2 of 5**	

Reagents

Reagent Name	Catalog Number	Lot Number	Expiration Date
DPBS			
Ficoll-Hypaque			
RPMI-1640, no L-Glutamine			
Fetal Bovine Serum			
200 mM L-Glutamine			
1M Hepes			
Penicillin/Streptomycin			
DMSO, Cell Culture Grade			
☐ N/A Vital Stain Dye (e.g. Trypan Blue)			

Consumables

Consumable Name	Catalog Number	Lot Number	Expiration Date
50 mL Polypropylene Tube			
☐ N/A 15 mL Conical Tube			
☐ N/A 2 mL Cryovial			
☐ N/A Cryovial Label		☐ N/A	☐ N/A
☐ N/A			

PBMC Isolation

Processing Steps

(√)	Process Step
	Dilute blood biospecimen with equal volume of DPBS.
	Dispense Ficoll-Hypaque into conical tubes.
	Overlay diluted blood biospecimen onto Ficoll-Hypaque.
	Centrifuge for 20 min, 1000 x g, 20°C, brake OFF.
	Remove PBMC layer and transfer to 50 mL conical tube. QS to 45 mL with DPBS.
	Centrifuge for 10 min, 470 x g, 20°C, brake ON.
	Decant supernatant. Add 45 mL DPBS.
	Centrifuge for 10 min, 300 x g, 20°C, brake ON.
	Decant supernatant. Resuspend in COLD RPMI-1640 Complete + 40% FBS

Verify current version prior to use. Use of a superseded or obsolete document is prohibited.

This document contains confidential and proprietary information. Do not copy or distribute without prior, written permission.

Verify current version prior to use. Use of a superseded or obsolete document is prohibited.

This document contains confidential and proprietary information. Do not copy or distribute without prior, written permission.

Frederick National Laboratory for Cancer Research
sponsored by the National Cancer Institute

Vaccine, Immunity and Cancer Program
Standard Operating Procedure
Form

**Form Title:** PBMC Isolation and Cryopreservation Form			
**Document ID: VIC_LAB_001.01**		Version:	2.0
Associated SOP: VIC_LAB_001		Effective Date:	
Supersedes:	1.0	**Page 3 of 5**	

Reagent Preparation

RPMI-1640 Complete + 40% FBS	
Reagent	Volume (mL)
RPMI-1640, no L-Glutamine	
Fetal Bovine Serum	
200 mM L-Glutamine	
1M Hepes	
Penicillin/Streptomycin	

Cell Count – Hemocytometer (Required)

Biospecimen Number	Live Cells	Dead Cells	Total Cells	% Viability
1				
2				
3				
4				
5				

Cell Count – Automated Cell Counter (For Information Only)

Biospecimen Number	Live Cells	Dead Cells	Total Cells	% Viability
1				
2				
3				
4				
5				

Verify current version prior to use. Use of a superseded or obsolete document is prohibited.

This document contains confidential and proprietary information. Do not copy or distribute without prior, written permission.

Verify current version prior to use. Use of a superseded or obsolete document is prohibited.

This document contains confidential and proprietary information. Do not copy or distribute without prior, written permission.

Frederick National Laboratory for Cancer Research
sponsored by the National Cancer Institute

Vaccine, Immunity and Cancer Program
Standard Operating Procedure
Form

**Form Title:** PBMC Isolation and Cryopreservation Form			
**Document ID: VIC_LAB_001.01**		Version:	2.0
Associated SOP: VIC_LAB_001		Effective Date:	
Supersedes:	1.0	**Page 4 of 5**	

PBMC Cryopreservation

Reagent Preparation

RPMI-1640 Complete + 15% DMSO (Freeze Media)	
Reagent	Volume (mL)
RPMI-1640, no L-Glutamine	
DMSO, Cell Culture Grade	
200 mM L-Glutamine	
1M Hepes	
Penicillin/Streptomycin	

Processing Steps

(√)	Process Step
	Label 2 mL cryovials. Chill at 2-8°C.
	Adjust cell concentration to be 20 x 10^6 cells/mL using RPMI-1640 Complete + 40% FBS.
	Add dropwise an equal volume of cold RPMI-1640 Complete + 15% DMSO. Gently resuspend cells.
	Transfer 1.0 mL of the cell suspension into each labeled 2 mL cryovial.
	Freeze PBMCs using Controlled-Rate Freezer.
	Store PBMCs in LN_2 Freezer.

Controlled-Rate Freezer, see attached printout

Biospecimen Number	Date / Time (24H) Blood Biospecimen Collected	Date / Time (24H) PBMC Biospecimen Stored in LN_2 Freezer	Initials
1			
2			
3			
4			
5			

Verify current version prior to use. Use of a superseded or obsolete document is prohibited.

This document contains confidential and proprietary information. Do not copy or distribute without prior, written permission.

Verify current version prior to use. Use of a superseded or obsolete document is prohibited.

This document contains confidential and proprietary information. Do not copy or distribute without prior, written permission.

Frederick National Laboratory for Cancer Research — sponsored by the National Cancer Institute		Vaccine, Immunity and Cancer Program Standard Operating Procedure Form	
**Form Title:** PBMC Isolation and Cryopreservation Form			
**Document ID: VIC_LAB_001.01**		Version:	2.0
Associated SOP: VIC_LAB_001		Effective Date:	
Supersedes:	1.0	**Page 5 of 5**	

Biospecimen Number	Number of Cryovials Frozen	Example Label
1		
2		
3		
4		
5		

Comments: ☐ N/A

Performed by/date:	
Reviewed by/date:	

Verify current version prior to use. Use of a superseded or obsolete document is prohibited.

This document contains confidential and proprietary information. Do not copy or distribute without prior, written permission.

Verify current version prior to use. Use of a superseded or obsolete document is prohibited.

This document contains confidential and proprietary information. Do not copy or distribute without prior, written permission.

Attachment 2: VIC_LAB_001.02, PBMC Biospecimen Collection Form

Frederick National Laboratory for Cancer Research sponsored by the National Cancer Institute		Vaccine, Immunity and Cancer Program Standard Operating Procedure Form	
**Form Title:** PBMC Biospecimen Collection Form			
**Document ID: VIC_LAB_001.02**		Version:	2.0
Associated SOP: VIC_LAB_001		Effective Date:	
Supersedes:	1.0	**Page 1 of 1**	

Deidentified Biospecimen ID:					
Biospecimen Group:	☐ Positive ☐ Negative ☐ Serosurveillance				
Section I. Vacutainer Collection Tube					
Type:	☐ ACD ☐ Other				
Catalog No.:					
Lot No.:	☐ N/A				
Exp. Date:					
Section II. Blood Biospecimen Collection					
Name of Clinic/Company:					
Date:		Time: (24 Hr)		Initials:	

Reviewed by/date: ______________________________

Verify current version prior to use. Use of a superseded or obsolete document is prohibited.

This document contains confidential and proprietary information. Do not copy or distribute without prior, written permission.

Attachment 3: Counting Cells with a Hemocytometer

- Count cells with a hemocytometer using vital stain dye (Trypan blue).
    - Note: May start with a 1:2 dilution (equal volumes of vital stain dye and cells). However, the dilution may need to change, so the total cell count of quadrants A, B, C, and D is ~80-200 cells.
- Add 10 µL of vital stain dye/cell mixture to the hemocytometer.
- Count cells in quadrants A, B, C, and D (refer to diagram below). <u>Only count cells that fall on two of the four outer edges of each of the four quadrants,</u> as defined by the red lines in the diagram below.

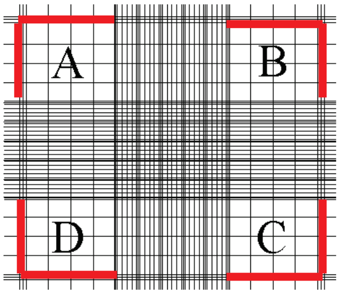

- Record the number of live cells (blue negative), dead cells (blue positive) and total cells (live cells + dead cells).
- To calculate cell concentration, use the following formula:

  (Total cells counted ÷ Number of quadrants counted) x Dilution Factor x 10,000

  For example, a sample that was diluted 1:2 had 100 live cells counted in four quadrants.
  (100 ÷ 4) x 2 x 10,000 = 500,000 cells/mL

- To calculate cell viability, use the following formula:
  (Live cells ÷ Total cells) x 100%

  For example, a sample has 75 live cells and 50 dead cells.
  Total cells = 75 live + 50 dead = 125
  Viability = (75 ÷ 125) x 100% = 60%

Attachment 4: Vial Label and Box / Rack Label

Vial Label

Biospecimen Aliquot ID
PBMC
Volume
Cell Concentration

Barcode:	Barcode linked to Biospecimen Aliquot ID
Line 1:	Deidentified Biospecimen Aliquot ID
Line 2:	PBMC
Line 3:	Volume (mL)
Line 4:	Final Cell Concentration (x 10^6 cells/mL)

Example Label:

A1_123456_123_1
PBMC
1.0 mL
10 x 10^6 cells/mL

Box / Rack Label

Study: ?????? / ??????
Biospecimen Type: ?????
Date: DDMMMYY
Shipping ID: XXXXXXX
Box ? **of** ?

Line 1:	SeroNet
Line 2:	PBMC
Line 3:	Date in DDMMMYY format
Line 4:	Shipping ID
Line 5:	Box Number

Example Label:

Study: SeroNet
Biospecimen Type: PBMC
Date: 01Jan20
Shipping ID: XXXXXXX
Box 1 **of** 10

Box Label Placement

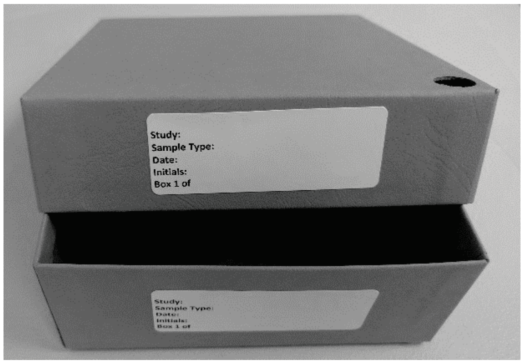

Attachment 5: Controlled-Rate Freezer Program Parameters

Step No.	Rate (°C/min)	End Temp (°C)	Hold (m s)	Trigger
1			5m 0s	Chamber
2	-1.00	-4.00		Chamber
3	-25.00	-50.00		Chamber
4	10.00	-20.00		Chamber
5	-1.00	-40.00		Chamber
6	-5.00	-90.00		Chamber
7			0m 0s	Chamber

Template for inclusion of SARS-CoV-2 vaccine booster in surveillance studies

Purpose:
Provide a common study template that could be used within SeroNet and beyond for the serosurveillance of individuals, including cancer/immunocompromised patients, receiving a SARS-CoV-2 vaccine booster. The template could be applicable to prospective interventional trials when investigators have access to vaccines for experimental use, or for observational studies where individuals receive vaccines as they become available in the community.

Dependent upon the needs of the study, this template provides guidance about recommended timepoints and sample types to collect post-boost for longitudinal evaluation of the immune response and to facilitate meta-analysis of the data.

Population can include:
- Immunocompromised individuals due to a cancer diagnosis or the effects from their treatment
  - Newly diagnosed cancer patients
  - Patients currently undergoing treatment for cancer, including chemotherapy, radiation therapy, immunotherapy, targeted therapy, or combinations of therapies
- Individuals with a history of cancer/cancer survivors
- Individuals with autoimmune diseases that are immunosuppressed
- Other immunocompromised individuals
- General population

This document is intended to be agnostic as to which booster individuals receive and will provide recommendations for:
- Specimen selection and collection
  - Timepoints for boost and collection
  - Specimen types
  - Specimen collection protocols
  - Key determinants to be considered
- Assays
  - Type
  - Benchmarking/standardizing
  - Standard protocols where available
- Data collection and common data elements (CDEs)

Design Considerations:
- Vaccine responses can be influenced by a number of factors and conditions
  - Age, prior SARS-CoV-2 infection, BMI, other infections, and co-morbidities
  - Time since first SARS-CoV-2 vaccine regimen, and the interval between the initial prime/boost series.
  - Cancer and autoimmune disease and their treatments could affect immunocompetence and therefore vaccination/boost responses.
  - Individuals with cancer are a complex and extremely diverse population and there are a multitude of considerations, including approaches to capturing the appropriate clinical information regarding an individual's cancer type, subtype, stage, treatments/regimen

(chemotherapy, radiation therapy, immunotherapy, surgery), time since diagnosis, timing of therapy, etc.
    - Individuals with autoimmune diseases could experience flareups or other adverse reactions following vaccination.
- Recommendations for when specimens should be collected for optimal tracking of the vaccine boost induced immune response.
    - Inclusion of additional sampling timepoints following a documented infection (positive PCR) after boost.
    - Investigators should aim to collect timepoints within a narrow window, not more than 10-days from the scheduled timepoint to facilitate meta-analysis.
- Detailed collection of demographics and non-cancer related clinical data such as performance status, chronic diseases and specific therapies, co-morbidities, tobacco history (particularly lung cancer), alcohol history, other medications, etc.
- Collection of quality-of-life considerations and patient reported outcomes.

Scientific and Clinical Questions

- Homologous versus heterologous boost strategies. It is important to capture information about the initial vaccine type and regimen as vaccines may have a different mechanism of action that could impact the immune response.
- Changes in incidence of adverse events (vaccine or cancer related).

Timeline and specimen collection

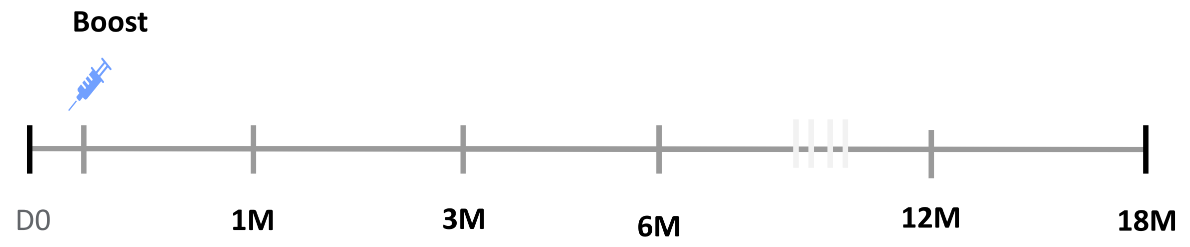

Figure 1. Recommended timepoints for the collection of biospecimens following SARS-CoV-2 booster vaccination. The timepoints indicate the recommended minimal timepoints for specimen collection to measure the immune response post-boost.

Figure 1 shows the minimum recommended sample collection timepoints for studying the effect of a vaccine boost. Baseline serology assessment is critical for testing the ability of a (homologous or heterologous) vaccine boost to illicit a measurable serology response. The D0 timepoint is intended to establish a baseline and samples should be collected prior to the boost. This sample collection can occur on the same day as the vaccination, or up to one week before a planned boost. For long-term immune assessment it is recommended to include a timepoint at 12- and 18-months post-boost. For near-term assessment of whether a vaccine boost is successful in inducing an immune response an interim analysis at 3 months post-boost is recommended.

Samples should be collected as close to the indicated timepoint as possible and within a 10-day window. It is also recommended to collect a nasal swab at the time other specimens are collected for monitoring for breakthrough infection and viral shedding.

Table 1. Recommended collection timepoints per specimen type. PB denotes post-boost timing. The timepoints with an X denote the minimum recommended specimen collection per timepoint. Other sample types within the table are recommended for additional collections based on study aims.

Sample Type	D0 (Baseline)	1M (PB)	3M (PB)	6M (PB)	12M (PB)	18M (PB)
Serum	X	X	X	X	X	X
Plasma						
PBMC	X	X	X	X	X	X
DBS						
Saliva						
Nasal Swab	X	X	X	X	X	X

Table 2. Minimum recommended assays per timepoint following vaccination. X denotes the minimum recommended assays per timepoint. Other sample collection timepoints within the table are recommended for additional assays depending on study aims.

Sample Type	D0 (Baseline)	1M (PB)	3M (PB)	6M (PB)	12M (PB)	18M (PB)
Serology (IgG LBA)	X	X	X	X	X	X
Neutralization Assay	X	X	X	X	X	X
T Cell Assay	X	X	X	X	X	X

Sample Preparation and Assay Types

It is recommended to harmonize study collection protocols and to use assays that have been well characterized and validated. Below is a list of recommended assay types and protocols as available at the time of document completion. Sample collection/preparation protocols are dependent upon the assay type.

- Serum biospecimen processing
- Plasma biospecimen processing
- PBMC isolation and cryopreservation
- **Serology (LBA):** To distinguish natural infection from vaccine induced immunity we recommend performing an IgG LBA against both the Spike (natural infection and vaccine-mediated) and N (natural infection) proteins.
  - It is recommended to use an FDA EUA semi-quantitative or quantitative assay that is calibrated against the National SARS-CoV-2 Serology Standard and report all findings in international units (IUs).
- **Serology (neutralization assay):** To determine the presence and magnitude of functional, neutralizing SARS-CoV-2 antibodies as well as correlation with ligand binding assay results.
- **Immune cell assessments:** Disease related abnormalities and treatments for cancer and autoimmune disease can impact immune cell populations/ immunocompetence and potentially affect the response to vaccination. Therefore, especially a baseline immune assessment will be critical for the correlation of immune kinetics to immune status.
  - Assay types can include Complete Blood Count (CBC), flow-cytometry and Cutoff.
- **T cell assays:**
  - **ELISpot**: Quantitative assay that measures cytokines released from antigen stimulated T cells.

    - **Intracellular cytokine staining (ICS):** Flowcytometry-based assay that allows for simultaneous cellular phenotyping and single cell cytokine detection used to assess T cell responses.
    - **Activation Induced Marker (AIM) Assay:** Detection of antigen specific T cells.
- **SARS-CoV-2 diagnostic test**: PCR-based assay to detect viral RNA.
    - If a breakthrough infection is noted additional follow-up may be required

Common Data Elements

To facilitate metanalyses SeroNet recommends the use of a minimum set of common data elements (CDEs). Some data elements are recommended to be collected at each timepoint/encounter if feasible, others can be collected following consent at the beginning of the study. CDEs are roughly divided by:

- Demographics
- SARS-CoV-2 vaccine (and infection) history
- Clinical characteristics
- Patient reported outcomes
- Assay results
