# Supplementary material for: Mission, Organization, and Future Direction of the Serological Sciences Network for COVID-19 (SeroNet) Epidemiologic Cohort Studies
Source: Open Forum Infect Dis. 2022 Apr 27;9(6):ofac171. doi: 10.1093/ofid/ofac171 (PMC9129196; doi:10.1093/ofid/ofac171)
Supplement: ofac171_suppl_Supplementary_Appendix_B [file ofac171_suppl_supplementary_appendix_b.pdf]

Appendix B

Characteristics of SeroNet Research Projects

**Project Title:** Multiplexed in-solution serological test for SARS-CoV-2, human coronaviruses and other respiratory pathogens
**Grant number:** ASU CBC 21X089
**Primary contact:**<br><br>Joshua LaBaer, M.D., Ph. D.<br>Arizona State University<br>Address: 1001 S. McAllister Ave., Tempe, AZ, 85281<br>Phone number: 480-965-2805<br>Email: Joshua.Labaer@asu.edu
**Other collaborators:**<br><br>Name: Ji Qiu<br>Affiliation(s): Arizona State University<br>Email: Ji.Qiu@asu.edu<br><br>Name: Milene Peterson<br>Affiliation(s): Arizona State University<br>Email: mtavare1@asu.edu<br><br>Name: Femina Rauf<br>Affiliation(s): Arizona State University<br>Email: frauf@asu.edu<br><br>Name: Jin Park<br>Affiliation(s): Arizona State University<br>Email: Jin.Park.1@asu.edu<br><br>Name: Vel Murugan<br>Affiliation(s): Arizona State University<br>Email: Velmurugan@asu.edu<br><br>Name: Lusheng Song<br>Affiliation(s): Arizona State University<br>Email: lsong14@asu.edu
**Institution(s):** Arizona State University
**Project Aims:**<br><br>• We will acquire and conduct quality control assessments of critical reference samples.<br>• We will optimize and obtain EUA authorization for an ASU-developed multiplex in-solution quantitative serological assay with high specificity and sensitivity and high-throughput for SARS-CoV-2.<br>• We will offer clinical testing services to underserved communities across Arizona.<br>• We will develop assay for antibody responses to all 7 human coronaviruses and other common respiratory viruses.<br>• We will study post-vaccine serosurveillance in immunocompromised and/or immunosuppressed patients to understand seroconversion and the dynamics of serological responses after vaccination.<br>• We will study convalescent immunity in COVID-19 recovered patients to understand the dynamics of serological responses to SARS-CoV-2 infection.

Primary study design:

- (Post-vaccination serosurveillance) We will collect samples from patients and healthy controls prior to and after vaccination. We will assay anti-SARS-CoV-2 antibodies in serum samples and assess sero-conversion after vaccination and the dynamics of antibody decay over time.
- (Convalescent serosurveillance) We will collect samples from COVID-19 patients and assess antibody dynamics after infection.

Study population:

- (Post-vaccination serosurveillance) Immunocompromised or immunosuppressed HIV, cancer or transplant patients and immunocompetent controls
- (Convalescent serosurveillance) COVID-19 patients

Age/Sex/Race/Ethnicity:

- We do not exclude study subjects based on age, gender, race, or ethnicity.

Geography:

- Our study subjects will be recruited mainly in Arizona and New York.

Current/Target number enrolled:

Currently enrolled up to May 1, 2021: 100 participants, all with a pre-vaccine visit.

Planned total enrollment:

- (Post-vaccination serosurveillance)
  - 1125 Immunocompromised or immunosuppressed HIV, cancer, or transplant patients
  - 375 immunocompetent controls
- (Convalescent serosurveillance)
  - 500 COVID-19 patients

Data collection period:

3/2021 – 10/2025

Data collection intervals:

- (Post-vaccination serosurveillance) Samples will be collected at 6 time points: Pre-vaccination (When possible or as close to the completion of the vaccine series as possible), 1m, 3m, 6m, 12m and 24m post-vaccination
- (Convalescent serosurveillance) Samples will be collected at 6 time points: 30 days within a positive COVD-19 PCR test, 1m, 3m, 6m, 12m and 24 m after enrollment.

Data collection methods:

Data will be collected from patients' questionnaires and electronic health record data abstraction.

Data elements:

Demographics: Age, Gender, Race, Ethnicity
COVID-19 Vaccination History
- Dates of vaccine and manufacture

COVID-19 Infection History

- Dates, severity, hospitalization

COVID-19 symptoms when applicable

- Fever, chills, muscle aches, runny nose, soar throat, loss of smell, loss of taste, cough, headache, difficulty breathing, shortness of breath, chest pain, nausea or vomiting, diarrhea, other

COVID-19 symptoms not resolved, (same list as above).

COVID-19 Complications

- Acute respiratory failure, pneumonia, acute respiratory disease, acute liver injury, acute cardiac injury, secondary infection, acute kidney injury, septic shock, disseminated intravascular coagulation, blood clots, chronic fatigue, rhabdomyolysis, multisystem inflammatory syndrome in children.

Comorbidities:

- Diabetes mellitus Type 1, Diabetes mellitus Type 2, Obesity, Hypertension, Coronary artery disease, Congestive Heart failure, Kidney disease, Liver disease, Asthma, Other chronic respiratory disease (COPD, Emphysema), Pulmonary fibrosis, Chronic oxygen needs, Epilepsy, Multiple sclerosis, Dementia, Alzheimer's disease, Other neurological disease, Other cancer, Autoimmune disease

Infection History;

- Hep-C, Hep-B, Hep-A, Tuberculosis, MRSA, Chicken pox, Positive PPD, West Nile, Measles, Rheumatic Fever, Meningitis, Rubella, Sexually transmitted disease – type

Vaccine History

- Influenza, Hep-B, Tetanus, Shingles, Pneumococcal, Measles, Mumps, Rubella, Varicella, Bacillus Calmette-Guerin

Social History

- Smoking history,
- Alcohol History
- Average number of drinks in the past 12 months, Max number of drinks in 24 hrs. in the past year, Average number of drinks in a day, Consumption of more than 5 drinks (male) 4 drinks (female) in a day in the last year
- Use or misuse of recreational or other drugs.
-

Specimens collected: Serum, PBMC, anterior nasal swab and saliva

Serology/other assays:

We will use an innovative multiplexed in solution protein array (MISPA) to assay antibodies against the immunodominant antigens from SARS-CoV-2, other 6 human coronaviruses, and other respiratory pathogens.

Project Title: Pre-exposure Immunologic Health and Linkages to SARS-COV2 Serologic Responses, Endothelial Cell Resilience, and Cardiovascular Complications: Defining the mechanistic basis of high risk endotypes" (Cardio-COVID)

Grant number: U01CA260513-01

Primary contact:

David A. Zidar, MD, PhD
Case Western Reserve School of Medicine, Louis Stokes Cleveland VAMC, University Hospitals of Cleveland
Address:10701 East Blvd, Cleveland; Cleveland, Ohio 44106
Phone number:216-791-3800
Email: daz21@case.edu

Other collaborators:

Name: Timothy Chan, MD PhD
Affiliation(s): Cleveland Clinic/Lerner School of Medicine
Email:chant2@ccf.org

Name: Christopher L. King MD PhD
Affiliation(s): Case Western Reserve School of Medicine, Louis Stokes Cleveland VAMC, University Hospitals of Cleveland
Email: cxk21@case.edu

Institution(s): Case Western Reserve School of Medicine; The Lerner School of Medicine

Project Aims:
To determine the mechanistic relationships between pre-existing lymphopenia and anisocytosis (subclinical immunologic dysfunction), protective serologic responses, and cardiopulmonary resilience in response to COVID.

Primary study design:
Retrospective analysis of COVID-19 patients who have had an outpatient complete cell count/leukocyte differential (CBC/diff) within 1 year to 1 month prior to COVID-19.

Study population:
United States Veterans with PCR-test confirmed COVID-19 between March 2019 through 12/31/2020.

Age/Sex/Race/Ethnicity:
N=146,449; 90% male; 67% white; 23% black; 10% Latino; mean (SD) age: 61.1 (16.5)

Geography: National

Current/Target number enrolled:
N=146,449 will be the final for this analysis

Data collection period:
March 2019 through 12/31/2020

Data collection methods:
EHR extraction

Data elements:

Mean Age, years (SD)
Male Sex
Race/Ethnicity
Non-Hispanic White
Non-Hispanic Black
Hispanic
Race Multi/Other/Declined
Charlson Score
Systolic: mean(SD)
Diastolic: mean(SD)
Mean (SD) total cholesterol
Mean (SD) HDL
Mean (SD) LDL
DM
Smoking status: current
Smoking status: former
Smoking status: never
Prior Myocardial Infarction
Prior Heart Failure
Peripheral Vascular Disease
Stroke
Dementia
Pulmonary Disease
Renal Disease
Rheumatologic Disease
Peptic Ulcer Disease
Paralysis
Cancer
HIV
5-year ASCVD: mean(SD)
Platelet Count (k/uL) (
Absolute Basophil Count (uL)
Absolute Eosinophil Count (k/uL)
Mean Cell Hemoglobin Level (g/dL)
Absolute Lymphocyte Count (k/uL)
Absolute Neutrophil Count (k/uL)
Red Blood Cell Distribution Width (%)
Mean Corpuscular Volume (fL)
Absolute Monocyte Count (k/uL)

Specimens collected:

Specimens are being collected prospectively and stored for future use.

Serology/other assays:

Reactivity of serum from infected with SARS-COV-2 to the full-length S protein (aa 16-1230), the receptor binding domain (RBD aa 319-541) of the S1 protein, and N protein (aa 1-419) (left panel), and the ability of serum at different dilutions from COVID-19 individuals (N=8, red) and pre-COVID-19 (black) to block binding of RBD on beads to recombinant biotinylated ACE2 (at 0.1ug/ml) detected with streptavidin-PE at 1:500 dilution.

**Project Title:** Early Drivers of Humoral Immunity to SARS-CoV-2 Infections
**Grant number:** U01 CA260539-01
**Primary contact:**<br><br>Christopher L. King, MD, PhD, MPH<br>Case Western Reserve University and Veterans Affairs Medical Center<br>Address:10900 Euclid Avenue, Cleveland, OH<br>Phone number:216-269-7728<br>Email:cxk21@case.edu
**Other collaborators:**<br><br>Name: Jeffrey Jacobson, MD<br>Affiliation(s): Case Western Reserve University<br>Email: jxj573@case.edu<br><br>Name:Jon Karn, PhD<br>Affiliation(s): Case Western Reserve University<br>Email: jxk153@case.edu<br><br>Name:Adam Burgener, PhD<br>Affiliation(s): Case Western Reserve University<br>Email:
**Institution(s):** Case Western Reserve University
**Project Aims:**<br><br>1. To examine the early immune responses to CoV2 in close contacts of individuals diagnosed with COVID-19 and evaluate the humoral and cellular correlates of immunity.<br><br>2. To assess early innate immune responses in close contacts of individuals diagnosed with COVID-19 and assess their relationship with humoral immune responses and viremia<br><br>3. To examine early drivers of humoral immunity on the long-term durability of immunological memory and responses to vaccines.
**Primary study design:**<br><br>Household contact study
**Study population:**<br><br>Individuals exposed to people known to have Covid-19 and determine whether they become infected or not and mechanisms as to why they avoid infection
**Age/Sex/Race/Ethnicity:**<br><br>Adult and now amending to children age 12 and older. Open to all irrespective sex or ethnicity.
**Geography:** Northeast Ohio

Current/Target number enrolled:

Just started enrollment about 1 month ago. Very few so far. Planned to enroll ~200

Data collection period:

See below

Data collection intervals:

Peripheral blood along with nasopharyngeal swabs, and saliva will be sampled on days 0, 1, 3, 7, 10, 14, and 28 and every 6 months for up to 3 years. This will include whether participants have been vaccinated.

Data collection methods:

In person visits and diaries during time enrolment.

Data elements:

Age, sex, ethnicity, comorbidities, Covid-19 and other vaccination history, information on nature of contact with index case, assessment of symptoms with daily diaries.

Specimens collected:

Peripheral blood along with anterior nares swabs, nasopharyngeal swabs, tongue brushings and saliva

Serology/other assays:

Bead array assays to measure antibodies to S and N proteins and RBD in serum. From SeroNet we are including RBD variants. We use recombinant ACE2 binding inhibition (with test sera) to RBD beads as surrogate for neutralizing antibodies. We also measure antibody isotypes and avidity using chaotrophic methods. We evaluate S and N proteins and RBD- specific sIgA and IgG in saliva relative to total IgA and IgG in saliva. We also measure Ag-specific B cells in peripheral circulation (flow cytometry and ELISPOT) and isolating individual Ag-specific B cells to determine somatic mutations and make mAbs.

To measure innate immune response, we are using proteomic analysis of swabs, tongue brushing and saliva. We are doing bulk RNAseq of peripheral circulation.

**Project Title:** Diversity and Determinants of the Immune-Inflammatory Response to SARS-CoV-2
**Grant number:** U54 CA260591
**Primary Contact:**<br><br>Jane C. Figueiredo, Ph.D.<br>Cedars Sinai Cancer and Department of Medicine, Cedars-Sinai Medical Center<br>Address: 700 N. San Vicente Blvd, Pacific Design Center.G-595F Los Angeles CA 90036<br>Email: jane.figueiredo@cshs.org
**Other collaborators:**<br><br>Name: Akil Merchant, M.D.<br>Affiliation(s): Cedars-Sinai Medical Center<br>Email: akil.merchant@cshs.org<br><br>Name: Karen Reckamp, M.D.<br>Affiliation(s): Cedars-Sinai Medical Center<br>Email: karen.reckamp@cshs.org<br><br>Name: Noah Merin, M.D.<br>Affiliation(s): Cedars-Sinai Medical Center<br>Email: noah.merin@cshs.org<br><br>Name: Gil Y. Melmed, M.D.<br>Affiliation(s): Cedars-Sinai Medical Center<br>Email: gil.melmed@cshs.org<br><br>Name: Dermot McGovern, M.D.<br>Affiliation(s): Cedars-Sinai Medical Center<br>Email: dermot.mcgovern@cshs.org<br><br>Name: Jon Braun, M.D.<br>Affiliation(s): Cedars-Sinai Medical Center<br>Email: jonathan.braun2@cshs.org
**Project Aims:**<br><br>Primary aims of interest include:<br>• Identify distinct trajectories and determinants of susceptibility to SARS-CoV-2<br>• Determine vaccine response in health care workers and immunocompromised individuals<br>• Determine immune-inflammatory profiles associated with susceptibility to SARS-CoV-2 and vaccine response
**Primary study design:** Prospective cohort study with repeated questionnaires and biospecimen collection.
**Study population:** Our primary focus is to study health care workers with a range of health conditions and selected immunocompromised populations. Groups of immunocompromised populations include:<br>• Cancer patients: Both patients actively undergoing systemic cancer treatments (primarily immunotherapies and aromatase inhibitors) and long-term cancer survivors. Our focus is on patients with all types of cancer (with the exception of non-melanoma skin cancer) and all stages are eligible to participate.<br>• Idiopathic aplastic anemia patients

- Transplant recipients: This includes hematopoietic cell transplant recipients (autologous and allogeneic).
- Inflammatory Bowel Diseases: This includes Crohn's disease and ulcerative indeterminate colitis.

Age/Sex/Race/Ethnicity: Only adults ≥ 18 years are eligible to participate in this study; all sexes, races and ethnicity are eligible to participate in this study.

Geography: Greater Los Angeles area

Target number enrolled:
Recruitment started November 2020
Total estimated recruitment: up to 2060 health care workers, up to 1,000 cancer patients, 175 IBD

Data collection period:
Start Date: November 3, 2020
End Date: September 30, 2025

Data collection intervals:
Baseline: Pre-vaccination sample
Follow up: 1-2 months post vaccine and/or every 6 months thereafter for 2 years.

Data collection methods:
Survey data and EHR data abstraction.

Common Data Elements:
- Demographic information: Age, race, sex, ethnicity, race, socioeconomic factors
- Clinical/Treatment history: Disease-related and associated treatments
- General medical history: Co-morbidities (obesity, hypertension, CVD, diabetes etc.)
- Behaviors (e.g. smoking, alcohol intake, physical activity, diet)
- Childhood and COVID immunization history
- COVID vaccine knowledge and perspectives
- COVID risk factors (e.g. travel history, exposure to known positive cases etc.)
- COVID medical history (e.g. hospitalization etc.)
- NIH PROMIS scales (loneliness, cognitive function, social isolation)
- Pittsburgh Sleep Quality Index
- Pandemic impact on healthcare, finances and well being

Specimens collected:
Plasma, cryopreserved PBMCs, buffy coat for below assays and future use

Serology/other assays:
- Measure total antibody levels using Abbott Technologies (antibody levels to RBD and N protein)
- T cell repertoire using Adaptive

Project Title: Culturally-targeted communication to promote SARS-CoV-2 antibody testing in saliva: Enabling evaluation of inflammatory pathways in COVID-19 racial disparities

Grant number: U01CA260469

Primary contact:

Todd Lucas, Ph.D.
Division of Public Health, College of Human Medicine, Michigan State University, Flint, MI, USA
Department of Epidemiology and Biostatistics, College of Human Medicine,
Michigan State University, East Lansing, MI USA
Address: 200 East 1st St, Flint, MI 48502
Email: lucastod@msu.edu

Other key investigators:

Douglas A. Granger, Institute for Interdisciplinary Salivary Bioscience Research, University of California Irvine, Irvine, CA, USA and Department of Pediatrics, Johns Hopkins University School of Medicine, Baltimore, MD, USA Email: dagrange@uci.edu

Steve W. Granger, Salimetrics, LLC, Carlsbad, CA, USA
Email:sgranger@salimetrics.com

Christopher D. Heaney, Department of Environmental Health and Engineering, Bloomberg School of Public Health, Johns Hopkins University, Baltimore, Maryland, USA; 2) Department of Chemical and Biomolecular Engineering, Whiting School of Engineering, Johns Hopkins University, Baltimore, Maryland, USA; 3)Department of International Health, Bloomberg School of Public Health, Johns Hopkins University, Baltimore, Maryland, USA Email: cheaney1@jhu.edu

Ahnalee M. Brincks, Department of Human Development and Family Studies, College of Social Science, Michigan States University, East Lansing, MI, USA. Email: brincksa@msu.edu

Debra Furr-Holden, Division of Public Health, College of Human Medicine, Michigan State University, Flint, MI, USA; Email: holdenc3@msu.edu

Kent Key, Division of Public Health, College of Human Medicine, Michigan State University Flint,MI, USA
Email: keykent@msu.edu

Maria Knight Lapinski, Department of Communication, Michigan AgBio Research, Michigan State University, East Lansing, MI, USA. Email:lapinsk3@msu.edu

Nicole Jones, Division of Public Health, College of Human Medicine, Michigan State University Flint, MI, USA 2) Department of Pediatrics and Human Development, College of Human Medicine, Michigan State University, East Lansing, MI, USA Email: warnerni@msu.edu

Nigel Paneth, Department of Epidemiology and Biostatistics College of Human Medicine, Michigan State University; 2) Department of Pediatrics and Human Development, College of Human Medicine, Michigan State University, East Lansing, MI, USA. Email:paneth@msu.edu

Project Aims:

1. Develop and compare effects of a general versus culturally-targeted video about antibody testing on African American and White Flint residents' salivary antibody testing attitudes, intentions, and behavior. We expect that uptake of salivary antibody testing will be higher among White than African American Flint residents when a

general video tutorial is used, but that racial differences in antibody testing interest and uptake will be attenuated by presenting a culturally-targeted video tutorial to African Americans.

2. Identify and compare effects of a general versus culturally-targeted video on activation of medical mistrust and racism-related cognition among African Americans when considering salivary antibody testing. We expect that culturally-targeted information about antibody testing will reduce medical mistrust and racism among African Americans. In turn, we expect that reduced medical mistrust and racism will act as mechanisms linking culturally-targeted video information to greater antibody testing interest and uptake.

3. Measure and identify multi-analyte inflammatory biomarker profiles among Flint Registry enrollees who complete salivary antibody testing, and we will compare inflammatory biomarker profiles by race and antibody status. We expect positive SARS-CoV-2 infection will be associated with higher pro-inflammatory and lower anti-inflammatory profiles. We further expect that differences in these profiles as a function of antibody status will be more pronounced in African Americans than in Whites.

Primary study design:

5-year U01 that will use a 2 (Race: African American versus White) x 2 (Video Tutorial: general versus culturally-targeted) quasi-experimental design. Race is a subject variable whereas video tutorial will be evenly randomly assigned to African American participants. As culturally-targeted modifications are intended for African American participants, White American participants will only be assigned to the general video tutorial condition. This study is considered a randomized control trial.

Study population:

We will recruit 500 screening eligible participants. The Flint Registry – a CDC-supported and highly visible health resource exchange that connects Flint residents to health programs and services in the wake of the Flint water crisis – will provide the population frame from which the proposed research sample will be drawn. The Flint Registry maintains a panel of over 10,000 fully enrolled individuals, including email contact information. Our criteria for inclusion are being an African American or White American individual 18 years and over and enrolled in the Flint Registry. We will measure but not exclude based on prior SARS-CoV-2 confirmed infection or vaccination.

Age/Sex/Race/Ethnicity:

This study will only a two-thirds African-American sample. African-Americans make up over 60% of Flint and have been found to be at increased risk SARS-CoV-2 infection and death. African Americans also make up over two-thirds of enrollees in the Flint Registry from which we will recruit.

This study will recruit both male and female participants. All participants will be 18 years old and over. We aim to recruit a total sample size of 500 individuals. We will stratify by age and recruit even sample sizes of N = 167 children 18-21, young adults 22-40; and older adults 40 and above. Each age group will be further stratified by race and gender. We will recruit a two-thirds African American sample (N = 110), and a one-third White sample (N = 57) in all age groups, both of which will be one-third to one-half male.

The inclusion of both a one-third White sample and a one-third male sub-sample will ensure that we are able to conduct effective race and gender comparisons.

Geography:

This study will take place in Flint, Michigan. Flint is located in Southeast Michigan – a region that encompasses the Detroit Metropolitan area, which includes a culturally and ethnically diverse population of approximately 4 million people. Over 30 percent of the metropolitan area population are members of ethnic and racial minorities. In the 2010 census, the city of Detroit had the third largest population of African-Americans among all cities in the nation and ranked first in the percentage (81.6%) of African Americans among all cities over 100,000 in

population. The Michigan State University College of Human Medicine is located in Flint, Michigan. Flint is the largest city in Genesee County, and is located approximately 70 miles northwest of Detroit. The Flint Metropolitan area is the fourth largest in the state. Flint has a population of over 102,000 and is the seventh largest city. The city of Flint encompasses an area of socioeconomic hardship. Flint became known to many in 2014 because of lead poisoning that occurred in its local water supply, causing a public health emergency. Like Detroit, Flint is a majority African American community (56.6% in 2010 census).
**Current/Target number enrolled:**<br><br>No planned or actual enrollment prior to 5/1/21. Planned total enrollment N = 500
**Data collection period:**<br><br>We anticipate data collection to begin starting 6/21 and running through 6/24
**Data collection intervals:**<br><br>At present collecting only at time 0. We anticipate adding longitudinal data collections over the life of the project (years 2-4).
**Data collection methods:**<br><br>Patient reported outcomes via electronic surveys.
**Data elements:**<br><br>We will collect (or have access to) a full panel of sociodemographic information that includes age, race, socioeconomic status, and health comorbidities. Our project especially emphasizes collection of psychosocial measures that include but are not limited to subjective social status, medical mistrust, perceived fairness, anticipatory racism, and healthcare cultural competency.
**Specimens collected:**<br><br>This project will utilize salivary data collection. This includes collection of saliva using an Oracol S14 saliva collection device, which will collect gingival crevicular fluid (GCF). The project will also collect passive drool salivary samples.<br><br>With permission granted by study participants, we anticipate that reserve capacities of passive drool salivary samples will be available for future use.
**Serology/other assays:**<br><br>1) We will use saliva collection to administer a multiplex salivary antibody assay. The assay measures 10 or more SARS-CoV-2 antibodies including but not limited to anti-nucleocapsid, RBD. IgG, IgA and IgM.<br><br>2) We will use saliva to measure a comprehensive panel of inflammatory markers. These include but are not limited to IFN-γ, TNF-α, IL-1β, IL-2, IL-5, IL-6, IL-7, IL-8, IL-10, IL-12p70, IL-13, IL-17A

**Project Title:** COVID and cancer, obesity, immune compromise, and other conditions
**Grant number:** U54 CA260563-01
**Primary contact:**<br><br>John D. Roback, MD PhD<br>Affiliations: Department of Pathology and Laboratory Medicine, Emory University School of Medicine<br>Address: EUH D655, 1364 Clifton Rd NE, Atlanta, GA 30322<br>Phone number: 404-712-1774<br>Email: jroback@emory.edu
**Other collaborators:**<br><br>Name: Rafick Sekaly, PhD<br>Affiliation(s): Department of Pathology and Laboratory Medicine, Emory University School of Medicine<br>Email: rafick.sekaly@emory.edu<br><br>Name: Jens Wrammert, PhD<br>Affiliation(s): Department of Pediatrics, Emory University School of Medicine<br>Email: jwramme@emory.edu<br><br>Name: Muhammad Ali, PhD<br>Affiliation(s): Department of Pathology and Laboratory Medicine, Emory University School of Medicine<br>Email: muhammad.ali@emory.edu<br><br>Name: Andrew Neish, MD<br>Affiliation(s): Department of Pathology and Laboratory Medicine, Emory University School of Medicine<br>Email: aneish@emory.edu
**Institution(s):**<br><br>Emory University School of Medicine
**Project Aims:**<br><br>**Aim 1:** To characterize the magnitude, repertoire and durability of SARS-CoV-2 specific B cell memory in cancer patients compared to non-cancer patients, including those with obesity, immune dysfunction, and other conditions that could impair the immune response.<br><br>**Aim 2:** To identify the mechanisms triggered by the inflammatory environment prevalent in a cohort of cancer patients during acute infection which impair the Ab response to SARS-CoV-2 infection, and to compare with patients with with obesity, immune dysfunction, and other conditions.<br><br>**Aim 3:** To identify the transcriptional and epigenetic mechanisms triggered by viral infection in these patients that will impede the longevity of memory B cells and function of Tfh cells.
**Primary study design:**<br><br>Hospital inpatients newly admitted due to a positive SARS-CoV-2 RT-PCR test on a nasopharyngeal swab are consented for blood draws. Blood draws are performed shortly after admission, at discharge, and then at 3- and 6-months after discharge. Samples are studies as per the Project Aims. In addition, the admission NP swab is collected, when possible, to support sequencing to determine viral strain.

**Study population:**<br>Hospital inpatients newly admitted due to a positive SARS-CoV-2 RT-PCR test on a nasopharyngeal swab. Emphasis is given to recruiting patients with cancer, obesity, immune compromise, and other conditions which could affect the viral immune response.
**Age/Sex/Race/Ethnicity:**<br>Age range: 23-81<br>Sex: 41 male, 52 female<br>Race: 59 African American, 33 Caucasian, 1 Unknown<br>Ethnicity: 2 Hispanic, 88 Non-Hispanic, 3 Unknown
**Geography:**<br><br>Atlanta and surrounding communities
**Current/Target number enrolled:**<br>93 enrolled to date (1 enrolled prior to May 1, 2021); we plan to continue enrollment during each viral wave in Atlanta
**Data collection period:**<br>4/25/21 – present
**Data collection intervals:**<br><br>Samples collected upon admission to hospital with positive test for SARS-CoV-2, upon hospital discharge, and then 3 and 6 months after discharge
**Data collection methods:**<br><br>Electronic health record data abstraction, blood sample collections and testing, collection of nasopharyngeal swabs and testing
**Data elements:**<br>Patient name, Participant ID, Medical Record Number, Gender, Race, Ethnicity, Date of Birth, Age, Address, Phone, Symptom Onset, Admission Date, Discharge Date, Date of Blood Collections, Date of NP Swab Collection, Type 1 or Type 2 DM, Transplant, Cancer, HIV, Lupus, Obesity, Asthma, Crohn's Disease, Immunosuppression, Chronic Kidney Disease, Cystic Fibrosis, BMI, CRP, D-dimer, CPK, Troponin, Ferritin, LDH, Absolute Lymphocyte Count, WBC, Albumin, AST, Insulin Dosage, Oral Hypoglycemic Agents, Other Medications, Respiratory Therapy
**Specimens collected:**<br>Plasma, PBMCs, NP swabs
**Serology/other assays:**<br>Serology, NGS of NP swab sample, flow cytometry, ELISPOT, viral neutralization, RNA-Seq, multiplex cytokine assays, metabolomics

**Project Title:** "Serological Sciences Network Capacity Building Center"
**Grant number:**<br>Leidos Subcontract Number 21X090
**Primary contact:**<br><br>Peter K Gregersen, MD<br>Affiliations: Professor and Head, Robert S. Boas Center for Genomics and Human Genetics, Feinstein Institutes for Medical Research, Northwell Health<br>Address: 350 Community Drive, Manhasset, NY 11030<br>Phone number: 516-562-1542<br>Email: pgregers@northwell.edu
**Co-investigators:**<br><br>Name: Betty Diamond, MD<br>Affiliation(s): Director and Professor, Institute of Molecular Medicine, Feinstein Institutes for Medical Research; Professor, Departments of Molecular Medicine and Medicine, Donald and Barbara Zucker School of Medicine at Hofstra/Northwell<br>Email: BDiamond@northwell.edu<br><br>Name: James M Crawford, MD PhD<br>Affiliation(s): Professor and Chair, Department of Pathology and Laboratory Medicine, Donald and Barbara Zucker School of Medicine at Hofstra/Northwell; Professor, Institute of Health System Science; Feinstein Institutes for Medical Research; Senior Vice President for Laboratory Services; Northwell Health<br>Email: jcrawford1@northwell.edu
**Institution(s):**<br><br>Feinstein Institutes for Medical Research, Northwell Health
**Project Aims:**<br><br>Aim 1: Control Population Response to COVID-19 Vaccination.<br>Aim 2: Response of an Autoimmune Population to COVID-19 Vaccination.
**Primary study design:**<br><br>**Aim 1.** Recognizing the increasing challenge of SeroNet institutions identifying pre-immune Human Subjects who have not been vaccinated, Feinstein/Northwell will leverage our continuing robust regional vaccination programming to identify Human Subjects at the time of the first vaccination and during the same encounter, obtain pre-immune blood samples for serum and PBMC harvesting. Post-vaccination samples will be obtained at scheduled intervals, to evaluate the host response of both B-cell and T-cell mediated immunity.<br>**Aim 2.** Feinstein/Northwell will access the population of patients with autoimmune disorders to evaluate their post-vaccination immune status. Although we will attempt to obtain pre-vaccination blood samples, since vaccination has been a high priority in these populations, our fundamental goal will be to obtain samples within 2 months post-vaccination and obtain further blood samples at scheduled intervals. This Human Subjects will include patients with Systemic Lupus Erythematosus, Sjögren's Syndrome, and Rheumatoid Arthritis.
**Study population:**<br>Aim 1. Control Human Subjects (without Autoimmune Conditions): The target recruitment is 700 individuals.<br>Aim 2. Human Subjects with Autoimmune Conditions (Systemic Lupus Erythematosus; Sjögren's Syndrome, Rheumatoid Arthritis: The target recruitment is 400 individuals.

Age/Sex/Race/Ethnicity:
Aim 1. Human Subject Recruitment will be representative of the age/sex/race/ethnicity of our regional community, on the basis of individuals who consent to be participants in this study.
Aim 2. Human Subject Recruitment will be representative of the age/sex/race/ethnicity of the patient populations in the respective "census" of the Northwell programs, who consent to be participants in this study.

Geography:
Northwell Health serves the greater New York Metropolitan area, inclusive of Nassau and Suffolk Counties (Long Island), Westchester County (Hudson Valley), and the New York City boroughs of Queens, Manhattan, Brooklyn, Bronx, and Staten Island.

Current/Target number enrolled:
The recruitment protocol is under review by SeroNet at this time.

Data collection period:
Start date for both Aims will be as soon as the recruitment protocol is approved by SeroNet (target: June 2021). End date for enrollment is when target recruitment has been achieved (target: December 2021).

Data collection intervals:

Aim 1. "Pre-immune" blood samples: at time of 1st vaccination visit. "Post-vaccination" blood samples: at 2 months ± 14 days post 1st vaccine dose; 6 months ± 14 days; 1 year ± 14 days; and 2 years ± 14 days.
Aim 2. "Pre-immune" blood samples (if at all possible; target 5% of total enrollment): at time of 1st vaccination visit. Most likely first blood samples: 2 months ± 14 days post 1st vaccine dose; then 6 months ± 14 days; 1 year ± 14 days; and 2 years ± 14 days.

Data collection methods:

Electronic health record data abstraction (including COVID-19 PCR and antibody test results); laboratory evaluation of host T-cell immune response.

Data elements:
Age, Gender, Race/Ethnicity (as available), Zip Code, Comorbidities, SARS-CoV-2 PCR test results, SARS-CoV-2 Antibody test results, Treatments (for Human Subjects with Autoimmune Conditions)

Specimens collected:
Whole blood for preparation of serum and of Peripheral Blood Mononuclear Cells (PBMCs)

Serology/other assays:
SARS-CoV-2 PCR assays: GenMark, Cepheid, BioFire, Abbott, Roche
SARS-CoV-2 Antibody assays: Roche, DiaSorin

Project title: Characterization of the Antibody Response to SARS-CoV-2 in Lung Cancer Patients

Grant number: U54 CA260560

Primary contact:

Fred R. Hirsch, MD, PhD
Icahn School of Medicine at Mount Sinai
One Gustave L. Levy Place. New York, NY 10029
Tel: (212) 241-8051
Email: Fred.Hirsch@mssm.edu

Co-investigators:

Emanuela Taioli
Icahn School of Medicine at Mount Sinai
Email: Emanuela.Taioli@mountsinai.org

Institution(s):

Icahn School of Medicine at Mount Sinai

Project Aims:

Aims: 1) To understand factors contributing to the vulnerability of SARS-CoV-2 infection in patients with lung cancer. **2)** To characterize and compare the antibody response to SARS-CoV-2 infection and SARS-CoV-2 vaccines in patients with lung cancer compared to a matched "healthy" control group

Primary study design:
This is a non-interventional, longitudinal study of COVID-19-induced blood antibody levels in lung cancer patients exposed to this virus or receiving vaccination. Control populations include lung cancer patients with no evidence of disease and healthy volunteers (non-exposed, exposed, and vaccinated). There are no drug treatments or other interventions specified by this study.

Study population:

Research will be conducted in the oncology clinics at all of the clinical locations of the Mount Sinai Hospital System.

Age/Sex/Race/Ethnicity:

Participants are over the age of 18 years. Recruitment to this study will take place in the oncology and lung cancer screening clinics of the Mt Sinai Health System. Subject demographics will follow the known breakdown of lung cancer patients at MSHS, seen below.

Facility	Race		
	White	Black	Asian
MSH	975	284	137
MSBI	262	132	127
MSW	159	149	21

Facility	Ethnicity		
	Hispanic	Non-Hispanic	Unknown
MSH	200	1747	92
MSBI	77	613	19
MSW	72	395	15

Geography: New York, NY

Current/Target number enrolled:

The target number for enrollment is 2,000.

Data collection period:

October 2020- April 2024

Data collection intervals:

Participants will be actively enrolled over a 3.5 year time period

Data collection methods:

Health records and in person visits

Data elements:

Name, Telephone number, Address, date of birth, date of admission, date of discharge, date of death, Medical Record Number, Medical history, concurrent medications, results of physical examinations, and results of tests and procedures.

Specimens collected:

Blood samples

Serology/other assays:

Control subject characteristics will be obtained by ELCAP. Subjects will be followed for two years, with blood serology draws obtained at the same schedule as cases (Baseline 3, 6, 12, 24 months). For the post-vaccination series, the control group will segue to non-lung cancer, vaccinated individuals. This post-vaccine "healthy control" group will be collected in exactly the same fashion but known to have had – or are immediately receiving on study entry – an approved vaccination for SARS-CoV-2; in all ways analogously to the lung cancer group.

**Project title:**<br>"Serological Sciences Network Capacity Building Center"
**Grant number:**<br>Leidos Subcontract Number 21X090
**Primary contact:**<br><br>Carlos Cordon Cardo, MD<br>Irene Heinz Given and John LaPorte Given Professor and Chairman for the Mount Sinai Health System Department of Pathology
**All other collaborators:**<br><br>Viviana Simon, MD; Ania Wajnberg, MD; Florian Krammer, MD; Samir Parekh, MD; Serre Yu Wong, MD; Lynne Richardson, MD; Meenakshi Rana, MD
**Institution(s):**<br>Icahn School of Medicine at Mount Sinai
**Project Aims:**<br>• We will acquire and conduct quality control assessments of critical reference samples.<br>• We will study convalescent immunity in COVID-19 survivors with and without different co-morbidities (Inflammatory Bowel Disease, Multiple Myeloma, Transplant) and in people of diverse backgrounds seeking care in our Emergency Department.<br>• Control Population Response to COVID-19 Vaccination in a largely healthy adult control population of COVID-19 survivors.<br>• Immune Responses to COVID-19 vaccination of populations with various co-morbidities (Inflammatory Bowel Disease, Multiple Myeloma, Transplant) and in people of diverse backgrounds seeking care in our Emergency Department.
**Primary study design:**<br>- Recruit participants and collect samples to characterize immune response to COVID-19 as well as vaccination in healthy participants to one, two and three vaccine doses.<br>- Recruit participants and collect samples to characterize immune response to COVID-19 as well as vaccination in participants with either IBD, MM, solid organ transplant, ED use to one, two and three vaccine doses.
**Study population:**<br>• Post-vaccination serosurveillance: COVID-19 survivors with and without different co-morbidities (Inflammatory Bowel Disease, Multiple Myeloma, Transplant) and in people of diverse backgrounds seeking care in our Emergency Department.<br>- Natural infection serosurveillance: COVID-19 survivors with various co-morbidities (Inflammatory Bowel Disease, Multiple Myeloma, Transplant) and in people of diverse backgrounds seeking care in our Emergency Department
**Age/Sex/Race/Ethnicity:**<br>• Subject Recruitment will be representative of the age/sex/race/ethnicity of our regional community as long as they are 18 years or older. We do not exclude study subjects based on age, gender, race, or ethnicity.
**Geography:**<br>New York City metropolitan area.

**Current/Target number enrolled:**<br>Currently enrolled up to October 1 2021: 250 critical reference sample participants and 500 participants in surveillance studies<br>Post-vaccination serosurveillance: 600 participants with and without IBD, MM, solid organ transplant
**Data collection period:**<br><br>February 2021-October 2023
**Data collection intervals:**<br><br>Pre vaccine (if feasible), longitudinally at 3, 6, 12, 24 months post vaccination.
**Data collection methods:**<br><br>Electronic health record data abstraction, collaboration with primary clinical partners and their medical teams.
**Data elements:**<br><br>Age, Gender, Race/Ethnicity (as available), Comorbidities, SARS-CoV-2 PCR test results, SARS-CoV-2 Antibody test results, Treatments
**Specimens collected:**<br><br>Whole blood for preparation of serum and of Peripheral Blood Mononuclear Cells (PBMCs)
**Serology/other assays:**<br><br>Mount Sinai/Kantaro ; Enzyme Linked Immunosorbent Assay (ELISA)

Project Title:
Johns Hopkins Excellence in Pathogenesis and Immunity Center for SARS-CoV-2 (JH-EPICS)

Grant number:
1 U54 CA260492-01

Primary contact:

Andrea L. Cox, M.D., Ph.D.
Affiliations: Johns Hopkins School of Medicine
Address: Rangos Rm 551, 855 N. Wolfe St, Baltimore, MD 21205
Phone number:410-502-2715
Email: acox@jhmi.edu

All other collaborators:

Name: Sabra L. Klein
Affiliation(s): Johns Hopkins Bloomberg School of Public Health
Email:sklein2@jhu.edu

Name: Andrew H. Karaba
Affiliation(s): Johns Hopkins School of Medicine
Email: Andrew.Karaba@jhmi.edu

Institution(s):
Johns Hopkins University

Project Aims:
JH-EPICS includes 3 research projects, each built upon the same foundation and sample access.

Research Project 1 Aims:

1. Establish the mechanism by which SARS-CoV-2 activates the inflammasome and determine how inhibition of this pathway alters innate immune signaling.
2. Define the impact of antibodies produced in COVID-19 on innate sensing of SARS-CoV-2.
3. Characterize the unique populations of MDSCs present in PBMC of COVID-19 and determine their role in COVID-19 recovery and pathogenesis.

Research Project 2 Aims:

1. Track the unique population of immune cells present in the PBMC of COVID-19 patients as biomarkers to follow disease course and predict severity of disease. In addition, employ this flow cytometry-based assay to identify surrogate markers for the testing of novel therapeutic agents.
2. By employing the FEST assay, test the hypothesis that disease pathogenesis is linked in part to mitochondrial-mediated apoptosis resulting in the deletion of viral antigen-specific T cells.
3. Dissect the metabolic programs driving the differentiation and function of the unique population of T cells present in the PBMC of COVID-19 patients.
4. Define immune cell metabolic profiles and phenotypes in solid organ transplant recipients that distinguish COVID-19 vaccine responders and non-responders

Research Project 3 Aims:

1. Determine the extent and duration of the serum antibody response against SARS-CoV-2 infection by measuring IgM, IgG (total and subclasses), and IgA (monomeric and dimeric) that recognize the SARS-CoV-2 S protein and the S receptor binding domain (S-RBD).
2. Characterize the kinetics and duration of the nAb response against SARS-CoV-2 and the ability of virus to escape from nAbs.

3. Analyze the function of non-neutralizing SARS-CoV-2-specific serological response by assessing antibody-dependent cellular cytotoxicity (ADCC), complement-mediated cytotoxicity, and complement fixation activity toward SARS-CoV-2 virus particles and virus-infected cells.

Cross-SeroNet Collaboration with Harvard U01:
Sex differences in systems serological responses to COVID-19 mRNA vaccines.

Primary study design:
Each Research Project has individual design components related to each of their specific aims. The primary design of JH-EPICS collectively is Research Projects working from access to a biorepository
of samples from a large, ongoing prospective cohort of patients to explore each pr individual aims, supported by a Virology Resource Core and an Analysis Resource Core to integrate data from the three RPs and develop analytical and predictive models to define the correlates of severe disease versus protection and identify the intersection of diverse demographic and clinical variables that serve as modifying factors affecting immune and serological responses to SARSCoV-2 infection.

Study population:
The COVID-19 Prospective Cohort. From April of 2020, all patients diagnosed with COVID-19 in the Johns Hopkins Health Care System have been invited to enroll in the COVID-19 Prospective Cohort regardless of disease severity or underlying medical conditions. Upon consent, sampling of hospitalized and non-hospitalized (ambulatory) patients was carried out and samples were compiled and stored in a central biorepository.

Age/Sex/Race/Ethnicity:
The population has grown since the below information was compiled and as such the demographics have likely changed somewhat. Additionally, the current demographics of the samples analyzed to date by the Research Projects differs across projects slightly as the process is ongoing. However, the information below does give a good insight into the demographic make-up of our study population:

The demographics of the first 160 hospitalized study patients enrolled with blood samples in the biospecimen repository demonstrate the tremendous diversity in race, ethnicity, underlying comorbidities, and disease severity of this cohort:

Prospective Cohort: characteristics of the first 160 hospitalized participants enrolled.
Demographics
Male N (%) 84 (52.5)
Female N (%) 76 (47.5)
Mean age (range) 56 (20-85)
Mean BMI (range) 32 (16.8-69.7)
Current smoker N (%)
6 (3.8)
Race and Ethnicity
Race N (%)
Black 78 (48.8)
Other* 43 (26.9)
White 32 (20)
Asian 3 (1.9)
Unknown 2 (1.3)
Native American 1 (0.6)
Pacific Islander 1 (0.6)
Ethnicity
Hispanic/Latinx
N (%)
Yes 45 (28)
No

115 (72)
Maximum Disease Severity** N (%)
MinO2 104 (65.0)
HFO2 14 (8.8)
Ventilated Lived 31(19.4)
Died 11 (6.9)
Comorbidities***
N (%)
Hypertension 86 (53.8)
Diabetes mellitus 59 (36.9)
COPD/asthma 40 (25.0)
Cancer
Coronary artery disease
26 (16.3)
14 (8.8)
Autoimmune diseases 13 (8.1)
Solid organ transplant 9 (5.6)
Hepatitis C virus infection 6 (3.8)
HIV infection 5 (3.1)

*Most self-identified as Hispanic/Latinx.
**Maximum disease severity indicates the most severe COVID-19 disease class for the patient while under observation: MinO2= no or low flow oxygen required, HFO2= high flow oxygen required, Ventilated= patient required intubation and survived, Died = patient died (ventilated or not)
***Comorbidities seen in > 3% of participants are listed

We have now enrolled 723 participants with COVID-19 infection and 245 solid organ transplant recipients pre and post vaccination.

Geography:
The geographic area encompasses much of the Baltimore-Washington metro area.

Current/Target number enrolled:
As of May 1, 2021: 478 participants enrolled (analyses are selected from this pool pending particular follow up sample availability)

Total planned is pending overall Johns Hopkins COVID-19 hospital burden, but estimated to be 2000.

Data collection period:
April 2020 – 24 months post final enrollment

Data collection intervals:
Hospitalized Patients:
Diagnosis (Day 0), Day 1, Day 3, Day 7, Weekly, Day 28, Month 3, 6, 9, 12, 18, 24

Ambulatory Patients:
Diagnosis (Day 0), Day 28, Month 3, 6, 9, 12, 18, 24
(self-swab and serum collection prior to day 28 while quarantined)

Data collection methods:
In person visits, electronic health record data abstraction.

Data elements:
Age, sex, race, ethnicity, disease severity, BMI, comorbidities, clinical information,

**Specimens collected:**<br>Depending on the visit:<br>Serum, plasma, NP swab, OP swab, PBMC
**Serologic and other assays:** Mesoscale Discovery Assay (MSD) and ELISA to assay antibodies and antibody subtypes directed against SARS-CoV-2 proteins, MSD assays for cytokines and chemokines, metabolic immune cell flow cytometry, virus neutralization assays, antibody-dependent cellular cytotoxicity, complement-mediated cytotoxicity, complement fixation, ViraFEST, and ELISpot.

Project Title: SARS-CoV-2 Serological Antibody Testing for Disease Surveillance and Clinical Use

Grant number: 1U01CA260584-01

Primary contact: Jacek Skarbinski, MD
Kaiser Permanente Northern California (KPNC) Division of Research
2000 Broadway, Oakland, CA 94612
Email: Jacek. Skarbinski@kp.org

Other key investigators
Lawrence Kushi and Douglas Corley
Kaiser Permanente Northern California
Emails: Larry.Kushi@kp.org; Douglas.A.Corley@kp.org

Project Aims:

- Assess prevalence and incidence of SARS-CoV-2 antibody sero-positivity in a representative sample of community-dwelling persons aged ≥7 years. Hypothesis: Prevalence and incidence of SARS-CoV-2 antibody sero-positivity will be low but will vary based on demographic, behavioral and clinical characteristics.
- Assess factors associated with SARS-CoV-2 sero-conversion and longevity of antibody response among a prospective cohort study of SARS-CoV-2-antibody positive individuals with serial SARS-CoV-2 antibody testing and EHR-based longitudinal follow-up. Hypothesis: Development of SARS-CoV-2 antibodies and longevity of antibody response will vary dependent on demographic, clinical, and COVID-19 specific factors.
- Assess factors for incident COVID-19 disease among persons positive for viremia, sero-positive for SARS-CoV-2 antibodies, or who have previously had COVID-19 disease. Hypothesis: Recurrent COVID-19 disease is rare, but few large-scale prospective studies have been conducted to assess for recurrent COVID-19.

Study Design: Prospective cohort study

Study Population: The study population is all KPNC membership >= 7 years old, with a valid email address that speak English, Spanish, Vietnamese, Chinese, or Tagalog

Age/Sex/Race/Ethnicity: KPNC members aged ≥7 years (Aim 1), ≥18 years (Aim 2), stratified by age group and area of residence, and race/ethnicity

Geography: All KPNC service areas in Northern and Central California: Central Valley (Modesto, Manteca), Diablo (Antioch, Walnut Creek), East Bay (Richmond, Oakland), Fresno, Greater Southern Alameda County (San Leandro, Fremont), Marin Sonoma, Napa Sonoma, Greater Sacramento, Greater San Francisco, Peninsula and South Bay (Redwood City, Santa Clara, San Jose)

Current/Target number enrolled:
Number enrolled up to May 1, 2021: 0
Planned total enrollment: 40,000

Data collection period:
Start date: 5/3/2021
End date: 10/1/2023

Data collection intervals:
Aim 1: 0, 3 months
Aim 2: 0, 3, 6, 12, 24 months

Data collection methods:
EMR, PRO via electronic surveys

Data elements:

Summary and description of data domains and data sources

**Data domain**	**Data source**	**Description**
Demographic	HER	• Age, sex, race/ethnicity, zip code of residence, socioeconomic status using cens variables, insurance status, length of KPNC membership
Behavioral	HER	• Smoking, alcohol use
Clinical	EHR;<br>CA OSHPD*	• Co-morbidities, including cancer, obesity, diabetes, hypertension, cardiovascula infection, immunosuppression<br>• Medications, Imaging<br>• Events such as hospitalization, admittance to intensive care unit, ARDS, and de
COVID-19 testing, treatment, and prevention	HER	• SARS-CoV-2 PCR and antibody testing as part of routine care<br>• COVID-19 disease severity: no history of COVID-19, asymptomatic outpatient outpatient, hospitalized, critically ill requiring ICU care<br>• COVID-19 treatments: Remdesivir, corticosteroids, COVID-19 convalescent pl immunomodulators<br>• SARS-CoV-2 vaccine: if available
SARS-CoV-2 antibody testing	Study testing	• SARS-CoV-2 antibody testing as part of this study
COVID-19 behaviors	Study questionnaire	• Occupation, compliance with shelter-in-place orders, demographic and househo characteristics, exposure to persons with COVID-19, COVID-19 symptoms sin

* CA OSHPD: California Office of Statewide Health Planning & Development (statewide hospitalization databases

Specimens collected:
Blood; not stored for future use.

Serology/other Assays:
Commercially available serology assays. Orthogonal testing algorithm using Siemens SARS-CoV-2 IgG assay and DiaSorin Liaison SARS-CoV-2 S1/S2 IgG assay

**Project Title:** Center for Serological Testing to Improve Outcomes from Pandemic COVID-19 (STOP-COVID)
**Grant number:** 1 U54 CA260582-01
**Primary contact:**<br><br>Ashish Panchal, MD<br>Affiliations: Department of Emergency Medicine, The Ohio State University Wexner Medical Center<br>Address: 760 Prior Hall, 376 West 10th Avenue, Columbus, OH 43210<br>Phone number: 614-366-7880<br>Email: ashish.panchal@osumc.edu
**Other key collaborators:**<br><br>Name: Ann McAlearney<br>Affiliation(s): Department of Family and Community Medicine, College of Medicine, The Ohio State University<br>Email: Ann.McAlearney@osumc.edu<br><br>Name: Eben Kenah<br>Affiliation(s): Division of Biostatistics, College of Public Health, The Ohio State University<br>Email: kenah.1@osu.edu
**Institution(s):**<br><br>The Ohio State University
**Project Aims:**<br><br>The Center to STOP-COVID will: Aim 1 Develop Institute Infrastructure through three shared resource cores: 1. An Administrative Core that provides overall direction and leadership, coordinating all Center activities as well as Project–Core–SeroNet interactions; 2. A Testing and Biorepository Core, whose role is to perform first-tier serologic and viral testing during our longitudinal study using high throughput ELISA and neutralization assays developed at OSU, and cost-shared by OSU; and 3. A Data Management and Analysis Core that will provide project investigators with a centralized resource for biostatistics, bioinformatics, epidemiology, and psychometrics expertise. Aim 2: Conduct three innovative research projects to address: Project 1: Parallel serological and viral testing to determine COVID-19 prevalence, transmission, and protection in extended first responder cohorts. This project will also generate serology data for vaccines or mAbs, once available to this presumably highpriority group; Project 2: Serologic and molecular determinants of COVID-19 severity and immune protection. This project will evaluate COVID-19 serological responses in the context of SARS-CoV-2 and common cold CoV (CCCoV) antibodies, using novel assays specific for a panel of antigens. Project 2 also will employ transcriptomics to understand how host genetics, CCCoV, other respiratory viruses,and immune responses contribute to pathogenesis; and Project 3: Responding to changing serological and viral information around COVID-19. This project will incorporate results from Projects 1 & 2 and SeroNet to inform best practices in risk communication, provide behavioral guidance to decrease transmission, and enhance protection from disease. Aim 3: SeroNet Participation and Sharing of Data and Best Practices. We will leverage STOP-COVID infrastructure to share data, results, reagents, and best practices with SeroNet, which will drive new discoveries and their translation into actionable strategies for implementation across all groups affected by COVID-19.
**Primary study design:**<br><br>The Center for Serological Testing to Improve Outcomes from Pandemic COVID-19 (STOP- COVID) is proposed as a transdisciplinary entity to understand the interface between exposure risk, transmission, immune responses, disease severity, protection, and barriers to testing/vaccination, with the goal of improving population health and clinical outcomes. The Center will utilize state-of-the-art serological and molecular tests, developed at

OSU, in a longitudinal study of first responders, a group at continual high risk of SARS-CoV-2 exposure, as well as their household contacts. Through the proposed work, STOP-COVID investigators will understand critical aspects of: (i) transmission in both asymptomatic and symptomatic individuals, (ii) immune, host, and viral determinants of disease outcome, and (iii) factors associated with immune protection. Center investigators will also identify best practices for communication of test results and information about COVID-19 to improve understanding of risk, transmission, and protection, while reducing access barriers to testing.
**Study population:**<br><br>First responders, healthcare workers, and their household contacts
**Age/Sex/Race/Ethnicity:**<br><br>First responders and healthcare workers are adults. Household contacts under the age of 18 will be included only if the primary subject becomes symptomatic. No limitations by sex, race, or ethnicity.
**Geography:**<br><br>The Central Ohio area within approximately a 45-minute drive from The Ohio State University Wexner Medical Center.
**Current/Target number enrolled:**<br><br>97 enrolled as of May 1, 2021 out of a planned total enrollment of 2,500.
**Data collection period:**<br><br>Data collection began on February 10, 2021. It is planned to continue throughout the five-year funding period, which ends in 08/2026.
**Data collection intervals:**<br><br>Initial sample collection is completed after full consent, subsequent sample collections are then completed every 180 days for study length as long as the subjects remain asymptomatic. In the case where the subject become symptomatic, additional sample collections are performed at days 1,7,14, and 25 from the onset of symptoms as long as the subject remains in quarantine.
**Data collection methods:**<br><br>Initial, weekly, and monthly surveys are completed by the subject through REDCap. Clinical antibody (SARS-CoV-2 Spike (S) Protein IgG), COVID-19 PCR positivity status, and saliva sample are collected during initial and subsequent sample collections.
**Data elements:**<br><br>Demographic, occupational information, living situation, community interactions, COVID-19 testing, stress, COVID-19 vaccination status, and basic medical history are collected during the initial survey. Any new onset of symptoms is collected during the weekly surveys. PPE use and potential exposure data is gathered in the initial survey as well as each subsequent monthly survey. Vaccination status and an updated treatment summary are collected at every six-month sample collection.
**Specimens collected:**<br><br>A clinical antibody blood specimen (SARS-CoV-2 Spike (S) Protein IgG) and a nasal swab (SARS-CoV-2 PCR) were collected for immediate processing and results. In addition, saliva sample and biorepository blood

specimens and were collected in cell preparation and serum tubes and processed for future research. In symptomatic patients, saliva samples were also collected for future use.
**Serology/other assays:**<br><br>anti-S (qual), anti-N (qual), trimeric anti-S (qual), unique S peptide alpha, unique N peptide alpha, unique S peptide beta, unique N peptide beta, unique S peptide SARS, unique N peptide SARS, common (cross-reactive) S peptide, common (cross-reactive) N peptide, neutralizing titer WT, neutralizing titer D614G, neutralizing titer B.1.1.7, neutralizing titer B.1.351, neutralizing titer P1, SARS, SARS QC coverage, SARS strain (Pango), SARS strain (GISAID), RSV A, RSV B, influenza A(H3N2), influenza A (H1N1), influenza B, human coronavirus HKU1, human coronavirus OC43, human coronavirus NL63, human coronavirus 229E, human metapneumovirus (HMPV), human adenovirus (HAdV), IFNB1 RNA, and DXVX QC

**Project Title:** Tulane University COVID Antibody and Immunity Network (TUCAIN)
**Grant number:**<br><br>1U54CA260581-01
**Primary contact:**<br><br>John S. Schieffelin, MD<br>Affiliations: Tulane University School of Medicine<br>Address: 1430 Tulane Ave, Box 8408<br>Phone number: 504-988-5117<br>Email: jschieff@tulane.edu
**Other key collaborators:**<br><br>Name: James E. Robinson, MD<br>Affiliation(s): Tulane University School of Medicine<br>Email: jrobisno@tulane.edu<br><br>Name: Monika Dietrich, MD<br>Affiliation(s): Tulane University School of Medicine<br>Email: mdietri@tulane.edu
**Institution(s):**<br><br>Tulane University School of Medicine
**Project Aims:**<br>**Aim 1.** To characterize the evolution, function, and longevity of the humoral immune response to COVID-19.<br>**Aim 2.** To identify cell mediated immune responses that contribute to durable or short-lived humoral immunity to SARS CoV-2<br>**Aim 3.** To correlate protective and potentially pathogenic immune responses to the clinical course of hospitalized COVID-19 patients.
**Primary study design:**<br>Observational cohorts
**Study population:**<br>1. TUCAIN: People living with solid and liquid cancers<br>2. COVID-19 Serostatus and Immunity (CSI): Adults and children with history of COVID-19 infection and/or vaccination<br>3. Allergy Reduction and Child Health Study (ARCHS): Children with asthma<br>4. HIV: Adults living with HIV<br>5. Serostudy: Serostudy of Stored Serum Samples of COVID-19 in Louisiana (Pediatric + Maternal) = analysis of clinical excess serum and plasma samples from a Children's Hospital and from a Women's Hospital
**Age/Sex/Race/Ethnicity:**<br>1. TUCAIN: ≥18 years/males and females/Black, White, Asian/Hispanic and non-Hispanic<br>2. CSI: ≥ 6mos to 99 years/males and females/Black, White, Asian/Hispanic and non-Hispanic<br>3. ARCHS: ≥ 5 years to <18 years/males and females/Black, White, Asian/Hispanic and non-Hispanic<br>4. HIV: ≥ 18 years/males and females/Black, White, Asian/Hispanic and non-Hispanic<br>5. Serostudy: ≥ 6mos to <18 years/males and females/Black, White, Asian/Hispanic and non-Hispanic, and pregnant women >18 years and newborns

**Geography:**<br>Southeast Louisiana
**Current/Target number enrolled:**<br><br>1. TUCAIN: 48/1250<br>2. CSI: 191/3240<br>3. ARCHS: 20/150<br>4. HIV: 41/50<br>5. Serostudy: 4671/20,000
**Data collection period:**<br>1. TUCAIN: 09/01/20 to 08/31/24<br>2. CSI: 04/15/20 to 08/31/24<br>3. ARCHS: 01/04/21 to 12/31/21<br>4. HIV: 06/01/21 to 08/31/24<br>5. Serostudy: 04/15/20 to 08/31/24
**Data collection intervals:**<br>1. TUCAIN: Day 0 and then every 6 months + after each immune event* 1 month and 3 mos.<br>2. CSI: Day 0, 1 month, 2 mos., 4 mos., 6 mos., then q 6 mos. + after each immune event* 1 month and 3 mos.<br>3. ARCHS: Day 0 only<br>4. HIV: Day 0, 1 month, 2 mos., 4 mos., 6 mos., then q 6 mos. + after each immune event* 1 month and 3 mos.<br>5. Serostudy: Day 0 only<br><br>*Immune Event = completion of primary vaccine series, receipt of booster dose OR SARS-CoV-2 infection
**Data collection methods:**<br><br>1. TUCAIN: electronic health record data abstraction<br>2. CSI: patient reported outcomes via survey, in person visits<br>3. ARCHS: patient reported outcomes via survey, in person visits<br>4. HIV: patient reported outcomes via survey, in person visits<br>5. Serostudy: electronic health record data abstraction
**Data elements:**<br><br>1. TUCAIN: age, race, ethnicity, zip code comorbidities, type of cancer, cancer treatment, COVID-19 vaccination hx, SARS-CoV-2 infection, symptoms, treatment, hospitalization<br>2. CSI: age, race, ethnicity, zip code, comorbidities, COVID-19 vaccination hx, SARS-CoV-2 infection, symptoms, treatment<br>3. ARCHS: age, race, ethnicity, zip code, comorbidities<br>4. HIV: age, race, ethnicity, zip code, comorbidities, COVID-19 vaccination hx, SARS-CoV-2 infection, symptoms, treatment, exposures, impact across personal and social domains<br>5. Serostudy: age, race, ethnicity, zip code, comorbidities
**Specimens collected:**<br><br>1. TUCAIN: serum, plasma, peripheral mononuclear cells (PBMCs), stored for future use<br>2. CSI: serum, plasma, PBMCs, stored for future use<br>3. ARCHS: serum, plasma, stored for future use<br>4. HIV: serum, plasma, PBMCs), stored for future use<br>5. Serostudy: serum, plasma, stored for future use

Serology/other assays:

1. TUCAIN: anti-SARS-CoV-2 N, Spike, RBD IgG ELISA, SARS-CoV-2 Spike pseudovirus neutralization assay, T cell studies
2. CSI: anti-SARS-CoV-2 N, Spike, RBD IgG ELISA, SARS-CoV-2 Spike pseudovirus neutralization assay, T cell studies
3. ARCHS: anti-SARS-CoV-2 N, Spike, RBD IgG ELISA, SARS-CoV-2 Spike pseudovirus neutralization assay, T cell studies
4. HIV: anti-SARS-CoV-2 N, Spike, RBD IgG ELISA, SARS-CoV-2 Spike pseudovirus neutralization assay, T cell studies, CD4 count
5. Serostudy: anti-SARS-CoV-2 N, Spike, RBD IgG ELISA, SARS-CoV-2 Spike pseudovirus neutralization assay, T cell studies

**Project Title:** Adaptive Immunity and Persistent SARS-CoV-2 Replication
**Grant number:** 1U01CA260462
**Primary contact:**<br><br>Swetha Pinninti, MD<br>Affiliations: University of Alabama at Birmingham<br>Address: CHB 114, 1600 6th Avenue South, Birmingham, AL 35233<br>Phone number: 205-638-2643<br>Email: spinninti@uabmc.edu
**All key collaborators:**<br><br>Name: Suresh B. Boppana, MD<br>Affiliation(s): University of Alabama at Birmingham<br>Email: sbboppana@uabmc.edu<br><br>Name: William J. Britt, MD<br>Affiliation(s): University of Alabama at Birmingham<br>Email: wbritt@uabmc.edu<br><br>Name: Sunil K. Pati, PhD<br>Affiliation(s): University of Alabama at Birmingham<br>Email:sunilkpati@uabmc.edu<br><br>Name: Misty Latting, BS<br>Affiliation(s): University of Alabama at Birmingham<br>Email:mlpurser@uabmc.edu
**Institution(s):**<br><br>University of Alabama at Birmingham
**Project Aims:**<br><br>The objective of the study is to define adaptive immune responses to SARS-CoV-2 quantitatively in a pediatric population with varying levels of immune responsiveness and to stratify these responses in terms of control of virus shedding from the upper respiratory tract.
**Primary study design:**<br><br>Case-control study
**Study population:**<br><br>Children with hematologic and solid organ malignancies on chemotherapy or other immunomodulatory treatments with acute SARS-CoV-2 infection enrolled as cases and followed prospectively. Controls include children without underlying conditions or immune compromise with acute SARS-CoV-2 infection.
**Age/Sex/Race/Ethnicity:**<br><br>Children between 3 months and 18 years of age. Sex, racial and ethnic composition will be representative of the oncology patient population at the Children's of Alabama.

Geography:

Most of the participants (80%) are residents of Alabama. A smaller proportion of children will come from other states bordering Alabama.

Current/Target number enrolled:

Number currently enrolled and target: Cases – 61/100; Controls: 40/200

Data collection period:

September 2020 to August 2023

Data collection intervals:

0, 1, 3, and 6 months for blood collection and weekly NP swab collection until x2 consecutive negative PCR results for cases.

Data collection methods:

In-person visits and electronic health record abstraction

Data elements:

Age, sex, race, ethnicity, comorbidities, symptom onset and resolution, COVID-19 exposures, severity of infection and disease, days of hospitalization, ICU admission, vasopressor support, oxygen requirement

Specimens collected:

Nasopharyngeal, nasal, and saliva swabs, and peripheral blood specimens. Blood samples are processed to separate serum and mononuclear cells. Specimens are archived at -80ºC for future studies.

Serology/other assays:

Serological assays: Binding antibodies against RBD, S and N proteins of SARS-CoV-2, S antibodies against seasonal coronaviruses. Neutralizing antibody assays using ACE2 binding inhibition, and pseudovirus. Antibodies against common antigens such as tetanus toxoid.
Detection of SARS-CoV-2 using the RT-PCR assay

Project Title: The DISCOVAR Study: Disparities in Immune Response to SARS-CoV-2 in Arkansas

Grant number: 1U01CA260526-01

Primary Contact: Wendy N. Nembhard, PhD, MPH
University of Arkansas for Medical Sciences, Fay W Boozman College of Public Health
Department of Epidemiology
4301 West Markham Street, Slot #820, Little Rock, Arkansas 72223
Phone number: 501-614-2145
Email: wnnembhard@uams.edu

Name: Joshua Kennedy, MD
Affiliation(s): University of Arkansas for Medical Sciences, College of Medicine, Department of Pediatrics and University of Arkansas for Medical Sciences, College of Medicine Department of Internal Medicine
Email: kennedyjoshua@uams.edu

Name: Karl Boehme, PhD
Affiliation(s): University of Arkansas for Medical Sciences, College of Medicine, Department of Microbiology and Immunology; Winthrop P. Rockefeller Cancer Institute, University of Arkansas for Medical Sciences, Little Rock, Arkansas; Center for Microbial Pathogenesis and Host Inflammatory Responses, Little Rock, Arkansas
Email: KWBoehme@uams.edu

Name: Namvar Zohoori, MD, PhD, MPH
Affiliation(s): University of Arkansas for Medical Sciences, Fay W Boozman College of Public Health Department of Epidemiology and the Arkansas Department of Health
Email: Namvar.Zohoori@arkansas.gov

Name: Benjamin C. Amick, PhD
Affiliation(s): University of Arkansas for Medical Sciences, Fay W Boozman College of Public Health Department of Epidemiology
Email: BCAmick@uams.edu

Name: Craig Forrest, PhD
Affiliation(s): University of Arkansas for Medical Sciences, College of Medicine, Department of Microbiology and Immunology; Winthrop P. Rockefeller Cancer Institute, University of Arkansas for Medical Sciences, Little Rock, Arkansas; Center for Microbial Pathogenesis and Host Inflammatory Responses, Little Rock, Arkansas
Email: JCForrest@uams.edu

Name: Ruofei Du, PhD
Affiliation(s): University of Arkansas for Medical Sciences, Fay W Boozman College of Public Health Department of Biostatistics
Email: RDu@uams.edu

Institution(s): University of Arkansas for Medical Sciences, Fay W Boozman College of Public Health

Project Aims:

Aim 1: Determine the serological response to SARS-CoV-2 infection over time by race/ethnicity among RT-PCR confirmed, positive adult Arkansans.

Aim 2: Determine the durability of the serological response to SARS-CoV-2 infection over time by race/ethnicity among RT-PCR confirmed positive adult Arkansans.

Aim 3: Determine the influence of psychosocial and behavioral factors on the serological response over time to SARS-CoV-2 infection by race/ethnicity among RT-PCR confirmed positive adult Arkansans.

**Primary study design:**<br><br>We will conduct a 5-year population-based, prospective cohort study in Arkansas between November 1, 2020, and September 30, 2025. The study population will consist of approximately 600 NH white, NH black and Hispanic adults >=18 years old, RT-PCR confirmed COVID-19 positive adults.
**Study population:**<br><br>The study population will consist of NH white, NH black and Hispanic adults, >=18 years old RT-PCR–confirmed COVID-19–positive adults (presymptomatic, asymptomatic, or symptomatic) in Arkansas between June 2021 and April 2022.
**Age/Sex/Race/Ethnicity:**<br>The populations included will consist of adult men and women aged 18 years or older at enrollment, an English or Spanish speaker and self-identified as Non-Hispanic black, Hispanic, or Non-Hispanic white.
**Geography:** State of Arkansas
**Current/Target number enrolled:** 450-600
**Data collection period:**<br><br>The data collection period will be from June 2021 to December 2025.
**Data collection intervals:**<br><br>Consenting study subjects will be asked to complete a computer- assisted telephone or video interview and biospecimen collection at baseline (time 0), and then 1, 2, 3, and 6 months later; thereafter every 6 months until 48 months post testing.
**Data collection methods:**<br><br>The data collection methods will include patient reported outcomes via telephone or video interview, in person data collection and electronic health record data abstraction.
**Data elements:**<br><br>Types of data elements to be collected will include ethnicity, age, race, height, weight, comorbidities, complete medical history, socioeconomic information, stress, anxiety, depression, loneliness, social support, daily functioning, perceived racism and discrimination, access to healthcare.
**Specimens collected:**<br><br>A non-fasting venous blood sample (5mL) will be collected and a dried bloodspot card for each participant.
**Serology/other assays:**<br><br>antibodies

**Project Title:** Enhancing racial and ethnic diversity in COVID-19 immunology research participation through storytelling (COVIDStory)
**Grant number:** U01CA261276
**Primary contact:**<br><br>Ann M. Moormann, PhD, MPH<br>Affiliations: University of Massachusetts Chan Medical School, Department of Medicine<br>Address: 364 Plantation Street, LRB 313, Worcester, MA 01605 USA<br>Phone number: +1 508 856 8826<br>Email: ann.moormann@umassmed.edu<br>Conflicts of Interest: None
**Other key collaborators:**<br><br>Name: Sarah Forrester, PhD<br>Affiliation(s): University of Massachusetts Chan Medical School, Department of Population and Quantitative Health Sciences<br>Email: sarah.forrester@umassmed.edu<br><br>Name: Raquel Binder, PhD MPH<br>Affiliation(s): University of Massachusetts Chan Medical School, Department of Medicine<br>Email: raquel.binder@umassmed.edu<br><br>Name: Catherine Forconi, PhD<br>Affiliation(s): University of Massachusetts Chan Medical School, Department of Medicine<br>Email: catherine.forconi@umassmed.edu<br><br>Name: Jennifer Batista<br>Affiliation(s): University of Massachusetts Chan Medical School, Department of Population and Quantitative Health Sciences<br>Email: jennifer.batista@umassmed.edu
**Institution(s):** University of Massachusetts Chan Medical School, Worcester, MA
**Project Aims:**<br><br>(1) Test the effectiveness of the COVIDStory intervention on improving participation of Black and Hispanic participants in COVID-19 immunology research through a randomized controlled trial.<br><br>(2) Determine SARS-CoV-2 seroprevalence, vaccine coverage, and characterize the SARS-CoV-2 immunological responses among the Black and Hispanic participants.<br><br>(3) Measure the association between (i) social determinants of health (SDoH) and seroprevalence; (ii) SDoH and vaccine coverage; and (iii) SDoH and vaccine hesitancy.<br><br>(4) Determine the presence, decay rate and neutralizing activity of anti-SARS-CoV-2 antibodies among diverse study participants. Re-assess factors that contribute to study attrition.
**Primary study design:**<br><br>The study will recruit up to 1,920 participants based on a community-based respondent-driven sampling (RDS) method. RDS, combines "snowball sampling" (getting individuals to refer those they know, these individuals in turn refer those they know and so on) with a mathematical model that weights the sample to compensate for the

fact that the sample was collected in a non-random way. Once participants agree to join the study, they will be asked to fill out demographic, medical history, vaccine hesitancy, and COVID19 impact surveys and are presented with either COVIDStory intervention or informational only videos. Then they will be asked if they want to participate in a COVID-19 research serology study. We expect the number of participants willing to participate in COVID research to be higher among those who have seen COVID stories. Finally, we will collect blood samples and screen them for SARS-CoV-2 antibodies (N, S, RBD IgG and IgA among others) from the individuals who agree to participate in the COVID-19 research serology study.

Study population: Black and Hispanic persons in the greater Worcester MA area.

Age/Sex/Race/Ethnicity: Black and Hispanic female and male adults.

Geography: Greater Worcester MA area

Current/Target number enrolled:

Max. 1,920

Data collection period:

October 2021 to August 2022

Data collection intervals:

Started in October 2021 and potentially 6 months later

Data collection methods:

Self-reported data collection via Qualtrics and RedCap surveys

Data elements:

Age, ethnicity, race, education, sex, living situation, income, comorbidities, economic impact of COVID-19, vaccine perception, COVID-19 vaccination/symptom/infection history

Specimens collected:

200-400 microliters blood via finger prick (~10 drops), incl. plasma and cell pellet for future use.

Serology/other assays:

ELISA and/or LUMINEX screening for SARS-CoV-2 N, S, RBD IgG and IgA antibodies among other common viral infections such as the common human CoVs (OC43, LN63, 229E, and HKU1) , SARS-COV-2 variants of concern (VOCs) influenza, EBV, and CMV.

**Project title:** Serological Sciences Network Capacity Building Center, Leidos Biomedical RFP #S20-119
**Grant number:** Subcontract 21X091 – F1
**Primary contact:** Bharat Thyagarajan, MBBS, PhD, MPH<br>University of Minnesota<br>1-136 Moos Tower, 515 Delaware Street SE, MMC 609, Minneapolis, MN 55455<br>Office: (612)624-1257; Email:thya0003@umn.edu
**Other key investigators:**<br>Amy B. Karger, Stefani N. Thomas and Luca Schifanella<br>University of Minnesota<br>Emails: karge026@umn.edu; stefanit@umn.edu; schif184@umn.edu
**Project Aims:**<br>Determine (a) proportion of vaccinated individuals who sero-convert among immunocompromised populations, (b) durability of vaccine response in immunocompromised individuals, and (c) immune response to booster doses in immunocompromised populations
**Study Design:**<br>Prospective cohort design focused on health care system-based recruitment of immunocompromised individuals.
**Study Population:**<br>Our primary focus is to study immunocompromised populations. We are specifically interested in studying three distinct groups of immunocompromised patients<br>(a) Cancer survivors: Both patients actively undergoing systemic cancer treatments and long-term cancer survivors. Patients with all types of cancer (with the exception of non-melanoma skin cancer) and all stages are eligible to participate.<br>(b) Transplant recipients: This includes both hematopoietic cell transplant recipients (autologous and allogeneic HCT recipients and solid organ transplant recipients.<br>(c) HIV positive individuals<br><br>In addition, we will recruit a generally healthy population group as a control group for this study.
**Age/Sex/Race/Ethnicity:**<br>Only adults ≥ 18 years are eligible to participate in this study.<br>All sexes, races and ethnicity are eligible to participate in this study.
**Geography:** Midwest region
**Current/Target number enrolled:**<br>Recruitment expected to start on June 1, 2021<br>Total planned recruitment: 2000 individuals (~600 patients in each of the three immunocompromised groups and ~300 healthy controls)
**Data Collection Period:**<br>Start Date: June 1, 2021<br>End Date: May 31, 2023
**Data Collection intervals:**<br>Baseline: Pre-vaccination sample<br>Follow up: 1-3 months post vaccine and every 6 months thereafter for 2 years.
**Data Collection Methods:**<br>Survey data and EHR data abstraction.

**Common Data Elements:**<br>• Demographic information: Age, race, sex, ethnicity<br>• Medical history: Cancer, HIV, transplant related questions<br>• General medical history: Comorbidities (obesity, hypertension, CVD, diabetes etc.)<br>• Risk behaviors (e.g. smoking, alcohol intake)<br>• COVID immunization history<br>• COVID risk factors (e.g. travel history, exposure to known positive cases etc.)<br>• COVID medical history (e.g. hospitalization etc.)<br>• Pandemic impact on healthcare, finances and well being
**Specimens collected:** Serum, plasma, cryopreserved PBMCs
**Serology/other assays:**<br>• Measure total antibody levels using the Roche spike and nucleocapsid antibody methods, and quantify IgG spike titers with a home brew ELISA assay<br>• Measure RBD specific B cell response in a subset of samples.<br>• T cell assays currently being evaluated.

**Project Title:u**North Carolina SeroNet Center for Excellence
**Grant number:** U54 CA260543
**Primary contact:**<br><br>Natalie M Bowman, MD, MPH, MPhil<br>Affiliations: UNC School of Medicine, Division of Infectious Diseases<br>Address: CB#7030, Bioinformatics Building, 2nd floor, Mason Farm Road, Chapel Hill NC 26599-7030<br>Phone number: 919-843-4385<br>Email: nbowman@med.unc.edu<br><br>Note: This is not the grant PI. Grant PIs are Shannon Wallet and Ralph Baric.
**Other key collaborators:**<br><br>Name: Shannon Wallet<br>Affiliation(s): UNC School of Dentistry, Department of Oral and Craniofacial Health Sciences<br>Email: spop@email.unc.edu<br><br>Name: Ralph Baric<br>Affiliation(s): UNC Gillings School of Global Public Health, Department of Epidemiology<br>Email: rbaric@email.unc.edu<br><br>Name: Alena Markmann<br>Affiliation(s): UNC School of Medicine, Division of Infectious Diseases<br>Email:Alena.Markmann@unchealth.unc.edu<br><br>Name: Jessica Lin<br>Affiliation(s): UNC School of Medicine, Division of Infectious Diseases<br>Email: linjt@email.unc.edu
**Institution(s):**<br>University of North Carolina at Chapel Hill
**Project Aims:**<br>The UNC SeroNet has multiple projects embedded within, most of which focus on immunology of SARS-CoV-2. Human samples for these studies are obtained from multiple cross-sectional and longitudinal studies based both in the hospital and in the community, as described below.<br><br>From UNC's SeroNet grant application, the overall goals of project are:<br>1) characterize the immune responses elicited to SARS-CoV2 infection<br>2) understand the mechanisms driving the serological, humoral, and cellular immune responses<br>3) determine modifiers of the serologic memory<br>4) determine the serological correlates of disease pathogenesis, and protection against future infection.<br>There are three projects under the UNC Seronet. **Project 1** will characterize the breadth and potency of polyclonal neutralizing antibody responses as well as determine the kinetics, magnitude, and durability of the type-specific and cross neutralizing responses in both the systemic and mucosal compartments. **Project 2** will determine the durability and the breadth of anti-SARS-CoV-2 serum antibodies and memory B-cells generated among convalescent plasma donors as well as determine the effect of convalescent plasma on the innate, adaptive and antibody repertoire in recipients. **Project 3** will reveal innate immune signatures as a function of serology across the span of natural disease, as well as identify signatures which promote development of protective vs. pathogenic antibody repertoires, while delineating mechanisms of antibody mediated activation and suppression of innate immune function which drives severe vs. mild disease respectively.

**Primary study design:**<br>There are several studies under the UNC SeroNet umbrella:<br>OBS-C: This is an observational longitudinal cohort of SARS-CoV-2 infected subjects designed to characterize clinical and virological aspects of infection over time and to establish a biorepository of various biospecimens from infected subjects.<br><br>COFF-NC: The study was designed to describe epidemiology and determine risk factors for SARS-CoV-2 infection in farm and food processing workers in North Carolina. Any adult who had worked at least 2 weeks in one of these industries since the beginning of the COVID-19 pandemic was eligible to participate; their household members aged 1 year and up were also invited to participate. Subjects were followed monthly with for nasal, saliva, and blood sampling, and data was collected about individual, household, and workplace risk factors for infection.<br><br>COHOST: The CO-HOST Study evaluated SARS-CoV-2 transmission in the household of individuals who tested positive and quarantined at home. Index cases were recruited after testing and were visited between 3-4 times at their private homes using a mobile unit van and returned to the Respiratory Diagnostic Center for the final study visit. NP swabs were collected weekly, and serology was performed at 28 days. Questionnaire and symptom data were collected to characterize clinical manifestations of infection and risk factors for household transmission.<br><br>Remnant samples: This study was a serial cross-sectional study to estimate seroprevalence of SARS-CoV-2 in North Carolina over time. Remnant serum and plasma samples from patients 5 years and older undergoing laboratory testing for any reason were collected from clinical laboratories of four UNC-affiliated hospitals. Data were deidentified and waiver of consent was provided.
**Study population:**<br>OBS-C: People of any age with a proven diagnosis of SARS-CoV-2 infection.<br><br>COFF-NC: Adult workers at farms or food processing facilities (primarily meatpacking) and their household members at least 12 months of age.<br><br>COHOST: Adults recently infected with SARS-CoV-2 by NP swab and household members at least 12 months of age<br><br>Remnant samples: Ages 5-99 who had remnant plasma or serum collected at UNC-affiliated hospitals from April 2020 to present.
**Age/Sex/Race/Ethnicity:**<br>OBS-C: All ages, both genders, mix of race/ethnicity<br><br>COFF-NC: Age range 1 year and up (mean 21 years), 47% female, 94% Latinx<br><br>COHOST: Age range 1 year and up, 55% non-Hispanic white<br><br>Remnant samples: Predominantly adult (<6% children), 56% female, racial/ethnic profile similar to population of central NC
**Geography:**<br>North Carolina, with the majority of samples from the central part of the state.
**Current/Target number enrolled:**<br>OBS-C: 41 enrolled/ongoing<br><br>COFF-NC: 224 enrolled<br><br>COHOST: 315 enrolled

Remnant samples: 9624 enrolled/ongoing
**Data collection period:**<br>OBS-C: April 2020 to present<br><br>COFF-NC: September-December 2020, may have future data collection in the same population<br><br>COHOST: April-October 2020, some follow up data in collection now<br><br>Remnant samples: April 2020 to present
**Data collection intervals:**<br>OBS-C: Day 0, 7, 14 and month 1, 3, 6, 12. Inpatients sampled more frequently until discharge.<br><br>COFF-NC: monthly<br><br>COHOST: Weekly through day 28; some with late convalescent sampling<br><br>Remnant samples: Cross-sectional
**Data collection methods:**<br>OBS-C: EMR data abstraction, interview, survey<br><br>COFF-NC: interview, survey<br><br>COHOST: interview, survey<br><br>Remnant samples: EMR data abstraction
**Data elements:**<br>OBS-C: age, sex, ethnicity, race, location, medical history, household information, COVID-related behaviors, risk factors<br><br>COFF-NC: age, sex, ethnicity, race, location, medical history, household information, COVID-related behaviors, risk factors<br><br>COHOST: age, sex, ethnicity, race, location, medical history, household information, COVID-related behaviors, risk factors<br><br>Remnant samples: age, sex, ethnicity, race, location, insurance, inpatient/outpatient, ICD-10 codes, date of discharge, recent COVID testing
**Specimens collected:**<br>Varies between studies but can include:<br>Blood (serum and/or plasma)<br>Saliva<br>Nasopharyngeal and/or nasal midturbinate<br>Tracheal aspirate<br>Sputum<br>Bronchoalveolar lavage<br>Urine<br>Exhaled breath condensate
**Serology/other assays:**<br>RBD, ELISA, SARS-CoV-2 PCR (various respiratory specimens)

**Project title:** SARS-CoV-2 correlates of protection in a Latino-origin population
**Grant number:** U01 CA260541
**Primary contact:**<br><br>Carlos Sariol, MD, PhD<br>Affiliations: University of Puerto Rico, Medical Sciences Campus<br>Address:PO Box 365067<br>Phone number:787-758-2525<br>Email:carlos.sariol1@upr.edu<br>Conflicts of Interest: None
**Co-investigators:**<br><br>Name:Marcos Lopez<br>Affiliation(s):Puerto Rico Public Health Trust, Puerto Rico Science, Technology and Research Trust and University of Puerto Rico at Humacao<br>Email: marcos.lopez11@upr.edu<br><br>Name:Daniela Weiskopf<br>Affiliation(s):La Jolla Institute of Immunology<br>Email:dweiskopf@lji.org<br><br>Name:Ana Espino<br>Affiliation(s):University of Puerto Rico Medical Sciences Campus<br>Email:ana.espino1@upr.edu<br><br>Name:James Brien<br>Affiliation(s) : Saint Louis University<br>Email:ana.espino1@upr.edu<br><br>Name:Alba Grifoni<br>Affiliation(s):La Jolla Institute of Immunology<br>Email:agrifoni@lji.org
**Institution(s):**<br><br>University of Puerto Rico Medical Sciences Campus<br>Puerto Rico Science, Technology and Research Trust<br>La Jolla Institute of Immunology
**Project Aims:**<br><br>1. Expansion of a comprehensive Infectious Diseases Repository Biobank (IDRB) and serologic diagnostic platform.<br>2. Characterization of the humoral immune response to SARS-CoV-2 virus in Puerto Rico by identifying the prevalence of antibodies to this virus and correlation of antibody titer with neutralization capabilities, quality of the neutralization, and disease outcome.<br>3. Dissection of the T cell immune phenotypes and correlation with the serological immune response, cytokine profile and disease outcome.<br>4. HLA and KIR characterization and correlation with the immune response and COVID-19 disease severity.
**Primary study design:**

We are proposing to collect about 6,000 samples per year so at the end of the five-year program we would be collect up to 30,000 samples. The study population consists of all confirmed or suspect COVID19 cases in a year. A random sample of 6,000 cases will be selected annually for serological screening representing approximately 17% of all confirmed or suspect cases expected in a year. As of Sept. 7, the estimated total number of SARS-CoV-2 cases is 35,375 (includes all cases, deceased, and recovered cases) in a population of approximately 3,193,694. The total cases per million population in Puerto Rico is 10,445 compared to 19,581 per million population in the US, however the active cases rate in PR is 11,086 per million population, compared to 7,749 per million population in the US. The symptomatic rate in Puerto Rico as in many other states and countries is unknown. Of the 6,000 cases expected annually, between 150-200 cases (2.5% to 3.3%) will be selected for further immunological tests to determine HLA typing and T cells immune response.

Study population:

Taking advantage of the local population It is anticipated that about 76% of the participants on this study will be white Latino-population and 12.5% Black/African, followed by about 9% of other Latino-origin groups (Dominican, Cuban, Colombians, Venezuelan among others) and a 4% of mixed population (American Indian, Alaskan Native, Native Hawaiian, other Pacific Islander, and others. However, inclusion will not be limited to Latinos or the ethnical groups described above. Any person willing to participate or samples collected from any individual regardless of the ethnicity will be eligible

Age/Sex/Race/Ethnicity:

Efforts will be directed to guarantee a proper representation of age, sex, gender, socioeconomic status as well as individuals with comorbidities including but not limited to obesity, cancer, and autoimmune diseases.

Geography:

Puerto Rico is the easternmost island of the Greater Antilles chain, it lies approximately 50 miles (80 km) east of the Dominican Republic, 40 miles (65 km) west of the Virgin Islands, and 1,000 miles (1,600 km) southeast of the U.S. state of Florida. It is situated in the northeastern Caribbean Sea, its northern shore facing the Atlantic Ocean. Two small islands off the east coast, Vieques and Culebra, are administratively parts of Puerto Rico, as is Mona Island to the west. It is roughly rectangular in shape, extending up to 111 miles (179 km) from east to west and 39 miles (63 km) from north to south.

Current/Target number enrolled:

Currently enrolled patients: 4354 patients
Target (in five years) ~30,000 patients

Data collection period:

Started in November 2020 and will end July 2025

Data collection intervals:

We are recruiting COVID-19 positive patients. We also have cohorts of vaccinated people. Samples are taken before vaccination and two weeks after finishing the vaccination regime. Follow up samples after 3 and 6 months are also taken.

Data collection methods:

We do electronic health record data abstraction, patient reported outcomes via survey, interviews, and in person visits.

**<u>Data elements:</u>**<br><br>Age, race, ethnicity, comorbidities, symptoms, date of positive test, vaccination, trademark of vaccine, date of vaccination.
**<u>Specimens collected:</u>**<br><br>NP swabs and whole blood is collected. Samples are stored in a biorepository for future use.
**<u>Serology/other assays:</u>**<br><br>We use an in house developed ELISA-based IgM/IgG tests. We use FDA-EUA-RT-PCR tests as well.

**Project Title:** Highly Multiplexed Immuno-Serological Assays for COVID19 in Patients with Hematologic Malignancies
**Grant number:** U01CA260507
**Primary contact:**<br><br>Rong Fan, Ph.D.<br>Affiliations: Yale University<br>Address: 55 Prospect St, MEC 213, New Haven, CT 06520, U.S.A.<br>Phone number: 1-203-432-9905<br>Email: rong.fan@yale.edu<br>Conflicts of Interest: R.F. is scientific founder and advisor of IsoPlexis, Singleron Biotechnologies, and AtlasXomics. The interests of R.F. were reviewed and managed by Yale University Provost's Office in accordance with the University's conflict of interest policies.
**Other key collaborators:**<br><br>Name: Stephanie Halene, MD, PhD<br>Affiliation(s): Yale School of Medicine<br>Email:stephanie.halene@yale.edu<br><br>Name: Dongjoo Kim, PhD<br>Affiliation(s): Yale University<br>Email: dongjoo.kim@yale.edu<br><br>Name: Giulia Biancon, PhD<br>Affiliation(s): Yale School of Medicine<br>Email: giulia.biancon@yale.edu<br><br>Name: Jennifer VanOudenhove, PhD<br>Affiliation(s): Yale School of Medicine<br>Email: jennifer.vanoudenhove@yale.edu
**Institution(s):**<br><br>Yale University
**Project Aims:**<br><br>Aim 1. Develop an immuno-serologic microchip assay for high-throughput measurement of 32 plasma protein markers including SARS-Cov-2 antibodies.<br>Aim 2. Develop a single-cell multi-protein assay for evaluating cellular immune response.<br>Aim 3. Comprehensive immuno-serological and cellular response analyses of a cohort of COVID19 patients with hematological malignancies.
**Primary study design:**<br><br>Retrospective cohort study of patients with hematological malignancies infected by SARS-CoV-2 and controls. Prospective cohort study of patients with hematological malignancies in response to COVID-19 vaccination. These patients will be followed longitudinally with repeated serologic measures from the time of vaccination.
**Study population:**<br><br>Adult (age ≥18) patients with hematological malignancies or non-hematological disease controls.

**Age/Sex/Race/Ethnicity:**<br><br>Age: median 60, range 21-86 Gender: 63.8% male.<br>Plan to enroll participants from all major races and ethnic groups, in particular, African Americans in the New Haven area.
**Geography:**<br><br>New Haven County – Study catchment area consists of several neighborhoods with participants that can be monitored longitudinally and populations of diverse socioeconomic status.
**Current/Target number enrolled:**<br><br>~100 currently enrolled, with a total target of ~300 participants.
**Data collection period:**<br><br>11/01/2020 – 10/31/2025
**Data collection intervals:**<br><br>Samples will be collected at pre-vaccine, and 1 month, 3 months, 12 months and 24 months post vaccination for patients who consented to participate. For a subset of patients, we are able to collect pre-vaccine samples, however pre-vaccine data time point are difficult to collect due to the fast rollout of the vaccinations in the state of Connecticut winter 2020/21.
**Data collection methods:**<br><br>Patient data collection is conducted via in person visits with our hematology physician team as well as abstraction from the EMR/EHR.
**Data elements:**<br><br>The metadata elements include patient age, sex, race, disease type, disease stage and duration, medications/treatment, vaccination status and duration, current and prior treatments, as well as vaccine type, prior or current COVID-19 status. The assay data elements include multiplex serology test and single-cell immune profiling data from patients.
**Specimens collected:** Plasma, Serum, and PBMC.
**Serology/other assays:**<br><br>Microfluidic barcode chip for high-plex serology assay.<br><br>Microfluidic barcode chip for high-plex plasma protein assay.<br><br>CodePlex assay for multiplex cytokine assay commercially available at IsoPlexis<br><br>IsoCode assay of single-cell cytokine signature commercially available at IsoPlexis<br><br>Single-cell RNA-seq commercially available at 10x Genomics<br><br>Single-cell TCR/BCR sequencing available at 10x Genomics<br><br>CyTOF assay for multiplex immunophenotyping commercially available at Fluidigm
